# Explicit Mechanistic Operators in Predictive Virtual Cells: Evidence, Failure Modes, and a Minimum Falsification Framework for Single-Cell Perturbation Prediction

**DOI:** 10.3390/cells15181711

**Published:** 2026-09-20

**Authors:** Mikołaj Stańczak, Sarfaraz K. Niazi

**Affiliations:** 1 School of Mathematical and Computer Science, Heriot-Watt University, Edinburgh EH14 4AS, UK; 2 College of Pharmacy and Pharmaceutical Sciences, Washington State University, Spokane, WA 99202, USA

**Keywords:** predictive virtual cell, mechanistic operator, neural differential equation, cellular foundation model, perturbation prediction, out-of-distribution generalization, identifiability, model discrepancy, unfolded protein response, falsification

## Abstract

The term virtual cell now covers objects ranging from curated biochemical simulators to atlas-trained neural representations, yet the decisive empirical question is narrower: whether a model built to obey known biochemical rules predicts the effects of new drugs or genetic changes better than an otherwise identical model that lacks them. Formally, this Perspective asks whether a biologically specified mechanistic operator improves out-of-distribution prediction of single-cell perturbation responses beyond strong simple, relational, transport-based, and learned-dynamical comparators. Recent benchmarks show that rankings depend on partition, statistical unit, effect-size distribution, gene set, and metric, and that several deep and foundation-model approaches fail to exceed no-change, mean-effect, additive, or linear controls. Set-based, knowledge-graph, transport, and learned state-equation models can improve defined tasks. No identified study isolates the incremental value of a biologically specified operator against late fusion, a capacity-matched static model, a capacity-matched learned-dynamical operator, and topology-rewired controls under one locked evaluation. Six source categories are distinguished, with the operator category divided into biologically specified and learned-dynamical subclasses. A minimum falsification framework is specified for the unfolded protein response with a declared capacity ledger, endpoint times chosen by a prespecified Stage 1 rule, and a locked comparator set. The framework is prospective: no new data, model implementation, or performance result is reported, and every threshold is a design choice awaiting empirical test.

## 1. Introduction

### 1.1. Operational Definition and Boundary

A virtual cell may mean a spatial simulation environment, a curated whole-cell reconstruction, a learned cellular representation, a perturbation-response model, or a patient-specific digital twin. These objects share the goal of representing cellular behavior, but they do not share an estimand, an observation model, or a validation standard. The Virtual Cell software environment was built to simulate biochemical systems within measured geometries, whole-cell reconstructions integrate many gene products and mathematical formalisms within one organism, and the current artificial intelligence program emphasizes learned representations and queryable models trained on large cellular corpora [1,2,3,4,5].

This Perspective adopts a deliberately narrow definition. A predictive virtual cell is a model that estimates a measurable response to a specified genetic or chemical intervention within a defined cellular context and can be scored against held-out observations. The response considered here is single-cell or replicate-level transcriptomic change. Other modalities enter only when they constrain the mechanistic state or test the predicted mechanism. Whole-cell models, tissue-scale simulations, clinical decision support, and longitudinal individual calibration fall outside this definition because they carry different and more demanding evidentiary requirements [5,6].

### 1.2. Out-of-Distribution Prediction Is Not One Task

The phrase out of distribution is often treated as a single property, although it covers several distinct transport problems. Predicting an unseen perturbation within a familiar cell line tests whether the model can associate interventions with responses. Predicting a familiar perturbation in a new cell type or donor tests transfer of response across biological context. Holding out dose, timing, combinations, laboratory, or assay platform each removes a different source of information. A single composite score hides these distinctions and can present success along an easier axis as general cellular prediction. Evaluation must therefore name the held-out unit and report each axis separately [7,8,9,10].

The observation target must also be explicit. A model may predict the mean log fold change across genes, a distribution of individual cell states, the probability of differential response, a pathway score, or a phenotypic endpoint. These targets are not interchangeable. Single-cell measurements are destructive and typically unpaired, so an apparent trajectory from an untreated to a treated cell reflects population-level coupling rather than an observed longitudinal path. Distributional methods such as neural optimal transport are built for this setting, whereas mean-response models answer a different question [11,12,13].

### 1.3. Central Question

In plain terms, the core question is whether a model that is built to obey known biochemical rules can predict the effects of new drugs or genetic changes better than a standard black-box model of the same size, trained on the same data with the same tuning and computing budget. A biologically specified operator is the component that carries those rules: a small set of equations whose variables are named molecules or activities, whose parameters are rates or affinities with plausible ranges, and whose form is fixed from prior knowledge before any data are fitted. Mechanistic predictive gain, defined in Section 6.6, is the proposed measure of what that component adds: the improvement in held-out prediction relative to the strongest matched alternative that is identical in every other respect but lacks the biological rules. The question matters beyond the specialist community because mechanistic structure is widely assumed to make virtual-cell predictions more trustworthy and more transferable to new cell types and treatments. If that assumption is correct, it should be demonstrable under a fair comparison, and if it is not, the effort spent encoding mechanism is better directed at calibration, experimental design, and hypothesis generation, where its value is not in dispute. The definitions that follow are formal versions of these three ideas.

The primary question is whether a biologically specified mechanistic operator supplies predictive information beyond what strong simple, relational, transport-oriented, task-specific, and learned-dynamical models already contain. Operators form one parent category with two subclasses. A biologically specified operator is a state equation, event process, rule-based simulator, or constrained optimization problem whose states, parameters, rules, objectives, and constraints carry declared biological semantics fixed before fitting. A learned-dynamical operator is a state equation of the same mathematical type whose structure and parameters are estimated from data and read biologically after fitting, as in a neural ordinary differential equation or a stationary diffusion with neural drift. A knowledge graph, coexpression graph, pathway mask, causal regularizer, or topological penalty belongs to neither subclass, because none of them specifies how a state evolves.

The question is incremental rather than a verdict on the scientific value of mechanism. Mechanistic models organize knowledge, expose contradictions, estimate latent quantities, support experimental design, and state conservation laws even when they do not improve a transcriptomic benchmark. The hypothesis under test is narrow and empirical: after fixing the split, observations, tuning budget, total trainable capacity, and compute, does a biologically specified operator improve a prespecified held-out endpoint more than a capacity-matched unstructured model, a capacity-matched learned-dynamical operator, a rewired operator, or late fusion of the same simulated features? This framing keeps explanatory plausibility separate from demonstrated predictive validity [14,15,16].

### 1.4. Evidence Appraisal

This article is a critical Perspective with a reproducible update strategy. It is not a systematic or scoping review and claims no exhaustive coverage. The search was first run to 29 July 2026 and was rerun and broadened on 14 August 2026 after an initial appraisal showed that dynamical formulations of perturbation response were underrepresented. The broadened strategy added neural ordinary differential equation, ordinary differential equation, stochastic differential equation, diffusion, steady-state ODE, gene regulatory network, dynamical system, Perturb-seq combined with ODE, and single-cell perturbation combined with dynamics to the operator terms, and it recovered the studies discussed in Section 3.5. Publication status and citation metadata for the studies used here were rechecked on 22 August 2026. The exact query families appear in Appendix A, and the expanded extraction records are provided in Supplementary File S1.

Sources were included when they supplied an empirical comparison, a mathematical method, a software or data standard, an experimental perturbation resource, or a credibility framework bearing directly on the central question. Application reports without a matched comparator were retained only as architectural precedents and excluded from performance claims. Peer-reviewed versions supersede preprints, and preprints are labeled at each point of material use. Every quantitative claim was checked against the primary publisher or preprint record. Because selection and extraction were carried out by one author, no ordinal evidence hierarchy is used. Evidence is instead described along the independent dimensions in Table 1 [13,17]. How possible omissions from a single-author search would bear on each class of conclusion drawn here is set out in Section 10.

### 1.5. What This Perspective Adds

Three recent syntheses cover adjacent ground. The community roadmap for building an artificial intelligence virtual cell sets priorities and opportunities but does not specify a decision rule for mechanistic coupling [5]. The review of interpretation, extrapolation, and perturbation of single cells organizes methods and estimands and is the best current map of the modeling space, yet it treats mechanistic and relational structure as parallel design choices rather than as claims requiring different controls [13]. The survey of virtual-cell models in preclinical research describes technical pathways and translation potential without fixing the comparator set that would make a translation claim testable [17]. This Perspective adds two things that none of them supplies. The first is an operator-isolation criterion that distinguishes the two operator subclasses from the five non-operator sources of biological structure and states the control required for each. The second is a single quantity, mechanistic predictive gain, defined against matched late-fusion, rewired, static-capacity, and learned-dynamical alternatives rather than against a historical model. It then gives a reproducible minimum-experiment specification with a declared capacity ledger and a prospective refutation rule.

### 1.6. Nomenclature and Conventions

**Figure 1:**
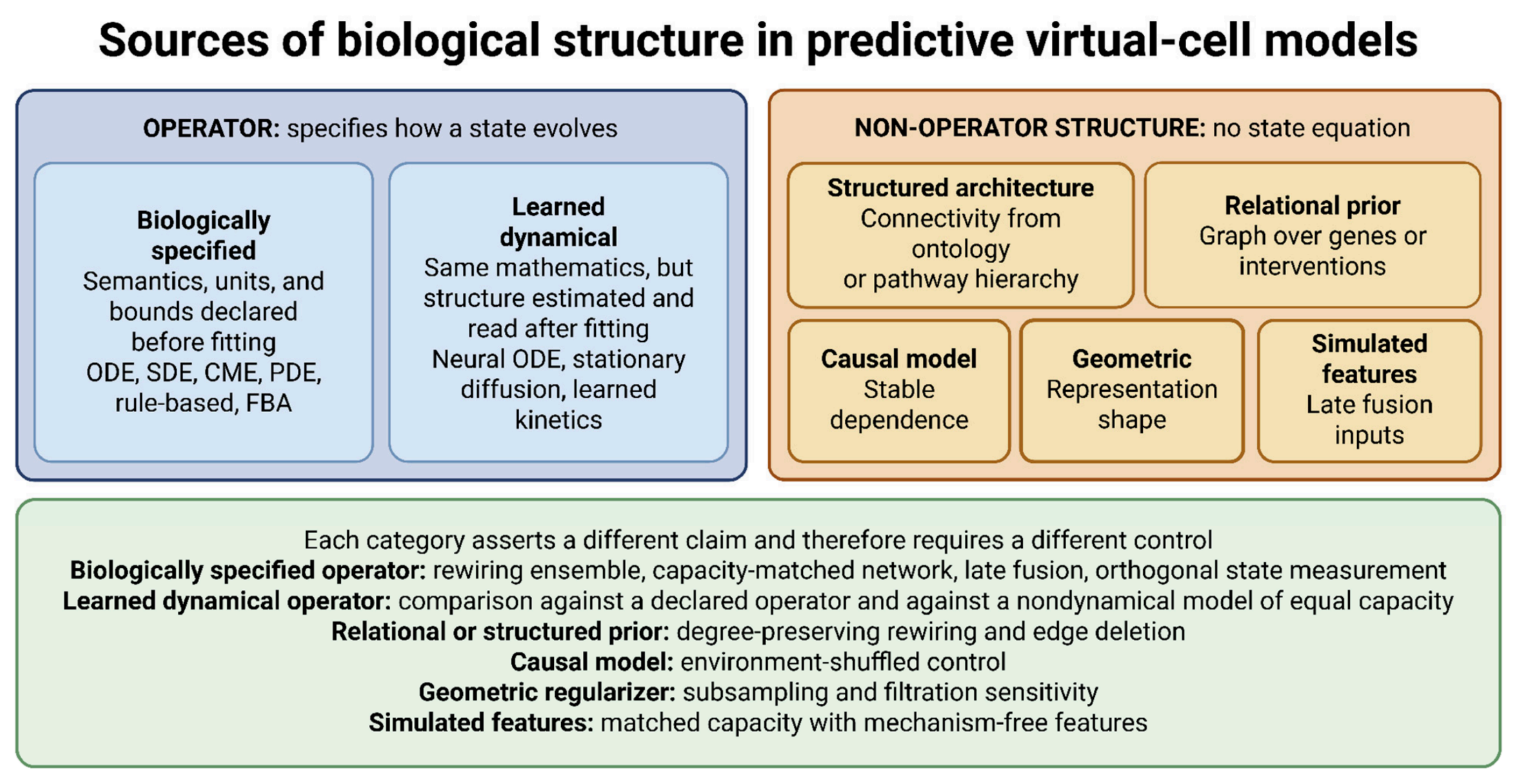
Sources of biological structure used in predictive virtual-cell models. An operator is the parent category and is divided into biologically specified and learned-dynamical subclasses. Five non-operator sources are shown separately: structured architectures, relational priors, causal models, geometric regularizers, and simulated features. These categories may be combined, but they assert different scientific claims and require different controls. Created in BioRender. Niazi, S. (2026) https://BioRender.com/ldv0x19 (accessed on 15 September 2026).

The notation is deliberately compact because reviewing hybrid models becomes difficult when one symbol serves biochemical parameters, neural weights, probabilistic parameters, and observations. Table 2 separates these entities and gives the units expected before any nondimensional transformation. The convention follows systems biology practice, in which model structure, experiments, observations, and parameter bounds are distinct reproducible objects rather than undocumented implementation details [18,19,20,21] (Figure 1).

## 2. What Counts as a Mechanistic Constraint

### 2.1. Explicit Dynamical and Optimization Operators

An operator specifies how a biological state evolves or how it is selected. Ordinary and stochastic differential equations, chemical master equations, rule-based reaction systems, reaction–diffusion systems, and constraint-based optimization problems all belong here, and the category divides into two subclasses. In a biologically specified operator, the states, parameters, rules, objectives, and constraints carry declared biological semantics, with units where the quantity is dimensional and bounds where a physical range is known, fixed before fitting rather than assigned afterwards. States may be concentrations, occupancies, activities, fluxes, discrete regulatory states, or membership in a feasible set, and parameters may be rates, affinities, thresholds, flux bounds, or objective coefficients [1,2,3,4,22,23]. In a learned-dynamical operator, the same mathematical objects are estimated from data and interpreted afterwards, which changes what the model can be held to rather than whether it is dynamical.

The scientific content of such an operator is that its quantities are biologically interpretable and are either directly observable or independently constrainable, including effective parameters and reduced-model states that are identifiable only through combinations. This is also its exposure. When a state cannot be constrained by any available measurement, when a parameter is not identifiable from the design in hand, or when a term is inserted only to improve fit, the operator retains the appearance of mechanism without the accountability. The remainder of this Perspective treats that gap as the central risk of hybrid modeling [22,23,24].

### 2.2. Biologically Structured Architectures and Relational Priors

Biologically structured neural architectures constrain connectivity using ontologies, pathway hierarchies, or curated interaction sets. Visible neural networks, biologically informed networks for cancer discovery, and knowledge-primed architectures show that such constraints can improve interpretability and sometimes accuracy [25,26,27]. They do not integrate a state equation, and their nodes are not required to obey conservation, positivity, or mass balance.

Relational priors supply graph structure over genes or interventions without constraining the functional form. Knowledge graphs, coexpression graphs, and gene ontology similarity graphs are of this type, and they underpin several of the strongest current perturbation predictors [28,29]. A relational prior asserts that related interventions have related effects, and it can be falsified by a suitably designed interventional experiment, as can a causal hypothesis. What an explicit operator adds is a further set of quantitative claims about state evolution, kinetics, conservation, or optimality that can be tested directly against measurements of the states themselves.

### 2.3. Causal, Geometric, and Simulated-Feature Structure

Causal models and invariance assumptions restrict which associations may be treated as stable across environments. Structural causal models, causal representation learning, and invariant prediction each impose testable restrictions, but they operate at the level of admissible dependence rather than of biochemical dynamics [30,31,32,33]. Geometric and topological constraints regularize the shape of a representation. Persistent homology summaries are numerically stable under small perturbations, which is a mathematical guarantee rather than evidence that two representations share a causal mechanism [34,35].

Simulated features enter through late fusion, in which a mechanistic simulator produces pathway activities, parameters, or fluxes that a downstream learner consumes without gradient flow across the operator. This design is often dismissed as weak coupling, yet it answers the prior question directly: does the mechanistic output carry predictive information at all? Because the operator and the predictor can be audited separately, late fusion also reveals whether an end-to-end model uses the mechanism or merely exploits added capacity, which is why an artificial immune-system model coupled to clinical breast cancer data remains a useful precedent for the design [15,36]. Table 3 states what each category may legitimately claim before experimental validation.

## 3. Benchmark Record for Out-of-Distribution Perturbation Prediction

### 3.1. Resources and the Prediction Estimand

Perturb-seq and related pooled screens link genetic interventions to transcriptomic readouts in thousands of cells, and chemical screens add compounds, doses, and cell lines. The Adamson unfolded-protein-response (UPR) screen, the Norman genetic-interaction study, and the genome-scale Replogle atlas have become recurring genetic benchmarks; chemical benchmarks include Sci-Plex and the much larger Tahoe-100M resource, which remains a preprint [37,38,39,40,41,42]. Harmonized collections such as scPerturb and analysis frameworks such as pertpy reduce format and metadata friction, but they do not remove differences in assay design, target engagement, dose, timing, or biological replication [41,43].

The most common prediction is a post-intervention expression profile conditioned on an untreated population and an intervention label. Some methods predict a mean profile, some transport a distribution of cells, and some estimate perturbation-specific effects after systematic variation has been removed. High correlation can arise from genes that are stable across conditions or from a global treated-versus-control difference. Transcriptome-wide analysis shows that many perturbations have small effects, which makes no-change and mean-effect controls strong by construction. Evaluation must therefore separate reconstruction of the cellular background from reconstruction of the perturbation-specific signal [44,45].

### 3.2. What the Negative Results Established

Ahlmann-Eltze and colleagues compared five pretrained cellular encoders and two other deep models against no-change, additive, and linear controls, and no deep model exceeded the simple baselines on the unseen-single and unseen-combination tasks examined. Wong and colleagues reached the same conclusion for held-out perturbations across three widely used Perturb-seq datasets and identified a label inconsistency in a curated benchmark file, which shows how preprocessing can move the apparent target. PertEval-scFM found limited and inconsistent value from zero-shot foundation-model embeddings under distribution shift, and the peer-reviewed evaluation of post-perturbation RNA sequencing reported a similarly modest advantage over simpler approaches [7,8,9,46].

These results do not show that deep or pretrained models cannot work. They show that size and pretraining alone do not deliver generalization on the endpoints evaluated, and that an adequate comparator must capture the empirical regularities available without a complex model. An additive combination of known single effects is the decisive control for combinatorial prediction; a training-set mean or no-change model is decisive when perturbation effects are small; and ridge or elastic-net models reveal whether a nonlinear architecture adds anything beyond a regularized linear map. Performance claims made without these controls cannot be reconciled with the subsequent benchmark record [7,8,44].

### 3.3. Why Rankings Change with Metric, Split, and Preprocessing

Systema showed that systematic variation between perturbed and control populations can dominate common metrics and inflate apparent perturbation-specific prediction, and it argued for heterogeneous gene panels and for reconstruction of the perturbation landscape rather than of a shared background [45]. The 2026 benchmark of 27 methods across 29 datasets and six metrics found that rankings depend on the generalization setting and identified cellular-context representation as the limiting factor [10]. scArchon, which compares nine published methods across six single-cell RNA sequencing datasets under a reproducible Snakemake pipeline, reached the same conclusion from a different direction: several tools fall below linear or control-level baselines on some datasets, and no single score or model family provides a stable overall ranking [47].

Metric orientation is a recurring source of ambiguity. Correlation across genes within a perturbation asks whether the response pattern is reproducible; correlation across perturbations within a gene asks whether a gene tracks intervention-specific variation; and correlation across all gene–perturbation pairs can be dominated by global means. Selecting differentially expressed genes from the test set leaks outcome information into scoring, and reporting thousands of cells as independent observations overstates precision when they come from one well, donor, organoid, or cell line. Pseudobulk and hierarchical methods respect the biological unit within the inferential model [48,49,50,51].

### 3.4. Gains from Set, Relational, and Transport Structure

The record improved when models encoded information aligned with the transfer problem. STATE, which remained a bioRxiv preprint at the 22 August 2026 publication-status recheck, uses a set-based architecture trained on large observational and interventional corpora and reports consistent gains over mean, linear, autoencoder, and encoder baselines in held-out cellular contexts, with the largest gains in context-diverse data. Because the result is a preprint, it should be read as a well-controlled preliminary report rather than as a performance standard, and its status should be rechecked at submission [52].

TxPert, published in *Nature Biotechnology* on 1 May 2026, is a knowledge-graph model that exceeds additive and task-specific baselines on unseen-single, unseen-combination, and cross-cell-line tasks and reports performance against split-half experimental reproducibility [28]. PrePR-CT uses cell-type-specific coexpression graphs to improve chemical-response prediction under limited data and in new cell types [53]. CMonge learns a global optimal-transport map conditioned on drug, dose, and combination and improves distributional prediction for unseen conditions [12]. What these models share is not a biochemical operator but structured information about relations among interventions, contexts, or populations, combined with evaluation designed to expose perturbation-specific effects.

### 3.5. Learned Dynamical Models of Perturbation Response

A second strand of positive results is dynamical rather than relational, and it must be represented accurately because it falls directly within the subject of this article. Latent causal diffusion frames single-cell gene expression as a stationary diffusion process observed under measurement noise, learns an explicit gene-level stochastic differential equation as the generative model, and reports more accurate prediction of distributional shifts for unseen perturbation combinations than established approaches, together with a linearization that approximates direct causal effects between genes [54]. PerturbODE models cell-state trajectories under perturbation with a neural ordinary differential equation whose parameters encode a gene regulatory network, and it predicts held-out transcription-factor overexpression more accurately than linear structural causal models on an atlas of over a thousand interventions [55]. LazyNet is a compact one-step neural ordinary differential equation for single-cell CRISPR activation and interference that operates on paired pre-treatment and post-treatment snapshots, yields multiplicative parameters with a stated interpretation, and reports competitive genome-wide accuracy under a matched one-hour training budget [56]. CLAMP, presented as a mechanistic probe of regulatory structure preserved by frozen single-cell foundation-model representations, fits a shared differentiable steady-state Hill-kinetics system to Perturb-seq endpoints, recovers a gene-space regulatory adjacency, and evaluates held-out combinatorial perturbations; the GenBio 2026 report is a non-archival workshop Spotlight [57]. Any claim that the positive results in this field contain no state equation is therefore wrong, and the evidence gap has to be stated more narrowly.

CellBox is an important earlier architectural precedent for coupling an explicit dynamical system to gradient-based parameter inference. It couples a system of ordinary differential equations for molecular and phenotypic responses to inference adapted from deep learning, and it predicts responses to untested drug combinations in a melanoma line, outperforming static network models and a neural network on transfer to unseen targets [58]. CellBox lies outside the estimand adopted in Section 1.1, because its readouts are protein, phosphoprotein, and phenotypic rather than single-cell transcriptomic, and that boundary should be stated plainly. Its variables and interventions were declared before fitting against measured signaling nodes, so within the taxonomy of Table 3, it sits closer to the biologically specified subclass than the transcriptomic models above, and it is treated separately in the paragraph that follows for that reason.

The distinction that survives is between the two operator subclasses. In latent causal diffusion, PerturbODE, LazyNet, and CLAMP, the dynamics are real and the systems are genuinely dynamical, but the regulatory structure is estimated rather than predeclared, the parameters are network weights or shared kinetic coefficients whose biological reading is extracted after fitting, the interventions enter as generic shifts, masks, or scaling terms rather than as declared biochemical actions on named terms, the states are transcript-level or latent module quantities rather than distinct molecular species, and no independent measurement of a latent state constrains the fit [54,55,56,57]. CellBox differs on the last three of these points and is limited instead by its estimand and by the absence of matched capacity and rewiring controls [58]. None of this is a defect for their stated purposes. It does mean that none of them tests whether biologically specified structure, declared in advance and constrained by orthogonal measurement, adds predictive information beyond a capacity-matched alternative, which is the comparison specified in Section 7 and Section 8.

### 3.6. Foundation Models Must Be Judged by Task

Cellular foundation models differ in tokenization, pretraining objective, corpus, and intended use. scGPT and scFoundation build general single-cell representations, Geneformer emphasizes network-aware transfer, and universal cell embedding builds zero-shot representations across tissues and species without dataset-specific fine-tuning [59,60,61,62]. The universal cell embedding model reached peer-reviewed publication in *Nature* on July 2026 and demonstrated substantial value for representation and atlas analysis. None of these achievements amounts to predicting the specific change produced by an intervention in an unseen context. Failures in perturbation prediction should not be generalized to annotation or integration, and success in annotation is not evidence of interventional prediction.

The separation between representation and response prediction also governs the analysis of pretraining contamination. A downstream test cell line may be absent from supervised fine-tuning yet present in the pretraining corpus, or a related experiment may be cataloged under a different identifier. Zero-shot claims therefore require a complete record of pretraining data, overlap assessment at accession level, corpus publication dates, and a statement of whether the test condition influenced tokenization, gene selection, or normalization. The problem is sharper for atlas-scale models because their scientific value depends on the breadth of prior exposure [51,62,63].

Two qualifications keep this separation from being overstated. First, the negative results summarized in Section 3.2 are statements about named models, corpora, tasks, and dates, not about the ceiling of the approach. Foundation models are improving quickly, and a model that learns to predict interventional responses in unseen contexts would not weaken the argument of this Perspective; it would raise the bar that a mechanistic operator must clear, because the strongest task-compatible general predictor is selected on validation data and becomes the comparator (Section 8.1). Second, the distinction between representation and response prediction describes the evidence available to date rather than the potential of the approach. Cross-context transfer is where pretrained representations are most plausibly useful: the largest reported gains of the set-based STATE preprint occurred in context-diverse data, and the 2026 multi-method benchmark identified cellular-context representation as the limiting factor [10,52]. The proposed design therefore retains a frozen encoder and a fine-tuned encoder among its comparators and reserves learned context generalization for a later multi-context stage (Section 7.7), where a pretrained representation can be tested for cross-context transfer under the same leakage controls rather than assumed to lack it.

### 3.7. Evidence Synthesis

**Figure 2:**
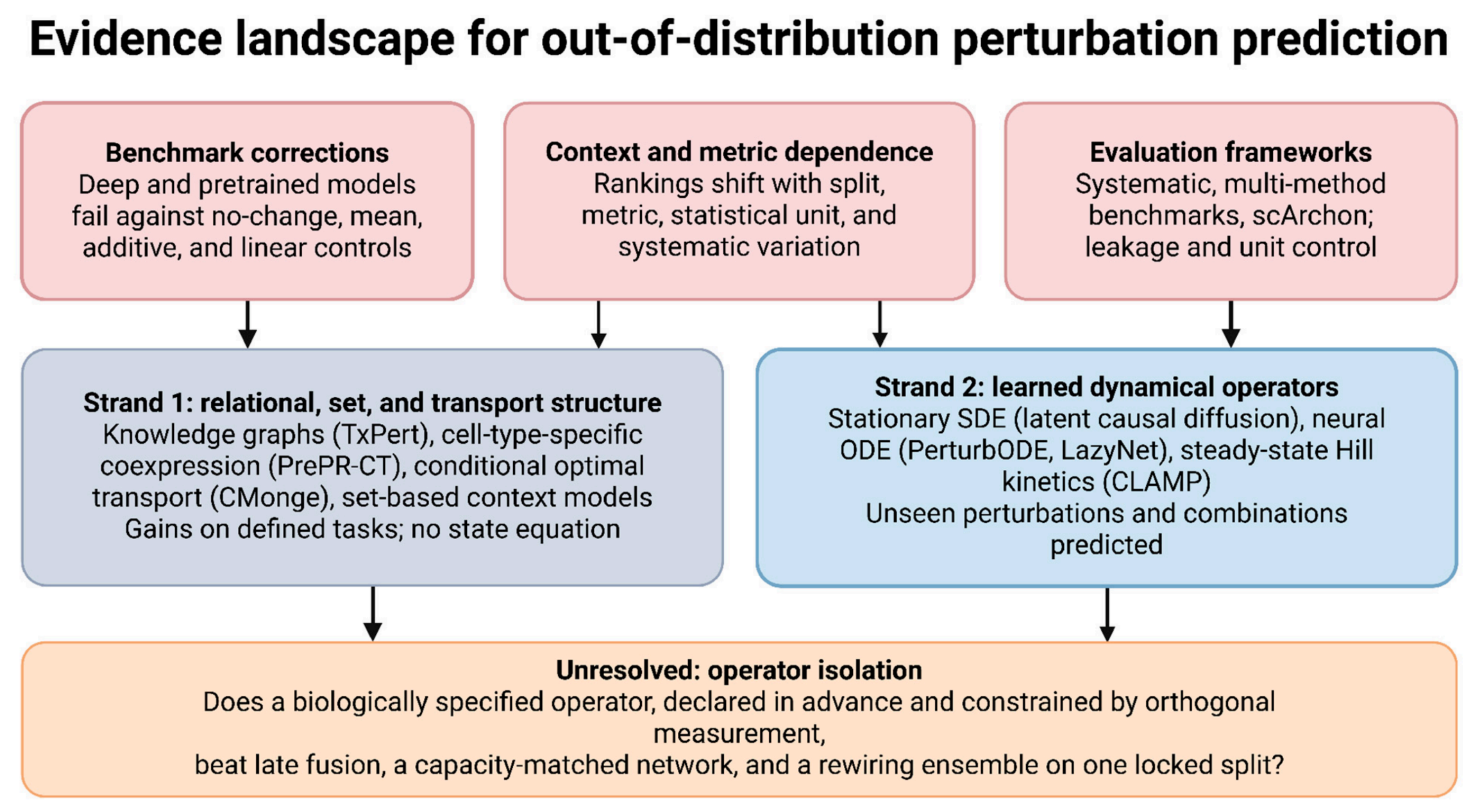
Evidence map for out-of-distribution perturbation prediction. Benchmark corrections have removed broad capacity-based claims. Subsequent gains have come along two strands, one relational, set-based, and transport-based, and the other dynamical, in which state equations are learned rather than curated. The incremental contribution of a biologically specified operator, isolated against matched controls, remains the unresolved comparison. Created in BioRender. Niazi, S. (2026) https://BioRender.com/afg5k7j (accessed on 15 September 2026).

Table 4 groups the evidence into five categories rather than a binary positive-versus-negative account. The first shows that deep or pretrained models may fail against strong controls. The second shows that measurement and evaluation choices can create or remove apparent gains. The third shows that relational, set, and distributional structure can improve defined tasks. The fourth shows that learned state equations now predict unseen perturbations and combinations and recover interpretable network structure. The fifth is the unresolved comparison addressed here. No identified study isolates the incremental value of a biologically specified operator against late fusion, a capacity-matched static nonmechanistic model, a capacity-matched learned-dynamical operator, and topology-rewired controls under one locked out-of-distribution evaluation, which is a narrower and more defensible claim than any assertion that dynamical formulations are absent from the field [10,13,54,55,56,57,58] (Figure 2).

## 4. Mechanistic Operator Families and the Observations They Require

### 4.1. Kinetic and Stochastic Descriptions

A kinetic model expresses the change in a concentration or activity vector as dx/dt = S v(x, theta, I), where S is a stoichiometric or incidence matrix, v collects rate laws, theta denotes parameters, and I denotes the intervention. Under standard smoothness and uniqueness conditions, the solution map is differentiable with respect to parameters and initial conditions. That qualification carries weight. Events, discontinuous interventions, bifurcations, non-unique solutions, and stiffness can make sensitivities unstable or numerically wrong even when a formal derivative exists. Continuous adjoints, discrete adjoints, direct sensitivities, checkpointing, and finite-difference verification are different numerical methods rather than interchangeable implementations [21,64,65].

Large signaling models show that kinetic operators can be assembled at scale. The pan-cancer pathway model and its scalable open-source implementation integrate receptor signaling, proliferation, and apoptosis informed by single-cell variability. Their lesson for hybrid design is not that a large operator should be dropped into a foundation model, but that parameter estimation, calibration data, observation mapping, and model reduction are themselves scientific work. A smaller operator is preferable when its variables are observable and its falsification boundary is defined [14,66,67].

Low copy number, transcriptional bursting, and cell-to-cell variability may require a discrete stochastic description. Exact stochastic simulation samples trajectories from the chemical master equation without resolving the full state space, tau-leaping groups reaction events for efficiency, and finite-state projection truncates the state space with controlled error for moderate systems. A deterministic approximation is not automatically invalid at low copy number, because suitability depends on the target, timescale separation, propensities, and whether the measured endpoint averages over many cells [68,69,70,71,72]. Discrete-event trajectories generally lack pathwise differentiability in the reaction rates, but likelihood-ratio estimators, reparameterizable approximations, coupled finite differences, smoothed relaxations, and learned surrogates all produce gradients with distinct bias, variance, and cost. A manuscript should name the estimator and validate it on a system with known sensitivities [73,74].

### 4.2. Constraint-Based, Rule-Based, and Spatial Descriptions

Flux balance analysis defines a feasible polytope through steady-state mass balance and flux bounds and then selects a point using an objective or secondary criterion. Dynamic flux balance analysis couples repeated optimization to extracellular or regulatory dynamics, and metabolism-and-expression models add resource allocation. These methods provide genome-scale coverage without estimating thousands of kinetic constants, but the objective, the bounds, and the steady-state assumption are parts of the mechanism and must be justified in context [75,76,77,78]. Feasibility defects, bound violations, or optimality conditions can be penalized, yet a penalty does not reproduce the selected optimum, guarantee the intended objective, or resolve degeneracy. Exact embedding requires a differentiable optimization layer or implicit differentiation through optimality conditions, and the resulting solution map is piecewise differentiable and nonsmooth where the active set changes [79,80].

Rule-based models describe interactions at the level of binding sites, phosphorylation sites, and local molecular patterns, which avoids enumerating every species before simulation. BioNetGen and PySB provide such representations for combinatorial signaling, and logical and Boolean models are useful when topology is known but kinetic data are sparse, with continuous-time stochastic logic extending the discrete formalism [81,82,83,84,85]. A rule-based system can be compiled to a reaction network and differentiated after reduction, whereas a Boolean transition usually requires relaxation or a probabilistic estimator; the relaxed and discrete systems must then be compared at the endpoint of interest.

Reaction–diffusion models bring geometry, transport, and boundary conditions into intracellular chemistry, and agent-based models represent cells as discrete entities with rules for division, death, migration, mechanics, and contact [1,86,87,88]. Their cost is set by grid resolution, geometry, stiffness, event frequency, and tolerance rather than by trainable parameter count. Neural operators can approximate parameter-to-solution maps and reduce repeated solving, but their reliability depends on training-distribution coverage, conservation tests, surrogate-error estimation, and evaluation beyond the sampled range, so they accelerate a validated solver rather than replace one [89,90].

### 4.3. Interfaces and the Limits Set by Measurement

A sequence of differentiable modules is not a multiscale biological model. Each interface must declare the variables passed upward and downward, their units, aggregation and disaggregation rules, the time resolution of each module, initial and boundary conditions, and conserved quantities. When modules run on different timescales, a synchronization or operator-splitting scheme is required and its splitting error must be quantified, because feedback can destabilize a coupled system whose components are individually stable. Model uncertainty and numerical error must cross interfaces rather than being computed separately and discarded [14,18,19,20]. This requirement is the reason the proposed minimum system contains one operator. Adding molecular, organelle, cell, and tissue modules at once makes diagnosis impossible, because a wrong prediction may come from the operator, a nonidentifiable parameter, a faulty interface, a synchronization error, an observation map, or an inaccurate gradient.

No single-cell assay measures the full biochemical state. Transcript counts constrain transcriptional outputs but not phosphorylation, receptor occupancy, protein degradation, metabolite flux, or organelle morphology. Proteomic and phosphoproteomic measurements identify pathway states more directly, isotope tracing constrains metabolic flux, imaging defines localization and morphology, and spatial assays constrain coupling among cells. A foundation-model embedding summarizes observations; it does not create missing state information. Table 5 maps operator families to gradient routes and to the observations required before inclusion in a hybrid model, and names the latent quantities that remain weakly identified when a modality is absent [13,37,40,41].

## 5. Patterns of Mechanistic and Machine-Learning Integration

### Six Patterns

Amortized inference trains a network to map observations to parameters or posteriors of a fixed simulator, which preserves the operator exactly and moves the learned component to inference. Simulation-based inference and its toolkits make this practical when likelihoods are intractable [91,92]. Universal and residual dynamics place a learned term inside the state equation, which increases flexibility and immediately raises the question of whether the residual has absorbed the biology that the interpretable terms were supposed to carry [93,94]. Soft residual penalties add equation defects to the loss, as in physics-informed networks. They are soft enforcement, weaker than the exact constraints imposed by a solver or by reparameterization, and the residual magnitude additionally serves as a diagnostic for approximate or learned components [95].

Differentiable simulators and optimization layers embed the operator in the computational graph and propagate gradients through solution or optimality conditions [64,79,80]. Constrained latent and causal representations restrict the geometry or dependence structure of a learned space without asserting biochemical dynamics [31,32,33,96]. Feature-level and late fusion pass simulator outputs to a downstream model without gradient flow, which is the weakest coupling and the most auditable [15,36].

**Figure 3:**
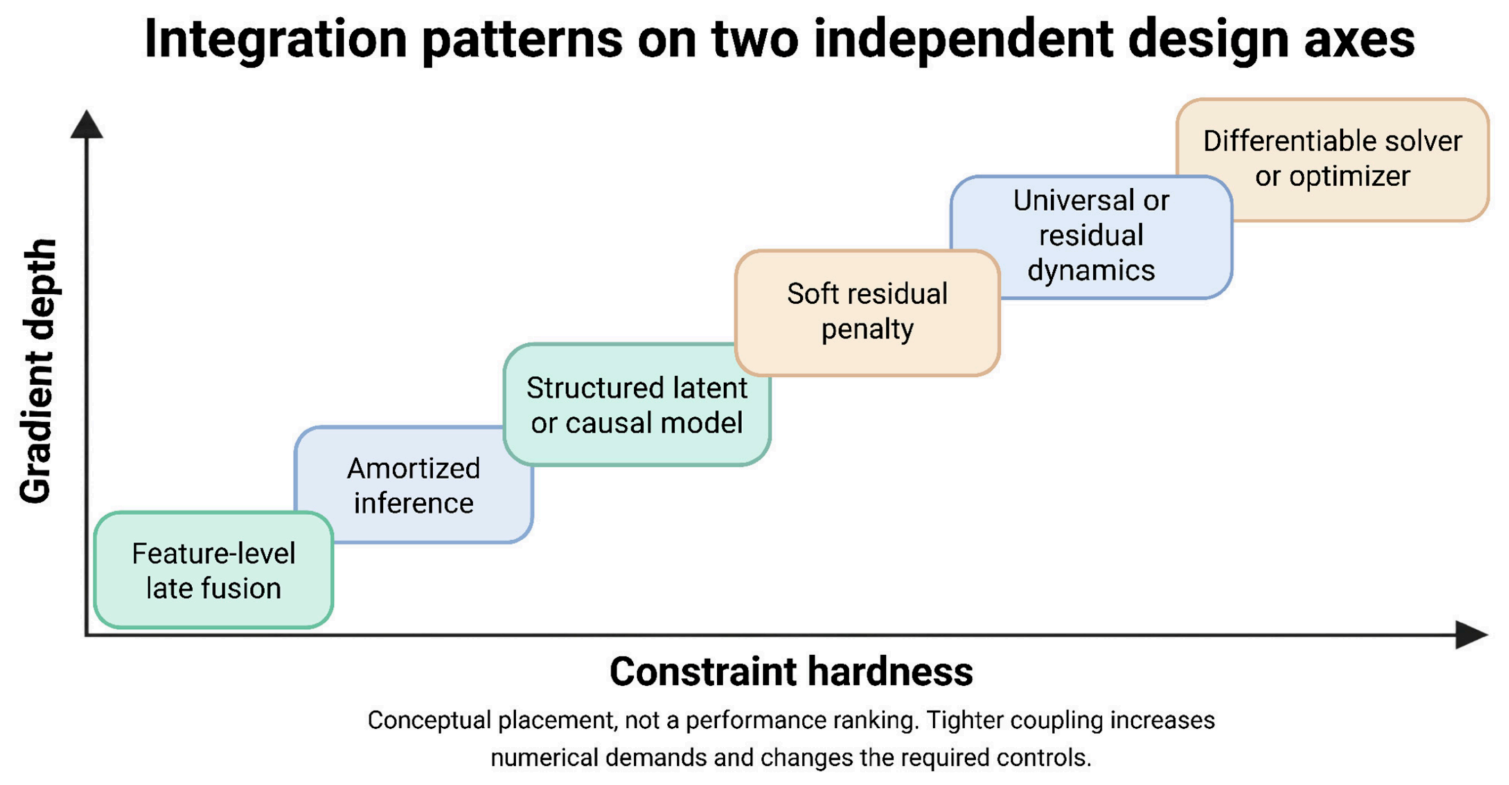
Six integration patterns positioned by constraint hardness and gradient depth. The arrangement is conceptual rather than a ranking. Tighter coupling enforces more structure while increasing exposure to identifiability problems, solver error, and optimization failure. Created in BioRender. Niazi, S. (2026) https://BioRender.com/xcthao5 (accessed on 15 September 2026).

Coupling strength and gradient depth are independent axes. A tightly constrained operator can be trained by derivative-free calibration, and a loosely constrained residual can be trained end to end. Tighter coupling enforces more structure while increasing exposure to identifiability failure, solver error, and optimization pathology. Table 6 states the deliverable of each pattern and the control needed before a positive outcome can be attributed to mechanism (Figure 3).

## 6. Requirements for a Credible Mechanistic Hybrid

### 6.1. Identifiability and Observability Precede Interpretation

Mechanistic structure does not resolve underdetermination. Structural identifiability asks whether perfect continuous observation would determine a parameter uniquely; practical identifiability asks whether the actual sampling schedule, noise, and replication suffice. Observability asks whether latent states can be reconstructed from measured outputs. Adding a neural initial-state model or parameter decoder introduces compensating directions and can make the combined system less identifiable than the operator alone. Any mechanistic claim must therefore report structural analysis where feasible, profile likelihood, posterior geometry, parameter correlations, state observability, and the effect of fixing or removing weakly identified quantities [22,23,24].

Predictive and mechanistic success must be reported separately. A model can forecast a transcriptomic response accurately while returning broad, multimodal, or biologically inadmissible parameters, in which case the prediction may still be useful but the kinetic labels must be withdrawn. A reduced operator may also recover interpretable pathway parameters without improving prediction at atlas scale, in which case it remains useful for calibration or design but is not evidence of superior virtual-cell prediction.

### 6.2. Initial Conditions and Intervention Semantics

A differential equation requires an initial condition, yet hybrid models often learn a parameter decoder and leave the initial state implicit. In destructive single-cell assays, the treated cell is never observed before treatment, so the initial state must be drawn from a matched baseline population, inferred through an observation model, or represented as a distribution. Uncertainty in the initial state strongly influences short-horizon predictions and must be propagated rather than replaced by a latent mean. Context should condition the initial state and the baseline parameters separately, because a cell line or donor can affect both [96,97].

The perturbation must enter through a biologically defined intervention operator rather than an unrestricted network that rewrites every kinetic parameter. A drug may change target occupancy, production or degradation rates, boundary inputs, or initial conditions; interference can reduce a target-specific synthesis rate; and a knockout can remove a reaction or a state. Allowing the intervention identifier to generate the full parameter vector lets the model encode responses in perturbation-specific kinetics and destroys the distinction between context and intervention. When an intervention affects more than one parameter, the affected set and its prior must be declared before the test partition is examined [29,37].

### 6.3. Observation Models, Replication, and Discrepancy

Single-cell RNA sequencing returns counts from distinct cells sampled after treatment rather than repeated observation of one cell, so the likelihood must describe a population or a replicate-level aggregate. A mean-response model can use pseudobulk counts or module scores for each independently treated well, donor, organoid, or cell line, while a distributional model can predict a conditional distribution or a transport map between baseline and treated populations. Without lineage or live-cell measurement, no such model can assert that a particular observed baseline cell followed a particular predicted trajectory [11,12,13]. Cells are informative within a replicate but do not increase the number of independent biological units; fifty thousand cells from one cell line do not demonstrate transport to a second cell line [48,49,50].

Every practical operator is incomplete relative to a stated context of use. If structural error is omitted, physical parameters absorb missing biology and appear interpretable while the model is wrong. Bayesian calibration separates parameter uncertainty from model discrepancy, but the two are not automatically identifiable: a flexible discrepancy can explain all observations without the mechanism, and an overly restrictive discrepancy forces the kinetic parameters to compensate. Discrepancy must therefore be localized in the state or observation space, constrained in rank or smoothness, given a declared prior, and included in sensitivity analysis [97,98]. Its magnitude is a warning indicator rather than a measurement of mechanistic contribution, because it also absorbs a wrong observation map, batch effects, wrong initial conditions, unmodeled intervention action, nonidentifiability, and solver error.

### 6.4. Gradient Provenance and Dimensionless Objectives

The phrase end-to-end differentiable is incomplete until the gradient path through each module is named. A kinetic system may use forward sensitivities, direct differentiation, a continuous adjoint, or a discrete adjoint. A stochastic simulator may use a likelihood-ratio estimator or a relaxation. A constrained program may use implicit differentiation. An event-driven agent model may have no usable pathwise gradient. Each choice has characteristic bias, variance, memory cost, and failure mode, so the implementation must report the method, solver version, tolerances, event handling, accepted and rejected steps, parameter-bound encounters, and finite-difference agreement for representative parameters [64,65,73,80]. Continuous adjoints are sometimes described as constant-memory solutions, yet reverse integration can be inaccurate in stiff, irreversible, or event-driven systems, and gradient clipping stabilizes optimization without correcting wrong sensitivities.

A hybrid objective combines a count likelihood, an initial-state term, a parameter penalty, a discrepancy penalty, and any numerical defect. These terms are not comparable unless they are dimensionless. Counts enter through a declared likelihood, equation defects are normalized by species-specific and timescale-specific quantities, prior deviations enter as standardized quadratic or logarithmic terms, and discrepancy is normalized to an observational scale. Equation (1) is a schematic of the Stage 2 objective and not the full joint posterior, which additionally contains the direct pathway-readout likelihoods used at Stage 1, the priors on the intervention parameters, the context model where it is active, and the discrepancy hyperpriors. The complete joint specification is given in Supplementary File S1.

(1) ℒ = −E_q_[log p(y | x(x_0_,θ,θ_I_), δ)] + KL[q_η(x_0_ | y_0_,c) ‖ p(x_0_ | c)] + λ_θ (θ − μ)^T^ Σ^−1^ (θ − μ) + λ_I_ (θ_I_ − μ_I_)^T^ Σ_I_^−1^ (θ_I_ − μ_I_) + λ_δ ‖Rδ‖^2^ + λ_num_ ℒ_num_

Equation (1) is a variational objective and its terms are fixed as follows. The initial state is not penalized at a point estimate. It is given an amortized approximate posterior q_η(x0 given y0 and c) and a conditional prior p(x0 given c) built from the measured baseline distribution, and the first term is the expected conditional log-likelihood under samples drawn from q_η while the second is the Kullback–Leibler divergence between the two, so that initial-state uncertainty is propagated through the solver rather than collapsed. The vectors μ and μI and the matrices Σ and ΣI are prior means and covariances for the kinetic and intervention parameters on the log scale, fixed from independent measurements before fitting. R is the regularization operator on the discrepancy coefficients defined in Section 7.5. The weights λ_θ, λI, and λ_δ are tuned on validation data within a declared grid and reported with sensitivity, while λnum is fixed at the value that equates the numerical term to solver tolerance. The numerical term contains only defects that are not already enforced by construction, because if the solver integrates, the system to the declared tolerance, an additional residual duplicates the same constraint and distorts the fit [21,95].

### 6.5. Uncertainty, Leakage, and Compute

**Figure 4:**
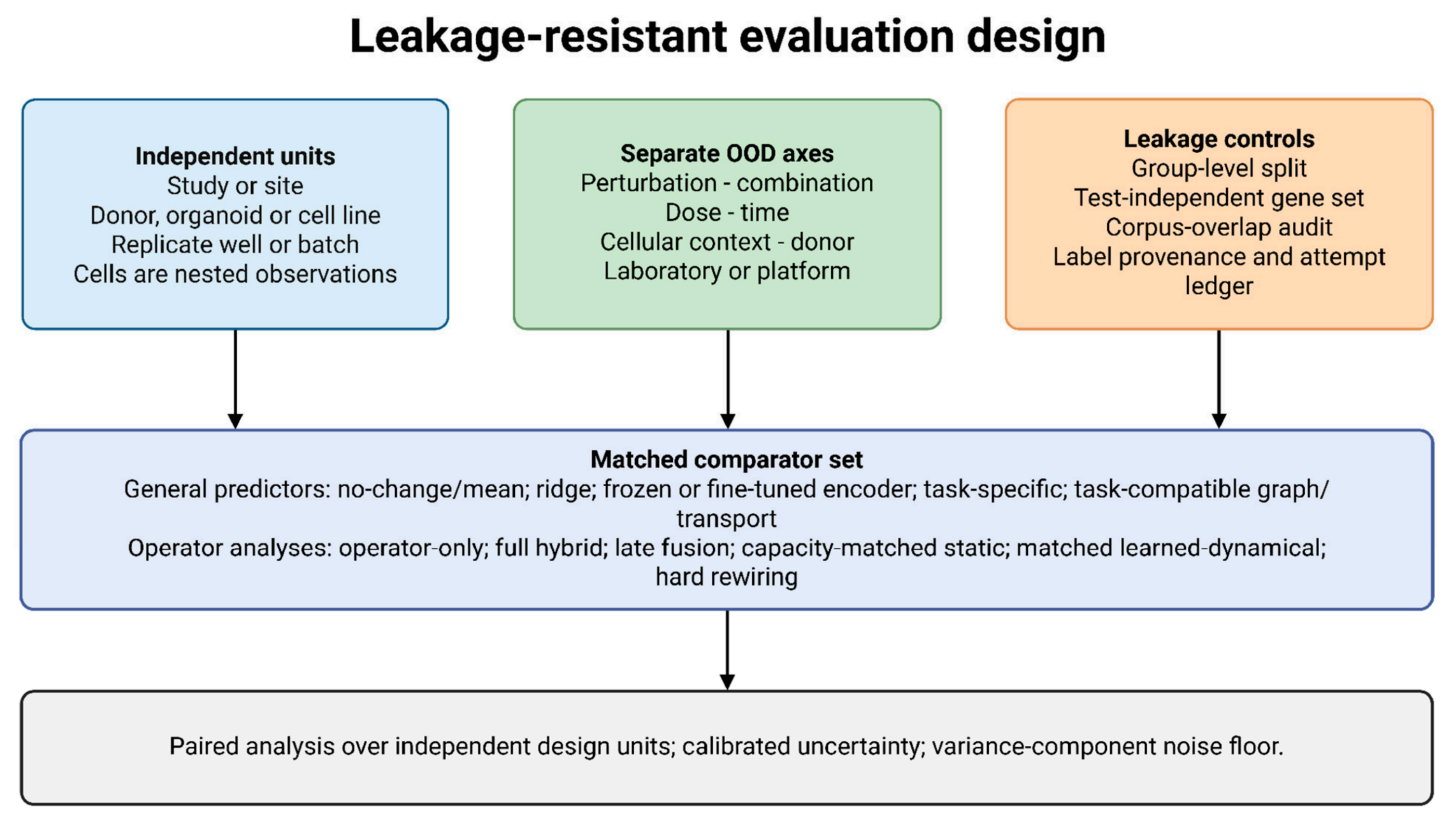
Leakage-resistant evaluation design. Cells are nested observations, generalization axes are scored separately, and leakage controls apply to both labeled data and pretraining corpora. The matched comparator set includes task-compatible general predictors and four operator-isolation controls: late fusion, a capacity-matched static model, a matched learned-dynamical operator, and validation-frozen hard rewirings. Primary inference is paired over independent design units and uses calibrated uncertainty and the variance-components noise floor of Equation (2). Created in BioRender. Niazi, S. (2026) https://BioRender.com/a313xdp (accessed on 15 September 2026).

A useful uncertainty analysis names its sources rather than reporting a generic interval. Count noise, replicate sampling, initial-state uncertainty, kinetic-parameter uncertainty, structural discrepancy, uncertain intervention action, encoder uncertainty, solver error, missing modality, and distribution shift are distinct. A negative-binomial likelihood, a posterior over parameters, a low-rank discrepancy, an ensemble over the learned component, and a solver-tolerance study each represent different sources, and they must be propagated jointly to the endpoint and assessed through coverage, width, proper scoring rules, reliability diagrams, and subgroup analysis [99,100,101]. Deep ensembles and conformal methods give predictive uncertainty under stated assumptions, and conformal coverage rests on exchangeability or on the assumptions of a particular shift-adapted extension. Neither identifies structural model uncertainty, and the operative difficulty in this setting is distribution shift across contexts rather than the fact that an alternative mechanism is unmodeled. A decision-facing hybrid must therefore abstain when the posterior is broad, the discrepancy is large, the context lies outside the calibration domain, or posterior predictive checks fail, with each of those conditions given a numerical threshold in Section 8.3 (Figure 4).

Groups must be partitioned at the level that defines novelty, gene sets and preprocessing must be fixed without test outcomes, and every pretrained encoder must undergo a corpus-overlap audit. The test set stays locked until architecture, priors, ablations, metric orientation, and hyperparameter budget are frozen, and every test evaluation is recorded in an attempt ledger, because repeated inspection converts a nominally held-out set into a tuning resource [7,8,51]. Compute is matched on a single declared resource budget rather than on trial counts, because equal numbers of trials, seeds, and early-stopping opportunities are not equal compute when one model repeatedly solves a stiff system and another performs inexpensive matrix operations. The primary budget is total accelerator-hour-equivalents, with central-processor time converted at a fixed declared rate and with trials, seeds, and stopping decisions free within that budget. Energy and wall-clock time are reported alongside, and the primary results include an accuracy-versus-compute curve rather than a single best score [65,102].

### 6.6. Mechanistic Predictive Gain

Mechanistic predictive gain is proposed as a reporting quantity for the incremental predictive value of the biologically specified structure. The name is deliberate: the quantity is a difference between normalized predictive scores and is not an information-theoretic measure, so it has no relation to Shannon information, mutual information, Kullback–Leibler divergence, or information gain. Let S(M) be a prespecified score oriented so that larger values are better and mapped affinely onto the interval between a trivial baseline and a reliability-adjusted noise floor. For the primary root-mean-square-error endpoint, the transformation is given in Equation (2), where RMSEbase is the error of the no-change or training-context-mean predictor and RMSEfloor is the irreducible prediction error defined from the same variance-components model used for inference. The same affine map is applied to each secondary metric using that metric’s own baseline and ceiling or floor, with orientation stated explicitly. On this scale, S(M) = 1 means prediction as accurate as the frozen measurement and replication design permits under the registered noise-floor model; it does not mean that all biological signal or causal structure has been recovered. Values above 1 and below 0 are permitted and are never truncated, because truncation would bias paired differences.

(2) S(M) = [RMSEbase − RMSE(M)]/[RMSEbase − RMSEfloor],     RMSEfloor^2^ = [σuc^2^ + σw^2^(1 + 1/m)]/n_cult

The noise floor is derived at the same aggregation level as the primary score. The standardized treatment-minus-control effect is first computed within each culture and then averaged across C independent cultures for each held-out intervention. Because treated wells are compared with control wells from the same culture and time, a shared additive culture-by-time effect cancels rather than entering the floor. In Equation (2), σuc^2^ is the intervention-by-culture random-effect variance from the prespecified training mixed model after the fixed design covariates, σw^2^ is residual well-level variance after standardization, m is the number of matched control wells entering the within-culture control mean, and n_cult is the number of independent cultures averaged in the intervention-level endpoint. For the eight-component endpoint, the implementation uses the corresponding covariance matrix before culture averaging. These components are estimated from Stage 1b and training data only and do not depend on which candidate predictor is being evaluated. A predictor that explains variation treated as random by this design model may therefore achieve S(M) greater than one; such values are permitted and reported. Because RMSEfloor changes with n_cult, the normalized scale and the 0.25 MPG threshold are design-specific; n_cult, the normalization rule, and the decision threshold are therefore frozen together before Stage 2 test access. If the estimated gap between RMSEbase and RMSEfloor is smaller than twice its own standard error, the endpoint is declared insufficiently informative, and the Stage 2 design is revised before the test partition is generated.

Two analyses are defined, and they must not be conflated. The first is predictive superiority: the hybrid against the single strongest task-compatible general predictor, whose identity is selected on validation data and frozen before the test set is opened. The second is mechanistic attribution, which is what mechanistic predictive gain measures. Its comparator is the validation-selected strongest member of four operator-isolation controls: late fusion, a validation-frozen hard-rewiring ensemble, a capacity-matched unstructured network, and a capacity-matched learned-dynamical operator. Equation (3) defines the gain against that fixed isolation comparator, so no maximum is taken over noisy test scores. The four isolation controls are then compared simultaneously with the hybrid using a multiple-comparisons-with-the-best procedure that controls the family-wise error rate. A mechanistic claim requires the gain to clear its threshold against the fixed comparator and requires the simultaneous interval to exclude the negligible region for all four controls; a predictive claim requires only the first analysis.

(3) MPG = S(M_bio_) − S(M_iso*_),   M_iso*_ fixed on validation data before the test set is opened

The rewiring control is an ensemble rather than one arbitrary graph, and its hard-negative subset is frozen on validation data before test access by the procedure specified in Section 8.3, so neither a maximum nor a percentile is selected after seeing test outcomes. Confidence intervals come from paired resampling of the independent design units, and MPG is reported alongside parameter admissibility, discrepancy share, calibration, and compute. A positive predictive gain with an MPG that is negative, negligible, or statistically indistinguishable from zero supports the predictor but not the claim that the biologically specified coupling supplied the gain.

## 7. A Minimum Testable Hybrid for the Unfolded Protein Response: A Prospective Specification

Section 7 and Section 8 are a prospective specification. None of the stages described below has been executed: no cells have been cultured, no model has been fitted, no synthetic-recovery study has been run, and no number that follows is a result. The equations, parameter counts, sample sizes, and thresholds are design choices that an implementing group would deposit in a public dated protocol before the corresponding data exist, and where the text uses the present tense, it states what the protocol requires rather than what has been observed. Section 10 sets out what remains unknown about the feasibility of the design until Stages 0 and 1 have been run.

### 7.1. Why This System

The unfolded protein response is broad enough to be interesting and narrow enough to test. Endoplasmic-reticulum stress is sensed through PERK, IRE1, and ATF6, which together control translation, transcription, chaperone capacity, degradation, and cell-fate decisions. The pathway offers direct molecular readouts, well-characterized stressors, established genetic reagents, and a long history of dynamical modeling [103,104,105,106,107]. The Adamson Perturb-seq experiment connects the pathway to single-cell transcriptomic perturbation data in K562 cells, and mechanistic studies define the branch interactions and feedback that any reduced model must contain [37].

Five properties make the pathway suitable for testing a biologically specified operator in particular, as distinct from testing perturbation prediction in general. First, its topology can be declared rather than inferred: three sensor branches with known activation logic, known transcriptional outputs, and an experimentally established negative-feedback loop define a signed directed graph that can be written down before fitting, which is the precondition for the rewiring and wrong-pathway controls of Section 8 to be meaningful. Second, the interventions have known molecular actions on named terms: tunicamycin raises unfolded-protein load by inhibiting N-linked glycosylation, thapsigargin lowers folding capacity by inhibiting the sarco-endoplasmic-reticulum calcium ATPase, and knockdown of a sensor or effector reduces a specific attainable pool or production term, so intervention semantics can be fixed prospectively instead of learned from the response. Third, the latent states of the operator are directly measurable with routine assays: phospho-eIF2α, ATF4, spliced XBP1, cleaved ATF6, BiP, CHOP, and GADD34 can each be quantified by protein, reporter, or targeted RNA measurement, which allows identifiability to be tested against orthogonal data rather than asserted. Fourth, the response is genuinely dynamical and nonmonotone: the eIF2α-ATF4-GADD34 feedback produces a pulse whose ascending and descending limbs separate adaptation from terminal stress, so a correct operator must predict something that a static map from intervention to endpoint does not represent, and the test is informative rather than trivial. Fifth, the pathway is small enough for a ten-state reduction, for enumeration of admissible rewirings, and for profile-likelihood analysis; its transcriptional outputs fall into recognized modules that allow the endpoint to be restricted to predefined gene sets rather than the whole genome; and a canonical Perturb-seq dataset in K562 cells together with a long modeling literature supplies development data and parameter priors [37,103,104,105,106,107]. A pathway lacking any of these properties, for example one whose latent states cannot be measured or whose interventions act diffusely, would leave a failed test uninterpretable, because the failure could not be assigned to the operator rather than to the observation model or the intervention model.

The UPR is therefore a favorable case, and the choice does not claim that it represents all cellular processes. The conclusions available from the test are correspondingly asymmetric: a negative result under favorable conditions would weigh heavily against the incremental value of biologically specified structure on this class of task, whereas a positive result would establish feasibility in one well-characterized pathway and would not license extrapolation to pathways whose topology, intervention semantics, or latent states are less well constrained. A first test uses one cellular context and one out-of-distribution axis. The retrospective Adamson data support model development and stress testing, but an inferential claim requires independent biological replicates and a locked prospective batch, because cells drawn from one pooled experiment do not confirm transport to a new experiment [37,48,49].

### 7.2. Estimand, Endpoints, and Experimental Unit

The estimand is the replicate-level mean change in four prespecified unfolded-protein-response modules following a specified intervention in a single cellular context. The modules are PERK-eIF2α-ATF4 signaling, IRE1-XBP1s and endoplasmic-reticulum-associated degradation, ATF6 and chaperone induction, and CHOP-associated terminal stress. Module membership and equal within-module weights will be frozen from external pathway knowledge and training data before the Stage 2 test split is opened. The confirmatory panel will contain G = 120 eligible genes after globally excluding all 16 candidate CRISPR interference (CRISPRi) target transcripts and any additional transcript predeclared, before panel freezing, as directly guide-affected. Those excluded transcripts may still be used for target-engagement quality control and sensitivity analyses but cannot contribute to the primary module score. This is a design exclusion and is distinct from outcome-driven gene dropping, which is prohibited. The panel is replenished to 120 eligible genes before the split, and the endpoint weights are then frozen. The inferential unit for generalization is the intervention; wells are nested within interventions and independent cultures, and single-cell counts within a well sharpen the estimate of a well mean without adding independent units [37,44,50].

Two endpoint times are selected by a prespecified rule rather than fixed by the proposed model. Section 7.5 places inducible phosphatase feedback in the operator, which makes ATF4 a nonmonotone function of phosphorylated eIF2α and produces a pulse whose ascending and descending limbs carry different information, so a single time cannot separate adaptation from terminal stress. Stage 1 is designed to measure phospho-eIF2α, ATF4, and CHOP trajectories in the same cellular context at eight sampling times. The two Stage 2 times are then defined as the observed time of maximal ATF4 and the observed time of maximal CHOP-to-ATF4 ratio, rounded to the nearest sampled hour, and are frozen in the dated protocol before Stage 2 begins. Published K562 stress kinetics place these windows near 4 h and 12 h; those values are provisional planning assumptions only [103,104,105,106,107].

### 7.3. States and Parameter Budget

The operator is a reduced ordinary differential equation system rather than a whole-cell reconstruction. Its state vector holds unfolded-protein load, scaled active PERK abundance/activity, phosphorylated eIF2α activity, ATF4 activity, scaled active IRE1 abundance/activity, spliced XBP1 activity, nuclear cleaved ATF6 activity, effective chaperone capacity, CHOP-associated terminal stress, and inducible GADD34-directed phosphatase activity. These states cover the three sensor branches, adaptive feedback, and a terminal-stress output, and exclude metabolism, spatial transport, organelle geometry, and tissue interaction. Table 7 gives the biological interpretation, preferred direct observation, and role of each state; every state whose preferred observation is a protein, phosphoprotein, or reporter quantity is latent at Stage 2 and is constrained through Stage 1 rather than inferred from RNA alone [103,104,105,106,107].

The capacity ledger is stated explicitly because the mechanistic operator is the smallest trainable component of the model and a comparison that matched only its parameters would isolate little. Of the 6365 parameters trainable at Stage 2, at most sixteen are biologically specified kinetic or chemical-intervention quantities, and the sixteen genetic action inputs are fixed from independent prechallenge evidence rather than fitted (Section 7.4), so Table 8 separates trainable capacity from fixed biological specification. That asymmetry is why the capacity-matched controls in Section 8.1 are defined on the complete trainable system and why discrepancy ablation is confirmatory rather than supplementary.

### 7.4. Initial State, Context, and Intervention Operator

Observation count alone does not establish identifiability, and the constraint here is structural and practical rather than a ratio. Ten states are observed only through a restricted transcriptomic map at two times, several latent activities are never measured directly at Stage 2, the four module outputs are correlated, and parameter compensation between synthesis and degradation terms and between the operator and discrepancy is expected by construction. A kinetic parameter or identifiable combination remains free at Stage 2 only if its Stage 1 profile-likelihood 95 percent interval spans less than one natural-log unit, its posterior places less than 5 percent of its mass within 1 percent of either bound, and its absolute pairwise posterior correlation with every other free quantity is below 0.9. Parameters failing any criterion are fixed to independently measured values, constrained more tightly, or pooled into identifiable combinations. The prospective protocol caps the number of free kinetic quantities at twelve and prespecifies sensitivity analyses at caps of eight and sixteen. No pilot or synthetic result is being reported here. If Stage 0 shows that twelve free quantities cannot be recovered at the planned sampling density, the cap is lowered, and the amended value is publicly time-stamped before Stage 2 test labels exist [22,23,24].

The initial state is not assumed known from an unperturbed transcriptome. For biological replicate r in context c, a restricted initial-state model returns an approximate posterior distribution rather than a single vector, and direct baseline pathway measurements enter that distribution when available. The context-parameter model in Equation (4) is separate and hierarchical, but it is inactive in a single-context experiment. With one cellular context, the loading matrix B, context random effect bc, and gene-specific context loadings cannot be separated from the population parameters and gene intercepts, so all three are removed at Stages 1 and 2 and the equation reduces to its population term. They are estimated only at Stage 3b, which requires at least four independent training contexts [22,23,24,96].

(4) x0,r ~ qη(x0 | y0,r, c),     log θc = log θpop + Bzc + bc     (context terms inactive at Stages 1 and 2)

The intervention is never allowed to rebuild the kinetic vector. Tunicamycin enters as a dose-dependent increase in protein-folding load through inhibition of N-linked glycosylation, thapsigargin as a time-gated reduction in calcium-dependent folding capacity through inhibition of the sarco-endoplasmic-reticulum calcium ATPase, and CRISPR interference through a target-specific availability or process multiplier applied only to a declared term. The chemical potency parameters are collected in θI and counted in Table 8. Genetic intervention strength is defined from an independently measured guide efficiency and an independently fixed target-to-process action coefficient rather than from the locked transcriptomic response. What is prohibited is not uncertainty in intervention strength but action outside the declared terms [37,104,106].

CRISPR interference is not an instantaneous event at time zero and is not modeled as one. Guides are introduced and selected five days before the stress challenge, so target reduction is established before stressor addition. The baseline state is measured after knockdown and before challenge, allowing prechallenge equilibration to alter the initial-state distribution. For each guide j, knockdown efficiency e_j is measured by targeted RNA assay and, for direct protein-state targets where feasible, checked by an orthogonal protein or activity assay. The target-to-process coefficient ρ_j is characterized only from the independent proximal or functional assay appropriate to the declared action. Rather than treating either quantity as known exactly, the protocol freezes their assay-derived probability distributions before Stage 2 and propagates the resulting uncertainty in a_j = 1 − ρ_j e_j through the primary predictive distribution. Neither distribution is updated with Stage 2 outcomes. A candidate target is ineligible for confirmatory testing if the independent assay cannot identify its action sufficiently to satisfy the target-engagement precision criterion frozen before the Stage 2 partition is generated.

A held-out intervention is defined so that no intervention-specific quantity is obtained from the locked response. The primary genetic held-out unit is a target with its guide. Its assay-derived e_j and ρ_j distributions may use prechallenge target-engagement or functional data, but neither may use post-challenge transcriptomic outcomes. The secondary chemical dose analysis holds out an interior dose of a compound whose remaining doses are in training; the dose-response parameters are therefore learned without the held-out response and no new parameter is introduced at test time. Any intervention whose mathematical action can be specified only after fitting its own locked response is ineligible for confirmation and remains in training or exploratory analysis.

### 7.5. Reduced Operator and Observation Model

The operator uses saturating activation and first-order turnover, with signs fixed by pathway knowledge and magnitudes estimated within independent bounds. The equations are a protocol specification and do not assert uniqueness of the unfolded-protein-response model. Sensor activation is governed by the ratio of unfolded load to effective folding capacity, adaptive outputs raise H, which feeds back on U and the sensors, and CHOP accumulates under sustained ATF4, ATF6, and residual stress. The PERK, IRE1, ATF6, and phospho-eIF2α variables are scaled active abundances or activities rather than fractions of an assumed immutable total pool, so target knockdown changes the attainable active pool rather than being misrepresented as a change in the intrinsic activation rate. Each state is constrained to be non-negative through transformation or a positivity-preserving solver [103,104,105,106,107].

The sensor, chemical, and genetic intervention functions are defined in Equation (5). Each sensor activation is a Hill function of the ratio of unfolded load to effective folding capacity with a fixed exponent of two and a branch-specific threshold fitted within bounds. Tunicamycin acts only through the load term as a saturating function of dose. Thapsigargin acts only through a folding-capacity multiplier that equals one before addition and is bounded below at 0.05 after addition. For a genetic target j, the mapped availability or process multiplier is a_j = 1 − ρ_j e_j, with all untargeted multipliers equal to one and with uncertainty in e_j and ρ_j propagated from the frozen external assays. Table 9 gives the single allowed primary mapping for every target. A common convention is used for production-type actions: when a_j represents reduced availability of a targeted species, it scales that species’ complete production or input block unless a different biological action is declared prospectively. Thus, a_A, a_X, a_C, a_G, and a_H scale their complete production or input blocks, whereas a_P, a_I, a_E, and a_F remain attainable-pool ceilings. For HSPA5 and other chaperone targets, the primary a_H action therefore scales the whole effective-chaperone production block in Equation (9). Two prespecified sensitivity models test a basal-only a_H k_H0 action and an alternative a_H multiplier on k_fold in Equation (6). No intervention may alter an additional term after test outcomes are seen.

(5) hj(U,H) = (U/H)^2^/[Kj^2^ + (U/H)^2^], j ∈ {P,I,F};   qload(u,d,t) = ETM d/(EC50TM + d)·1[t ≥ 0];   gCa(u,d,t) = 1 for t < 0 and max{0.05, 1 − ETG d/(EC50TG + d)} for t ≥ 0;   aj = 1 − ρj ej

Knockdown strength therefore enters without outcome-based fitting, through the frozen e_j and ρ_j distributions and the assay-precision eligibility bound specified in Section 7.4, and the trainable intervention vector θI contains only the four chemical quantities counted in Table 8.

(6) dU/dt = qload(u,d,t; θI) + ksyn − kfold gCa(u,d,t; θI) H U/(KU + U) − aU kU U

(7) dP/dt = kP hP(U,H)(aP − P) − dP P;   dE/dt = kE P(aE − E) − (dE + kEG G)E;   dA/dt = aA kA E/(KA + E) − dA A

(8) dI/dt = kI hI(U,H)(aI − I) − dI I;   dX/dt = aX kX I − dX X;   dF/dt = kF hF(U,H)(aF − F) − dF F

(9) dH/dt = aH(kH0 + kHX X + kHF F + kHA A) − kHU H U/(KH + U) − dH H

(10) dC/dt = aC[kCA A + kCF F + kCU U/(KC + U)] − dC C;   dG/dt = aG(kG0 + kGA A + kGC C) − dG G

Two couplings require explicit representation. First, the three sensors are not interchangeable. PERK, IRE1, and ATF6 differ in activation threshold and persistence, with IRE1 and ATF6 outputs attenuating under sustained stress while PERK-driven translational control and CHOP induction may persist [104]. Each sensor therefore receives its own activation function and threshold, hP, hI, and hF with KP, KI, and KF. The availability multipliers aP, aI, and aF cap the attainable active state after knockdown rather than changing kP, kI, or kF, separating target abundance from activation kinetics. Second, the operator contains the CHOP-GADD34-eIF2α feedback present in data-entrained models of this pathway [105,106,107]. GADD34, induced by ATF4 and CHOP, recruits protein phosphatase 1 to dephosphorylate eIF2α; together with the constitutive activity absorbed into dE, this delayed negative feedback returns eIF2α toward baseline and restores translation. Omitting the loop would force other rates to mimic recovery and would remove the ability to distinguish adaptation from terminal stress, which is why the two endpoint times are selected as described in Section 7.2 [103].

The mechanistic state is linked to RNA through a restricted observation map, and the division between fixed and estimated quantities is stated exactly. The 120-gene confirmatory panel and equal within-module endpoint weights will be frozen before Stage 2 from eligible external and training information after removing the candidate CRISPRi target transcripts and any other predeclared directly guide-affected transcripts. What is estimated is a per-gene intercept, a single nonzero loading of that gene on its assigned module state, a per-gene dispersion, and, when the context block is active, a context loading. The loading vector in Equation (11) is sparse by construction, with one nonzero entry per gene and its sign constrained, and it is counted in Table 8. This prevents direct mechanical suppression of a targeted transcript from masquerading as pathway prediction. A prespecified sensitivity model may reinstate the excluded target transcripts using an explicit direct-knockdown observation term with externally regularized coefficients; any additional parameters are counted and that model is not used for the primary endpoint. Direct phosphoprotein and reporter measurements are modeled with assay-specific likelihoods; they constrain the latent pathway states and permit identifiability to be tested, which is not the same as guaranteeing that the states are inferable [37,96,104].

(11) y_rgt_ ~ NegBin(μ_rgt_, φ_g_),   log μ_rgt_ = log s_rt_ + α_g_ + w_g_^T^ x_rt_ + q_g_^T^ z_c_ + ℓ_g_^T^ δ_rt_

**Figure 5:**
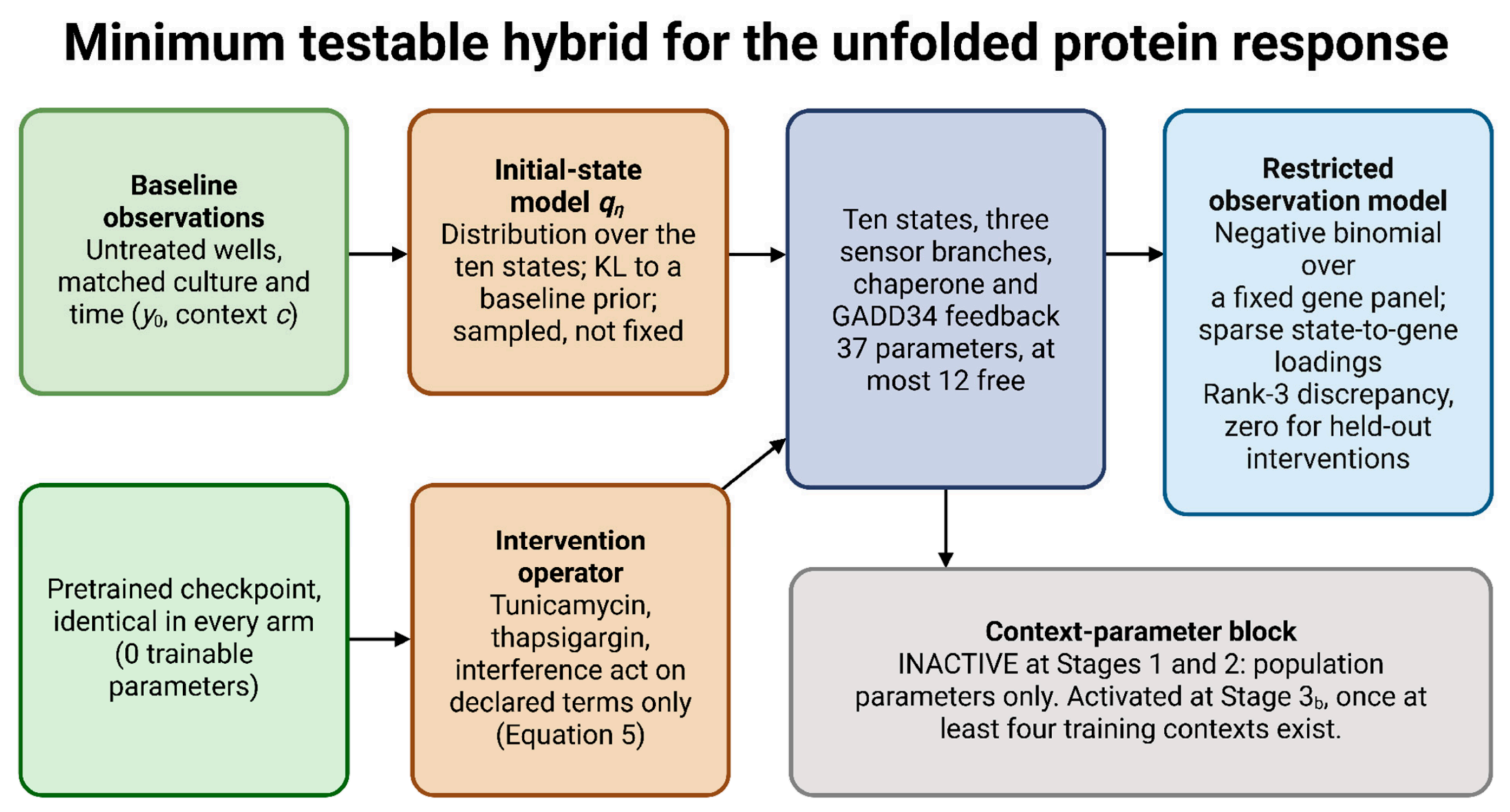
Minimum testable hybrid for the unfolded protein response. Baseline observations inform a distribution over initial states. The context-parameter block shown at the upper right is inactive at Stages 1 and 2, where population parameters alone are used, and is activated only at Stage 3b once at least four training contexts are available. Tunicamycin, thapsigargin, and interference act through the declared intervention operators of Equation (5) rather than rewriting the kinetic vector. One reduced ordinary differential equation module predicts pathway states, and a restricted-count observation model with a rank-three discrepancy produces a calibrated replicate-level distribution. Created in BioRender. Niazi, S. (2026) https://BioRender.com/wrtmyai (accessed on 15 September 2026).

The discrepancy is regularized but not forced to zero, and its factorization is stated explicitly because a flexible discrepancy can absorb the biology under test. In Equation (11), ℓg is a global loading vector of length three for gene g and δrt is a latent vector of length three with a Gaussian prior whose diagonal scales and time offsets are trainable; the resulting 369 discrepancy parameters are itemized in Table 8 and the full factorization is given in Supplementary File S1. The per-well latent δrt values are integrated out in training and prediction and are not counted as trainable parameters. For a held-out intervention, the predictive distribution integrates δrt from the learned prior without conditioning on the held-out outcome; the point prediction uses the resulting posterior predictive mean, not a latent value fitted to that response. With only two transcriptomic times, R is a first-difference penalty between the two time-specific offsets plus ridge regularization on discrepancy loadings and scales. Rank three is fixed prospectively; cross-validated fits at ranks 0, 1, 2, and 5 are sensitivity analyses rather than a rank-selection procedure. Mechanistic interpretation is withdrawn when discrepancy accounts for more than 30 percent of predicted module-effect variance, when prespecified posterior predictive checks fail, or when kinetic posteriors shift by more than one posterior standard deviation under the alternative discrepancy prior [97,98] (Figure 5).

### 7.6. Stage Gates and Data Provenance

**Figure 6:**
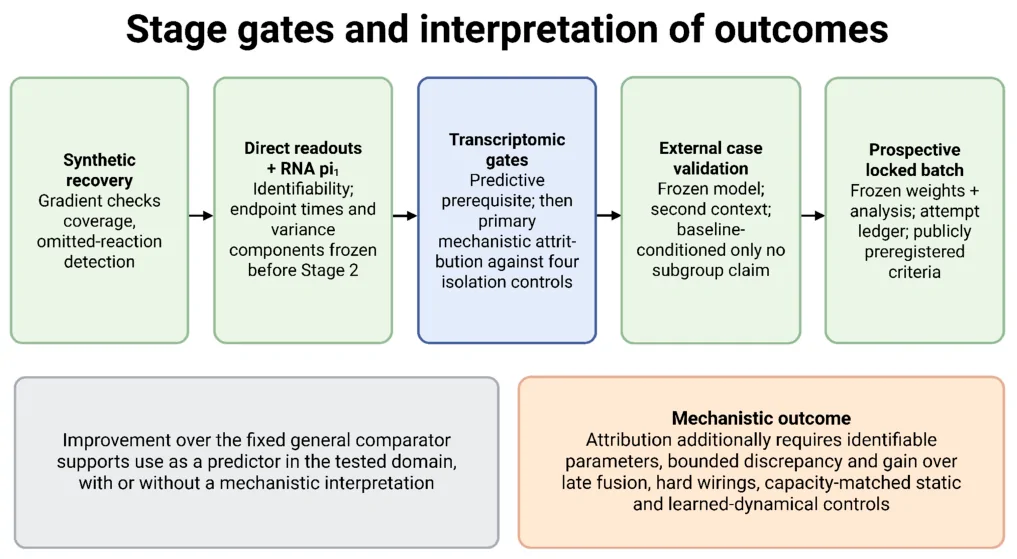
Stage gates and interpretation of outcomes. The model advances from synthetic recovery to direct pathway measurement plus a training-only transcriptomic variance pilot, restricted transcriptomic prediction with a prerequisite predictive gate and a primary mechanistic-attribution gate, second-context external case validation, and a locked prospective batch. Predictive performance and mechanistic attribution are assessed separately, so a useful predictor can be retained even when kinetic interpretation fails. Created in BioRender. Niazi, S. (2026) https://BioRender.com/7zivddz (accessed on 15 September 2026).

Development proceeds through five stages, shown in Figure 6 and summarized in Table 10. Stage 0 is synthetic recovery with known dynamics, realistic count noise, batch effects, and one deliberately omitted reaction. Stage 1 combines direct pathway measurement with a training-only transcriptomic variance pilot, determines which kinetic quantities remain identifiable, and locks the two endpoint times. Stage 2 adds the restricted transcriptomic observation map and separates a predictive gate from the primary confirmatory mechanistic-attribution gate. Stage 3 applies the frozen model to one additional cellular context as baseline-conditioned external case validation. Stage 4 applies the frozen system to a prospectively generated batch with code, thresholds, and analysis rules fixed before outcomes are released. Failure at any stage directs work to the failing component rather than to architectural expansion [15,16,24,37]. The staging also orders the work by cost: Stage 0 and a retrospective development run on the Adamson data require no new experiments, Stage 1 is a targeted time series of a few pathway readouts, and only Stage 2 onward requires new perturbation sequencing, so an implementing group can stop, and report, at the first stage that fails.

The data required at each stage must be named because the retrospective resources do not supply them. The Adamson screen is an interference experiment in K562 cells with transcriptomic readout and no branch-resolved protein measurements, and the chemical atlases do not provide the complete phospho-eIF2α, ATF4, XBP1s, cleaved-ATF6, BiP, CHOP, and GADD34 time series required here [37,40,42]. Stage 1 therefore requires a new K562 dose-and-time series using only interventions assigned to the Stage 2 training set, with those direct or targeted pathway readouts at eight sampling times. After the two endpoint times are selected, Stage 1b generates a transcriptomic variance pilot at those times using training interventions and matched controls in separate pilot cultures and passages that are not reused as the six Stage 2 confirmatory cultures. Stage 1b data are used for module-time covariance, variance-components, normalization, and power estimation and are not pooled into the locked Stage 2 confirmatory outcome set. Before Stage 3, a separate baseline reference panel of at least twenty untreated cultures, generated or assembled under the same library chemistry and preprocessing and drawn from prespecified eligible contexts, is frozen solely to calibrate the context-distance threshold; it contains no intervention outcomes and is not used to fit the predictor. If such a reference panel is unavailable, the Stage 3 distance-based abstention claim is deferred rather than estimated from six cultures. Stage 4 requires an independently generated batch after model freezing.

The Stage 2 intervention design is prospective and is frozen only after Stage 1 and Stage 1b. The genetic axis contains 16 candidate interference targets partitioned into 8 training, 3 validation, and 5 held-out test targets. The held-out set is action-diverse: HSP90B1 (a_H), SEL1L (a_U), and MBTPS2 (a_F) test new genes through action classes already represented in training and form the primary confirmatory known-action stratum; XBP1 (a_X) and PPP1R15A/GADD34 (a_G) test action classes not exercised in training and form a prespecified secondary new-action stratum. The equal-weighted five-action summary is reported only as a secondary integrated stress test and cannot by itself support attribution to either target generalization within a known action class or extrapolation to a new action class. Validation uses EIF2S1 (a_E), PDIA4 (a_H), and MBTPS1 (a_F), spanning three distinct action classes; DNAJB11 is assigned to training. Because three validation actions still provide a small selection set, comparator ranking and hard-rewiring selection stability are reported under action-stratified resampling rather than treated as noise-free. The chemical axis contains 12 stressor-dose conditions, tunicamycin and thapsigargin at six doses each, partitioned into 8 training, 2 validation, and 2 held-out interior doses for a separate dose-generalization analysis. Six independent Stage 2 cultures are to be prepared from separate passages; within each culture, one well is treated per intervention per endpoint time, giving 336 perturbed wells. Controls are two vehicle wells and two nontargeting-guide wells per culture and time, giving 48 control wells. Culture is the independent biological replicate for within-context replication, and the declared action class is the pairing unit for each stratum-specific genetic contrast.

Stage 1, Stage 1b, and the Stage 2 confirmatory test partition are therefore disjoint by construction: no intervention that contributes post-challenge identification or transcriptomic variance data before Stage 2 may appear in the Stage 2 test partition, and prechallenge target-engagement measurements for a held-out guide serve only to freeze the external e_j and ρ_j distributions and never include the locked post-challenge endpoint. Relaxing this rule would change the claim to held-out transcriptomic response conditional on prior response calibration, which is not the confirmatory claim proposed here.

The confirmatory claim is restricted accordingly. The 16 genetic targets are a purposively constructed set, not a random sample from a defined population. The primary confirmatory estimand is the average paired method difference across the three known-action held-out classes a_H, a_U, and a_F, represented by HSP90B1, SEL1L, and MBTPS2. Inference is conditional on those three prespecified classes and does not support a population-wide claim about all UPR perturbations. XBP1/a_X and PPP1R15A/a_G are secondary new-action extrapolations. Because no training intervention constrains the response to an a_X or a_G multiplier, those tests challenge the declared intervention functional form as well as the proposed topology; failure cannot be assigned uniquely to one of those two elements. The five-action pooled result is therefore descriptive and secondary. With only three primary action classes, the action level is the binding sample size, so the design requires the target/action pool or culture count to be enlarged before Stage 2 if the prospective power calculation is inadequate rather than lowering the effect threshold. Two off-pathway risks are declared in advance. MBTPS1 and MBTPS2 participate in SREBP processing as well as ATF6 cleavage, so sterol- and lipid-regulatory responses can enter the transcriptome outside the reduced UPR operator; EIF2S1 perturbation can alter global translation beyond the modeled phosphorylated-eIF2α route. These effects are treated as anticipated model discrepancy rather than silently absorbed into kinetic parameters, and mechanistic interpretation for an affected target is withdrawn if the prespecified discrepancy or direct-readout checks fail [103,104,108].

### 7.7. What Stage 3 Can and Cannot Show

A mapping from context to parameters cannot be learned from one training context and then extrapolated to an unseen one, so Stage 3 is baseline-conditioned external transfer without outcome-based parameter refitting rather than learned context generalization. The model is frozen after Stage 2. In the second context, no kinetic, observation, discrepancy, or context parameter is refitted; only the initial-state distribution is recomputed from baseline measurements obtained before intervention. Because the parameters are frozen, they cannot newly become inadmissible. Failure criteria are instead an initial state outside the externally calibrated domain, solver failure or a state trajectory outside biological bounds, a direct-readout check outside its calibrated interval, a predictive interval wider than twice the median training width, or a failed posterior predictive check. One additional cell line or donor supports an external case validation only; it cannot support subgroup calibration or a claim about transport across donors. A learned context mapping is a later Stage 3b analysis requiring at least four independent training contexts before any held-out context is scored [13,45].

### 7.8. Deliberate Exclusions

The first system excludes flux balance analysis, spatial partial differential equations, agent-based tissue modeling, discrete stochastic trajectories, topological alignment, and simultaneous multiscale coupling. These methods suit other questions, but none is needed to decide whether a biologically specified signaling operator adds information to an out-of-distribution transcriptomic prediction. Topological summaries may be examined after the primary analysis with attention to subsampling, filtration, latent dimension, and preprocessing, and their stability under small perturbations does not establish shared causal dynamics [34,35]. A second operator is added only after the single-operator system shows positive mechanistic predictive gain, measurable or explicitly noninterpretable parameters, calibrated predictions, and a reproducible computational advantage relative to its gain, and only through the interface contract of Section 4.3 [5,14,17].

## 8. Locked Evaluation and Falsification Protocol

### 8.1. Registration, Comparators, and Compute

A protocol is prespecified only after it has been deposited in a public dated registry before the relevant outcome data are available. No registry record is claimed by this Perspective. Before biological model fitting, the implementation is to deposit the biological units, partitions, primary estimand, gene modules, equations, priors, parameter bounds, discrepancy rank, solver and tolerance, gradient method, hyperparameter and compute budgets, comparator set, metric orientation, power calculation, and decision rules. The Stage 2 test partition is generated and sealed only after Stage 1 and Stage 1b are complete and the analysis container is frozen, and every later evaluation enters an attempt ledger [15,16,51]. The test manifest records sample identifiers, culture date, passage, treatment preparation, library chemistry, sequencing run, and preprocessing version. Any pretrained encoder checkpoint and its corpus are also fixed with a documented overlap audit against the evaluation context, cell line, tissue, perturbation, and study [37,51,62,63].

The comparator set is designed to isolate the operator rather than to produce a favorable ranking, and every implementation, release tag, checkpoint hash, preprocessing pipeline, and hyperparameter space is to be frozen before test access. General predictive controls are no change, the training-context mean, ridge or elastic-net regression, a frozen encoder with a linear head, a fine-tuned encoder, and a tuned task-specific neural predictor; the additive model enters only for combination tasks. Structural comparators are TxPert, PrePR-CT, and CMonge when their inputs and estimands align [12,28,53]. Operator-focused ablations include the operator alone and a universal differential equation. The four primary operator-isolation controls for mechanistic attribution are late fusion of the same simulated outputs, a capacity-matched unstructured static network, the validation-frozen hard-rewiring ensemble, and a capacity-matched learned-dynamical operator [7,8,54,55,56,57,93]. No comparator may be added, substituted, or upgraded after the test partition is opened.

Comparators are assigned to estimands rather than pooled into one ranking: the general predictive controls and TxPert contest the primary genetic module endpoint, the additive model enters only for combination conditions, CMonge only for the distributional secondary endpoint, and PrePR-CT only for the chemical-response setting to which it applies. Predictive superiority is assessed against the strongest task-compatible general predictor selected on validation data. Mechanistic predictive gain is computed only against the four matched operator-isolation controls, never against a model that estimates a different biological quantity.

The static capacity-matched control shares the identical frozen encoder, initial-state model, intervention inputs, observation map, discrepancy block, and total trainable capacity with the hybrid and replaces only the mechanistic state-equation block with an unstructured map. The matched learned-dynamical control receives the same frozen target-to-action mapping and the same assay-derived a_j distributions as the biologically specified operator, as well as the same chemical intervention functions; otherwise the comparison would confound topology with intervention localization. It also preserves the same ten-state dimension, initial-state model, numerical solver, observation map, discrepancy block, tuning opportunities, and compute allocation. Its internal connectivity and rate coefficients are learned from training data under a prespecified sparsity and parameter-tying rule that leaves exactly the same number of free state-equation coefficients as the biologically specified operator after Stage 1, at most twelve in the primary configuration. Thus, the isolation contrast tests predeclared topology rather than giving either model extra intervention knowledge or operator-block capacity. A dense ten-state learned operator may be reported as an exploratory sensitivity analysis, but it is not used for MPG unless its total trainable capacity can be exactly matched. Late fusion uses the same simulated features without gradient coupling, and the hard-rewiring control preserves the operator family and numerical burden while changing topology. All four isolation controls receive the same accelerator-hour-equivalent budget and validation opportunities.

### 8.2. Endpoints, Power, and the Decision Rule

The primary endpoint will be frozen before the Stage 2 test split so that no scaling or gene-selection choice remains outcome dependent. A 120-gene confirmatory panel will be selected from eligible external and training information after globally excluding all 16 candidate CRISPRi target transcripts and any additional transcript predeclared as directly guide-affected; excluded genes remain available for target-engagement quality control and the direct-knockdown sensitivity model but never enter the primary module score. For module m and time t, the standardized effect is computed within each culture as the mean log2 count per million with a fixed pseudocount of one across the frozen eligible genes, minus the mean of the matched same-culture/time controls, divided by a module- and time-specific scale s_m,t estimated from training controls only. Chemical conditions use vehicle controls and genetic conditions use nontargeting-guide controls. Zero counts remain observations. A well may be excluded only by predeclared library-quality criteria independent of response. For the primary known-action score, observed and predicted eight-component module-time vectors are averaged across n_cult = 6 independent cultures within each of HSP90B1, SEL1L, and MBTPS2, and RMSE is then calculated across those three intervention-level mean vectors. The two new-action targets are scored by the same frozen endpoint definition in a separate secondary analysis, and a five-target pooled summary is reported only as a secondary integrated stress test. Culture-level data are retained for the mixed model and hierarchical uncertainty analysis [11,12,48].

Generalization axes and strata are reported separately rather than collapsed into the primary result. Within the genetic perturbation axis, the known-action and new-action strata defined in Section 7.6 are analyzed separately, and the pooled five-target summary cannot establish which stratum supplied any observed gain. Dose generalization on the chemical axis is a separate prespecified secondary analysis with its own endpoint, comparator, and report; a gain on one axis or stratum is never used to support a claim about another.

All other endpoints are exploratory. Mean absolute error, Spearman correlation, direction agreement, branch classification, negative log-likelihood, interval coverage and width, the continuous ranked probability score, and performance stratified by effect magnitude are reported as a declared panel with Benjamini–Hochberg control at 5 percent across that panel, and no confirmatory claim rests on any of them. Replicate-to-replicate agreement is reported descriptively as a reproducibility benchmark, but it is not used to derive the normalization floor; that floor comes from the variance-components model in Equation (2) [11,12,28,100].

The inferential hierarchy is stated explicitly. Modules and times are correlated outcomes within a well; wells are nested within independently prepared cultures; and each normalized score is calculated from intervention-level mean vectors after averaging the six cultures. The primary estimand is the conditional known-action contrast defined in Section 7.6, not a superpopulation effect for all possible UPR interventions. The culture-level mixed model includes fixed method and intervention/action effects, random culture effects, and an intervention-by-culture random term. The same design-based intervention-by-culture variance enters the noise-floor calculation before division by n_cult. A two-way hierarchical bootstrap over the three primary action classes and cultures reports stability of the confirmatory contrast. The two new-action targets and the five-target pooled summary are reported separately and do not increase the primary action-class sample size. Cells are nested measurement units and do not increase the number of independent action classes or cultures [48,49,50].

The decision rule is fixed rather than deferred. Stage 2a is a prerequisite predictive gate: the hybrid must show a positive normalized advantage over the validation-selected general comparator with the paired 95 percent lower bound above zero and adequate interval calibration. Stage 2b is the primary confirmatory mechanistic-attribution hypothesis and is formally tested only if Stage 2a passes. This serial gatekeeping hierarchy does not split alpha between Stage 2a and Stage 2b; the family-wise multiplicity control applies to the four operator-isolation comparisons within Stage 2b. The minimally important mechanistic predictive gain is prospectively set at 0.25 on the normalized scale of Section 6.6, with sensitivity analyses at 0.15 and 0.35. The 0.25 value is a design judgment, not an empirical result, and represents the smallest share of variance-components-defined predictive headroom considered potentially decision-changing for candidate prioritization. For mechanistic attribution, MPG must exceed 0.25 with its paired lower 95 percent bound above zero and the simultaneous multiple-comparisons-with-the-best intervals must exclude the negligible region for all four isolation controls. These rules are to be publicly registered before Stage 2 test access. The Stage 2b test is to be powered to 80 percent at the 0.25 design effect; the threshold is not lowered if the planned sample is inadequate [10,45,47,48,49,50].

The power calculation is prospective rather than a completed analysis. Stage 1b uses separate pilot cultures and supplies the transcriptomic variance components required for design: residual well variance after matched-control subtraction, intervention-by-culture response variance, module-time covariance, and the variance of paired model differences under training-only resampling. A transparent first-pass planning approximation for the Stage 2b action count is n_action approximately 152 σΔ^2 when the design effect is 0.25, power is 80 percent, and α* = 0.05/4 is used conservatively for the four isolation comparisons. On that approximation, five independent action classes would require σΔ no greater than about 0.18, whereas the three-class primary known-action analysis requires σΔ no greater than about 0.14. These numbers make the design constraint visible but are not the final power calculation: the four-control conjunction, module-time covariance, culture hierarchy, culture averaging, and Stage 2a-to-2b gatekeeping can only make the simulation-based requirement at least as demanding as this first-pass calculation. The closed form is therefore a lower-bound planning screen, not an estimate of the final required pool. If the Stage 1b simulation or parametric-bootstrap analysis shows that 80 percent power cannot be reached for the three-class primary test, the known-action target pool and/or independent culture count must be increased before the Stage 2 partition is generated; the 0.25 threshold is not relaxed.

### 8.3. Ablations, Numerical Verification, and Selective Prediction

The ablation set is fixed before test outcomes are available. Each objective term is removed separately, including the parameter prior, discrepancy regularizer, and any numerical diagnostic used as a loss. The observation map is replaced by a matched unrestricted map to test whether restriction improves transport; the encoder is tested frozen and unfrozen; the operator is tested alone, in late fusion, and in end-to-end coupling; and a universal differential equation tests whether a learned residual improves fit while eroding parameter meaning [93,95]. A matched learned-dynamical operator additionally tests whether a state equation with learned connectivity can equal or exceed the predeclared UPR topology at the same operator-block capacity. Every negative control is refitted under the same positivity and numerical-stability requirements, parameter bounds where applicable, tuning opportunity, compute allocation, and success criteria. Kinetic scales in the randomized-scale control are drawn from plausible but incorrect priors inside the admissible range rather than from deliberately pathological values, and a control draw that fails to integrate is resampled rather than counted as evidence for the proposed topology [25,26,27].

The rewiring ensemble is specified rather than described. For the ten-state signed directed graph, admissible rewirings are generated by double-edge swaps that preserve the in-degree and out-degree of every state, the multiset of edge signs, the number of edges within each sensor branch, the self-loop degradation term on every state, and reachability from every intervention target to at least one observed module. Draws that leave a state unreachable, have no non-negative steady state under the shared bounds, or diverge within 24 h of simulated time are rejected and redrawn. At least 200 admissible distinct rewirings are to be generated, and the ensemble is enlarged until the Monte Carlo standard error of the validation-score 95th percentile is below 0.02 normalized units. Before test access, the top 5 percent by validation score, with a minimum of ten members, are frozen as the hard-rewiring subset; the locked-test comparator is the average predictive distribution or paired score across that frozen subset, not a test-set percentile or maximum. If the full constraint set yields fewer than 200 distinct rewirings, any relaxation is made in a prespecified order and documented before the test partition is opened. The alternative-pathway control is also fixed in advance: a reduced heat-shock operator with the same state count, HSF1-driven chaperone induction, and chaperone-sequestration feedback, a biologically coherent topology that is inappropriate for endoplasmic-reticulum stress.

The synthetic study reproduces the sampling structure of the intended experiment rather than noiseless trajectories. It draws context parameters and initial states from declared distributions, integrates the reduced operator, generates direct readouts and negative-binomial counts, adds batch effects, and omits one known reaction from the fitted model, then evaluates state and parameter coverage, posterior predictive calibration, detection of the omitted reaction through discrepancy or model comparison, and robustness to changes in time points and noise [91,92,97,98]. Numerical verification is part of the same gate: gradients from the selected sensitivity method are compared with carefully scaled finite differences; forward trajectories are reproduced across solver tolerances with accepted and rejected steps, stiffness indicators, positivity violations, and conservation defects recorded; and continuous, discrete-adjoint, and direct sensitivities are compared on a reduced test [64,65].

A binary seen-versus-unseen partition hides how far a new context lies from training, so the protocol reports a distance-stress curve using a threshold that can be estimated independently of the Stage 3 outcome. The primary distance is a standardized one-dimensional projection of the four untreated baseline module scores, with projection direction, scale, and abstention threshold fixed from an external reference panel of at least twenty independently cultured untreated samples generated on the same library chemistry and processed through the same pipeline. The reference panel is assembled before Stage 3, is prespecified with respect to eligible cell contexts, contains no intervention outcomes, and is used only for distance calibration rather than model fitting. A Ledoit–Wolf Mahalanobis distance on that external panel is a sensitivity analysis. Encoder-embedding cosine distance and descriptor-set distance are exploratory sensitivity analyses because latent distances can track batch rather than biology [49,50,61]. The model abstains when the primary distance exceeds the externally calibrated threshold, when the posterior predictive interval exceeds twice the median training width, when discrepancy variance share exceeds 0.30, when a direct-readout check falls outside its calibrated interval, or when the initial-state posterior lies outside the prespecified support. Coverage and error are reported as functions of retained fraction. If the external reference panel is unavailable or assay-incompatible, the distance-based Stage 3 claim is not made [21,96,97].

### 8.4. Evaluation Contract and Interpretation

Table 11 states the locked evaluation contract, separating the first held-out intervention test from later context-transfer and prospective assessments, naming the statistical unit, and tying each claim to its decisive comparator. Table 12 lists the controls needed to interpret a gain and the interpretation that follows when performance is unchanged, together with the conclusion permitted by each combination of predictive and mechanistic outcome. A result is persuasive only when the primary endpoint, calibration, parameter admissibility, mechanistic predictive gain, and compute accounting agree [7,15,16,28]. Predictive and mechanistic conclusions remain separate throughout: a positive prediction with nonidentifiable parameters supports the architecture as a predictor within the tested domain but does not license interpretation of its rate constants, and equal performance from late fusion or a rewired mechanism supports the simpler or less interpretive alternative [15,24,98].

## 9. Translational Boundaries and a Gated Roadmap

Translation begins with a context of use rather than an application catalog. Target prioritization requires prospective improvement in hit rate within a primary or disease-relevant system. Assignment of a mechanism of action requires demonstrated pathway perturbation or rescue rather than an attribution map. Selection of combination therapy requires dose-resolved prospective testing against an additivity model. A digital twin requires individual calibration, longitudinal updating, uncertainty propagation, external validation, and demonstrated influence on a prespecified decision, and a population model conditioned on patient features does not meet that standard even when its outputs look individualized [6,17,28]. Table 13 states what must be shown before each use can be claimed and the gate that governs progression; it does not assert that any threshold has been met.

Data provenance and release governance are part of the same standard, and the three properties at stake are distinct. Reproducibility is the recovery of the reported result from the same data and code. Replicability is the recovery of the finding by an independent team running its own implementation on the same or comparable data. Independent external validation is the confirmation of predictive performance in data the original team never held. Perturbation atlases, harmonized collections, and pretrained checkpoints carry licensing, consent, and reuse conditions that determine which of these are available to whom, and a model that depends on a proprietary graph or an unreleasable checkpoint forfeits open reproducibility while remaining open to replication through independent implementation and to external validation through controlled or federated access [41,43,109,110]. A release should therefore state which components are open, which are restricted, what an independent group can rebuild without the restricted assets, and which reported results survive that restriction. Provenance documentation at the level of dataset datasheets and machine-actionable metadata is the minimum that makes such a statement checkable [109,110,111].

## 10. Limitations

Four limitations bound everything above. The first concerns the evidence synthesis. This is a critical Perspective in which study selection and data extraction were performed by a single author without duplicate screening, rather than a systematic review, so the exact search strings, update procedure, inclusion criteria, and evidence matrix are supplied to make the argument auditable. The search was rerun and broadened on 14 August 2026 after dynamical formulations were found to be underrepresented in the first pass, which itself illustrates the limitations of single-author narrative searching. Publication status and citation metadata for the included fast-moving studies were rechecked on 22 August 2026, and that verification will be repeated immediately before the manuscript is frozen for publication. Direct comparison remains difficult because task, preprocessing, effect-size distribution, statistical unit, and compute differ across studies, so the conclusions are confined to the studies and operator ablations represented here [10,13,45,47,54,55,56,57,58].

The consequences of possible omissions differ by type of claim. The conclusion that simple controls remain competitive, and the conclusion that relational, set-based, transport, and learned-dynamical structure can improve defined tasks, each rest on several independent peer-reviewed studies and would not be reversed by a missed report; an omitted study could add nuance but not remove the pattern. The claim that no identified study isolates the incremental value of a biologically specified operator against the full matched control set is an existence claim and is therefore the most exposed: a single overlooked study meeting the operator-isolation criterion would falsify it. For that reason, the claim is dated to the 22 August 2026 recheck, stated as a description of the identified literature rather than of the field, and accompanied by the criterion itself so that any reader can test a candidate study against it. Such a study would change the framing of Section 7 and Section 8 from first test to replication, but it would not alter the design, whose validity rests on the matched controls rather than on the state of the literature. The most likely direction of bias is toward high-visibility venues and English-language records and away from workshop, thesis, and non-indexed work, which is where the first-pass underrepresentation of dynamical models arose; the broadened rerun corrected that instance but cannot exclude others.

The second limitation is that the proposed framework is itself unvalidated. No pilot, synthetic-recovery, or performance analysis has been carried out, and every numerical threshold in Section 7 and Section 8, including the 0.25 minimally important gain, the 0.30 discrepancy-share bound, the identifiability criteria, the twelve-parameter cap, and the sample sizes, is a prospective design judgment rather than an empirically established standard. Whether twelve kinetic quantities are identifiable at the planned sampling density, whether the paired-difference variance is small enough for three action classes to give 80 percent power, whether at least 200 admissible rewirings exist under the declared constraints, and whether the variance-components floor leaves enough predictive headroom for the endpoint to be informative are empirical questions that Stages 0 and 1 are designed to answer before any confirmatory test. The staging is the mechanism by which the framework can fail cheaply: an infeasible design would be detected, and would be reportable, before new perturbation sequencing is generated. Until an implementation has passed those stages, the framework should be read as a specification of what a mechanistic-attribution claim would require, not as evidence that such a claim can be made.

The third limitation is cost. The full protocol requires a targeted protein time series, a variance pilot, six independent cultures, sixteen guide constructs with independent target-engagement assays, and matched compute across the comparator set, and few laboratories will run it alone. Three considerations bound that demand. First, the demand follows from the claim rather than from the framework: a group that wishes only to report a useful predictor need not run the operator-isolation controls, and the staged design allows work to stop, and to be reported, at any gate. Second, the computational burden is modest in absolute terms; the operator is a ten-state system, the full Stage 2 configuration has about 6000 trainable parameters, and the accelerator-hour ledger is a reporting requirement for fair matching rather than a demand for large-scale compute. Third, several elements can be adopted separately and at low cost by any group publishing a hybrid model, namely the capacity ledger, the distinction between predictive superiority and mechanistic attribution, the attempt ledger, and the late-fusion and rewiring controls. The wet-laboratory components are of the scale of one medium-sized Perturb-seq experiment and are well suited to a consortium or to a benchmark organizer, where the cost of a locked evaluation is shared across the groups whose models it adjudicates.

The fourth limitation is that the field moves faster than any synthesis. The negative benchmark results summarized in Section 3 describe named models, corpora, and tasks evaluated between 2024 and 2026, and some may be superseded. The argument of this Perspective does not depend on their persistence: it depends on the requirement that any gain be measured against the strongest matched comparator available at the time of testing, and a foundation model that comes to predict interventional responses in unseen contexts would enter the design as that comparator rather than undermine it. In the same way, the separation of representation learning from perturbation prediction in Section 3.6 records what has and has not been demonstrated; it does not deny that pretrained representations may prove to be the best route to cross-context transfer, which is the axis on which their contribution would most plausibly appear and which the design defers to a later multi-context stage for that reason.

## 11. Conclusions

Predictive virtual-cell research has reached the point at which architectural ambition should be separated from empirical evidence. Simple controls remain competitive on several out-of-distribution perturbation tasks. Recent gains have come along two strands: set structure, knowledge graphs, cell-type-specific priors, and transport geometry on one side, and learned state equations expressed as neural ordinary differential equations, stationary diffusions, and related learned kinetic systems on the other, the second of which now predicts unseen perturbations and combinations and recovers interpretable regulatory structure [54,55,56,57]. Those results establish that both relational structure and learned dynamics can matter. What remains unestablished is whether a biologically specified topology, declared in advance with constrained semantics and orthogonal measurement, adds predictive information beyond static and learned-dynamical capacity-matched alternatives on a leakage-resistant group-level split under matched compute [7,8,10,12,28,53,58].

The next experiment is therefore smaller than a multiscale virtual cell. It uses one pathway, one biologically specified operator, one primary predictive modality with orthogonal mechanistic readouts, and one primary generalization axis, beginning in a single cellular context before staged external testing. It uses explicit initial-state uncertainty, a separate intervention operator, a restricted observation map, a bounded discrepancy model, a declared capacity ledger, and statistics computed on independent cultures and interventions. It compares biologically specified coupling against late fusion, a static capacity-matched control, a matched learned-dynamical operator, and a validation-frozen hard-rewiring ensemble, and advances through synthetic recovery, direct pathway measurement plus a variance pilot, restricted transcriptomic prediction, external case validation, and a locked prospective batch. This design can support, bound, or refute the hypothesis without redefining success after the outcome is known [15,24,97].

A positive result would justify extending the tested context and adding a second operator only through a declared interface. A null result would be equally informative: it would favor late fusion, relational structure, or a simpler task-specific model, and would quantify the cost of mechanistic coupling without dismissing mechanistic simulation for calibration or hypothesis testing. The value of a predictive virtual cell should be judged by measured transport, calibrated uncertainty, reproducible computation, and the capacity to be falsified, rather than by the number of scales or modalities named in its architecture [5,16,17].

## Figures and Tables

**Table 1:** Evidence dimensions used to interpret studies. The dimensions are not collapsed into one ordinal score because peer-review status, split integrity, comparator adequacy, and biological replication answer different questions.

Dimension	Question Applied to Each Source	Consequence for Interpretation
Publication status	Peer-reviewed article, reviewed conference paper, preprint, software release, or institutional description?	Preprints can identify emerging directions but do not establish a field-wide performance claim.
Target and estimand	Mean response, distributional response, classification, trajectory, pathway state, or phenotype?	Results are compared only when they estimate the same biological quantity.
Generalization axis	What group is absent from fitting: perturbation, combination, dose, time, context, donor, or laboratory?	Performance is not aggregated across different transfer problems.
Statistical unit	Are independent donors, cell lines, organoids, wells, or studies used for inference?	Cell counts alone do not determine confidence or external validity.
Comparator adequacy	Were no-change, mean, additive, linear, task-specific, and structured baselines run on the same split?	A reported gain is bounded by the strongest matched comparator, not by a historical model.
Leakage control	Were gene sets, labels, preprocessing, and pretraining-corpus overlap fixed without test information?	Claims weaken when outcome information can enter representation or scoring.
Reproducibility	Are data, splits, code, checkpoints, versions, and seeds available?	Unreproducible rankings are treated as provisional.
Calibration and validation	Are uncertainty, subgroup coverage, external data, and prospective testing reported?	Retrospective accuracy cannot support decision use without these elements.
Operator isolation	Is the mechanistic operator ablated against late fusion, a matched network, and rewired topology?	Only matched isolation against late fusion, a static capacity control, a learned-dynamical control, and rewired topology can support attribution to the biologically specified operator.

**Table 2:** Nomenclature used in this Perspective and in the proposed minimum hybrid. Units are illustrative and must be replaced by assay-specific and model-specific units in an implementation.

Symbol	Meaning	Typical Unit	Role
y0, y	Observed baseline and post-intervention counts or replicate summaries	counts or normalized abundance	Data entering the initial-state and observation models
c	Cellular context, such as cell line, donor, medium, and batch descriptors	categorical or standardized covariates	Conditions baseline state and parameters
u, d, t	Intervention identity, dose, and observation time	category; concentration; time	Defines the intervention operator
x0, x(t)	Initial and time-dependent mechanistic state	concentration, occupancy, or activity	State integrated by the operator
θ	Context-specific mechanistic parameters	parameter-dependent	Bounded and hierarchically pooled quantities
θI	Intervention potency and occupancy parameters	parameter-dependent	Disjoint from θ; carried by the intervention operator
ψ, η, ω	Neural or probabilistic model parameters	dimensionless after scaling	Initial-state, parameter, observation, or discrepancy models
I(u,d)	Biologically specified intervention operator	operator-dependent	Modifies selected inputs, rates, or initial conditions
δ	Structured model discrepancy	same scale as the observation predictor	Represents missing biology without relabeling it as kinetics
S(M)	Normalized predictive score for model M	dimensionless	Affine transformation of the prespecified raw metric relative to its trivial baseline and variance-components-defined predictive floor; larger values are better
MPG	Mechanistic predictive gain	dimensionless after normalization	Incremental predictive value of the biologically specified operator over matched operator-isolation controls; not an information-theoretic quantity

**Table 3:** Operational taxonomy of biological structure and the claim each category can support. The first two rows are subclasses of one operator category.

Category	Structure Imposed	Legitimate Claim Before Validation	Required Control
Operator, biologically specified	State equation, event process, or constrained optimization with semantics declared before fitting	Predictions and parameters refer to biologically interpretable quantities that are directly observable or independently constrainable, including identifiable combinations	Rewiring ensemble, capacity-matched static network, matched learned-dynamical operator, late fusion
Operator, learned dynamical	State equation of the same type with structure and parameters estimated from data	Responses are represented through an explicitly dynamical state equation whose fitted structure permits post hoc biological interpretation	Matched biologically specified operator and an equal-capacity nondynamical model
Structured architecture	Connectivity restricted by ontology or pathway hierarchy	Interpretable groupings of features	Matched network with permuted grouping
Relational prior	Graph over genes or interventions	Related interventions have related effects	Degree-preserving rewiring and edge deletion
Causal model	Restrictions on stable dependence across environments	Certain associations should persist under intervention	Environment-shuffled or anticausal controls
Geometric regularizer	Constraints on representation shape	Numerical stability of the summary	Subsampling, filtration, and dimension sensitivity
Simulated features	Simulator outputs supplied to a learner	Mechanistic outputs carry predictive information	Matched capacity with mechanism-free features

**Table 4:** Selected evidence bearing directly on out-of-distribution perturbation prediction. Findings are confined to the task and partition used in each study.

Study	Design and Comparator	Main Result Relevant Here	Operator Status
Ahlmann-Eltze et al. [7]	Five cellular foundation models and two deep models versus no-change, additive, and linear controls	No deep model exceeded the controls on the unseen-single and unseen-combination tasks examined	No explicit operator
Wong et al. [8]	Graph and pretrained models versus simple controls on three Perturb-seq datasets	Simple controls won; a curated benchmark label inconsistency was identified	No explicit operator
PertEval-scFM and Csendes et al. [9,46]	Foundation-model embeddings under held-out perturbations and distribution shift	Benefits were limited or inconsistent and depended on the setting	No explicit operator
Systema [45]	Perturbation-specific evaluation after accounting for systematic variation	Common metrics can overestimate prediction by rewarding shared treated–control differences	Evaluation framework
Wei et al. and scArchon [10,47]	27 methods across 29 datasets and six metrics; nine methods across six datasets	Rankings vary by dataset, metric, and generalization axis; context representation remains limiting	Evaluation frameworks
STATE [52]	Set-based transformer versus mean, linear, autoencoder, and encoder baselines	Preprint reports consistent gains in held-out cellular contexts, strongest in context-diverse data	No explicit operator
TxPert [28]	Multiple knowledge graphs versus additive and task-specific baselines	Peer-reviewed gains on defined unseen-perturbation and cross-context tasks	Relational prior
PrePR-CT [53]	Cell-type-specific coexpression graphs in data-limited chemical-response prediction	Improved prediction in unseen cell-type and perturbation settings	Relational prior
CMonge [12]	Conditional optimal transport across drugs, doses, and combinations	Improved distributional prediction for unseen conditions using a global conditional map	Geometric and transport model
Latent causal diffusion [54]	Stationary stochastic differential equation with neural drift versus established distributional predictors	More accurate distributional prediction for unseen perturbation combinations; learns a gene-level dynamical system	Learned-dynamical operator
PerturbODE [55]	Neural ordinary differential equation with the network encoded in its parameters versus linear structural causal models	Improved prediction of held-out transcription-factor overexpression and recovery of network structure	Learned-dynamical operator
LazyNet [56]	One-step neural ordinary differential equation on pre- and post-treatment snapshots versus transformer and state-space baselines at matched budget	Competitive genome-wide accuracy on CPU with interpretable multiplicative parameters	Learned-dynamical operator
CLAMP [57] (GenBio 2026 Spotlight; non-archival)	Mechanistic probe of regulatory structure in frozen single-cell foundation-model representations using a shared differentiable steady-state Hill-kinetics system fitted to Perturb-seq endpoints	A gene-space regulatory adjacency is recovered, and held-out combinatorial perturbations are evaluated; the report is a non-archival workshop Spotlight	Learned-dynamical operator
CellBox [58]	Explicit ordinary differential equations with deep-learning parameter inference versus static network and neural-network models	Prediction of untested drug combinations and transfer to unseen targets; protein and phenotypic readouts, outside this estimand	Biologically specified operator, different estimand
Operator isolation	Biologically specified operator versus late fusion, validation-frozen hard rewirings, a capacity-matched unstructured network, and a capacity-matched learned-dynamical operator on one locked split	No study identified through the 22 August 2026 status recheck isolates this incremental effect under the full design	Evidence gap

**Table 5:** Mechanistic operator families, gradient routes, and the observations that are strongly informative for mechanistic identification in the stated context of use. The listed observations are typical requirements, not universal minima for every implementation.

Operator Family	Constraint and State	Gradient Route	Typical Observations for Identification	Quantity Left Weakly Identified Without Them
Kinetic ODE	Concentrations or activities governed by stoichiometry and rate laws	Direct sensitivity, discrete or continuous adjoint, with finite-difference checks	Time-resolved protein, phosphoprotein, reporter, or targeted RNA data	Activation and turnover rates, branch-specific delays
Kinetic SDE or CME	Probability distribution or integer molecular counts	Reparameterized approximation, score function, coupled finite difference, or surrogate	Single-molecule or population-distribution data revealing intrinsic variability	Burst size and frequency, intrinsic versus extrinsic variance
FBA or dFBA	Feasible fluxes under mass balance, bounds, and an objective	Implicit differentiation through the optimization problem	Exchange flux, isotope tracing, metabolite concentrations, growth demand	Objective validity, bound tightness, degenerate optima
Rule-based	Site-specific complexes and modifications	Compiled network sensitivities or a validated surrogate	Binding, modification, complex, or pathway-output measurements	Site occupancy and combinatorial complex abundance
Logical or Boolean	Discrete regulatory state and attractors	Relaxation, probabilistic estimator, or derivative-free calibration	Qualitative activation patterns and intervention-response logic	Timing, threshold placement, switch sharpness
Reaction–diffusion PDE	Concentration fields, geometry, boundary conditions, transport	Differentiation through a discretized solver or validated neural operator	Spatially resolved concentration or imaging data and measured geometry	Diffusion coefficients, boundary fluxes, localization
Agent-based	Per-cell state, position, mechanics, birth, death, contact	Smoothed population model, event estimator, or surrogate	Longitudinal imaging, lineage, spatial neighborhood, tissue phenotype	Contact rules, motility, division and death rates

**Table 6:** Integration patterns, their primary deliverable, and the control required to interpret a positive result.

Pattern	Primary Deliverable	Control Required
Amortized inference	Fast parameter or posterior estimation with the operator unchanged	Simulation-based calibration and coverage on synthetic data
Universal or residual dynamics	Improved fit with a learned term inside the state equation	Residual-free operator, and parameter stability with and without the residual
Soft residual penalty	Approximate consistency with the equations	Removal of the penalty term, and verification that the defect is not already enforced by the solver
Differentiable simulator or optimization layer	End-to-end training through the operator	Gradient provenance report and finite-difference agreement
Constrained latent or causal representation	Stable representation under environment shift	Environment-shuffled control and matched unconstrained latent
Late fusion	Predictive value of mechanistic outputs	Matched downstream capacity with mechanism-free features

**Table 7:** States in the proposed reduced unfolded-protein-response operator, with the preferred direct observation for each. States whose preferred observation is a protein, phosphoprotein, or reporter measurement remain latent when only RNA is collected. All states are scaled to a declared reference concentration or activity before fitting; numerical bounds and priors are specified in Supplementary File S1 and are to be frozen in the dated protocol before biological model fitting.

Symbol	Biological Quantity	Preferred Direct Observation	Role in the Reduced Operator
U	Unfolded or misfolded protein load in the endoplasmic reticulum	Stress reporter, aggregation-sensitive probe, or calibrated surrogate	Drives sensor activation and consumes folding capacity
P	Scaled active PERK abundance/activity	PERK phosphorylation or kinase reporter	Initiates eIF2α phosphorylation and translational attenuation
E	Scaled phosphorylated eIF2α abundance/activity	Phospho-eIF2α measurement	Reduces global translation and promotes selective ATF4 translation
A	ATF4 activity	ATF4 protein or transcriptional reporter	Controls integrated-stress-response genes and contributes to CHOP induction
I	Scaled active IRE1 abundance/activity	IRE1 phosphorylation or RNase activity	Controls XBP1 splicing and adaptive output
X	Spliced XBP1 activity	XBP1s RNA or protein	Induces folding and degradation programs
F	Nuclear cleaved ATF6 abundance/activity	Cleaved ATF6 or nuclear reporter	Induces chaperone genes and modifies CHOP dynamics
H	Effective chaperone and folding capacity	BiP or GRP78 and selected folding-capacity readouts	Clears unfolded load and mediates negative feedback
C	CHOP-associated terminal-stress signal	CHOP RNA, protein, or reporter	Stress-severity and cell-fate endpoint
G	GADD34-directed eIF2α phosphatase activity	GADD34 RNA or protein and phospho-eIF2α recovery	Delayed negative feedback that restores translation

**Table 8:** Capacity ledger for the Stage 2 configuration. Counts are exact for the prespecified architecture with G = 120 eligible endpoint genes after the predeclared CRISPRi-target exclusions and 10 states; formulas are shown so that capacity follows from the frozen panel and discrepancy specification.

Component	Fixed Before Fitting	Trainable at Stage 2	Count Formula
Pretrained encoder	Checkpoint, corpus, tokenization	0 (frozen)	Identical across all arms; capacity shared, not compared
Initial-state model q_η	Architecture, two hidden layers of width 32, input G + 1	5620	(121 × 32 + 32) + (32 × 32 + 32) + (32 × 20 + 20)
Context model (B, b_c_)	Disabled at Stages 1 and 2	0	Activated only at Stage 3b with at least four training contexts
Mechanistic operator θ	37 kinetic parameters and thresholds; signs and bounds declared	12 at most	Prospective cap; sensitivity analyses at 8 and 16
Chemical intervention operator θI	Chemical target and mathematical action; exposure functions fixed in form	4	2 stressors × (maximal effect, half-maximal concentration)
Genetic action inputs aj	Target-to-process mapping plus assay-derived distributions for ej and ρj, frozen before Stage 2 and never updated from locked outcomes	0	16 externally specified action distributions, one per candidate CRISPRi target; uncertainty is propagated but adds no Stage 2-fitted parameter
Observation map	Module membership, endpoint gene weights, sign of each loading	360	3G: Intercept, one nonzero module loading, dispersion per gene
Discrepancy δ	Rank 3, regularization operator R, latent δ_rt_ marginalized	369	3G loadings + 3 diagonal scales + 3 × 2 offsets
Total	Trainable structure, bounds, priors, and frozen 120-gene endpoint panel	6365	5620 + 12 + 4 + 360 + 369; up to 16 biologically specified trainable kinetic/chemical quantities plus 16 fixed genetic action inputs

**Table 9:** Prospective mapping of every candidate genetic intervention to the single operator action it may modify. For each target, a_j = 1 − ρ_j e_j is represented by an assay-derived distribution frozen before Stage 2; no action parameter is estimated from the locked response. The primary confirmatory stratum contains three known-action classes represented in training; two additional held-out targets form a secondary new-action stratum.

Interference Target	Declared Operator Action	State/Process Affected	Partition and Independent Eligibility Evidence
EIF2AK3 (PERK)	Attainable PERK pool aP	P	Training; Stage 1 calibration; proximal PERK assay
ERN1 (IRE1)	Attainable IRE1 pool aI	I	Training; Stage 1 calibration; IRE1/XBP1s assay
ATF6	Attainable ATF6 pool aF	F	Training; Stage 1 calibration; cleaved-ATF6 assay
EIF2S1	Available eIF2α substrate aE	E	Validation; phospho-eIF2α/target-engagement assay; declared global-translation off-pathway risk
ATF4	ATF4 production multiplier aA	A	Training; ATF4 protein/reporter check
DDIT3 (CHOP)	CHOP production multiplier aC	C	Training; CHOP protein/reporter check
HSPA5 (BiP)	Effective-chaperone production multiplier aH on the full H input block	H	Training; prechallenge folding/chaperone assay defines the ρj distribution
EDEM1	ERAD-clearance multiplier aU on kU	U	Training; prechallenge ERAD assay defines the ρj distribution
XBP1	XBP1s production multiplier aX	X	Held-out test; new action class aX; secondary extrapolation; XBP1-splicing assay defines ρ_j
PPP1R15A (GADD34)	Inducible phosphatase-production multiplier aG	G	Held-out test; new action class aG; secondary extrapolation; GADD34/phospho-eIF2α recovery assay defines ρ_j
MBTPS1	ATF6 cleavage/availability multiplier aF	F	Validation; ATF6-cleavage assay; declared SREBP-processing off-pathway risk
HSP90B1	Effective-chaperone production multiplier aH on the full H input block	H	Held-out test; known action class aH, new gene; independent functional assay defines ρ_j
PDIA4	Effective-chaperone production multiplier aH on the full H input block	H	Validation; independent functional assay defines ρj
DNAJB11	Effective-chaperone production multiplier aH on the full H input block	H	Training; independent functional assay defines the ρ_j distribution
SEL1L	ERAD-clearance multiplier aU on kU	U	Held-out test; known action class aU, new gene; independent ERAD assay defines ρ_j
MBTPS2	ATF6 cleavage/availability multiplier aF	F	Held-out test; known action class aF, new gene; independent cleavage assay; declared SREBP-processing off-pathway risk

**Table 10:** Stage-gated development of the minimum hybrid. Thresholds are prospective design choices to be deposited in a public dated protocol before confirmatory use. Stage 2a is a prerequisite predictive gate; Stage 2b mechanistic attribution is the primary confirmatory hypothesis.

Stage	Data and Task	Required Analyses	Quantitative Gate
0. Synthetic recovery	Simulated trajectories, replicate counts, batch effects, one omitted reaction	Gradient verification, parameter and state coverage, posterior predictive checks, misspecification detection	Maximum relative gradient error below 0.001; 90 percent credible-interval coverage between 0.85 and 0.95; omitted reaction detected with Bayes factor above 10 or discrepancy variance share above 0.20
1. Direct readouts + variance pilot	Eight-time direct pathway series plus training-only RNA variance pilot in separate pilot cultures at the two selected endpoint times	Identifiability, posterior calibration, intervention-action validation, endpoint-time selection, module-time covariance and variance-component estimation	Identifiability criteria met; at most twelve free kinetic quantities; two endpoint times frozen; variance-components noise floor and 80 percent power design estimable before Stage 2
2a. Restricted transcriptomics, predictive gate	Five held-out genetic targets total: three known-action targets form the primary test, and two new-action targets form a secondary analysis; six Stage 2 cultures, two frozen times, one context	Primary prediction on the three known-action targets against the fixed general comparator; separate secondary new-action and pooled five-target reports; likelihood calibration; observation-map ablation	Normalized predictive advantage over the fixed comparator with paired 95 percent lower bound above zero; predictive-interval coverage between 0.85 and 0.95
2b. Mechanistic-attribution gate	Primary known-action partition after Stage 2a passes; new-action stratum analyzed secondarily	Primary confirmatory MPG across HSP90B1, SEL1L, and MBTPS2; simultaneous comparison with four isolation controls; discrepancy analysis; separate new-action analysis	Primary confirmatory gate: MPG > 0.25 with paired lower 95 percent bound > 0; simultaneous interval excludes the negligible region for late fusion, hard rewirings, capacity-matched static network, and matched learned-dynamical operator; discrepancy share < 0.30
3. Second-context external validation	One additional cell line or donor, model frozen, context terms inactive	Baseline-conditioned external prediction, distance-stress analysis, direct-readout checks	Normalized gain above 0.15 with coverage in band, initial states inside the calibrated domain, no solver failure or out-of-bound trajectory
4. Prospective locked batch	New independently generated cultures after freezing	Frozen code and weights, attempt ledger, publicly preregistered analysis, prospective intervals	Prespecified primary interval excludes the negligible region; coverage criterion met; no post hoc model change

**Table 11:** Locked evaluation contract. Numerical margins are prospective design choices; variance components needed for normalization and power are estimated in Stage 1b and frozen before Stage 2 test access.

Claim	Partition and Unit	Primary Comparison and Endpoint	Required Supporting Evidence	Refutation
Held-out intervention prediction	Three primary known-action held-out targets plus two secondary new-action targets; wells nested in six independent Stage 2 cultures; action class is the pairing unit	Primary: Full hybrid versus the validation-selected task-compatible general predictor on culture-averaged RMSE across HSP90B1, SEL1L, and MBTPS2; secondary: XBP1, PPP1R15A, and pooled five-target summaries	Exploratory metric panel, variance-components noise floor, interval calibration, attempt ledger	Predictive advantage has paired lower 95 percent bound ≤ 0 or calibration gate fails
Mechanistic interpretation	Same Stage 2 partition with Stage 1 orthogonal pathway measurements and prespecified target-action evidence	Profile-likelihood/posterior identifiability, parameter admissibility, bounded discrepancy	State coverage, posterior predictive checks, intervention-action validation	Any free kinetic quantity fails the Stage 1 criteria, discrepancy share exceeds 0.30, or interpretation is prior-sensitive
Mechanistic predictive gain	Paired scores on the three prespecified primary known-action classes; new-action MPG reported separately as secondary	Hybrid versus late fusion, validation-frozen hard rewirings, capacity-matched static network, and matched learned-dynamical operator	Paired design-unit interval, matched compute, multiple-comparisons-with-the-best interval	MPG < 0.25 or the simultaneous interval fails to exclude the negligible region for any isolation control
External case validation	One additional cell line or donor; model frozen; context terms inactive; initial state updated from baseline only	Frozen hybrid versus the fixed general predictor and late fusion	Externally calibrated context-distance stress curve, direct-readout checks, predictive coverage	Gain < 0.15, coverage failure, baseline state outside calibrated domain, solver/trajectory failure, or failed direct-readout/posterior predictive check
Prospective reproducibility	New locked batch or site after model and analysis freezing	Frozen model and analysis versus the prespecified comparator	Sample and test manifests, prospective coverage, no post hoc model change	Prespecified predictive or calibration criterion is not met

**Table 12:** Mandatory controls, the question each answers, and the conclusion permitted when performance is unchanged.

Control	Question Answered	Interpretation If Performance Is Unchanged
Operator alone	Does the curated operator predict without a learned representation?	The learned component may be unnecessary
Late fusion	Does gradient coupling add value beyond the same simulated features?	Tighter coupling is not justified
Capacity-matched unstructured network	Is the gain due to capacity rather than structure?	No evidence that mechanistic restriction adds information
Rewiring ensemble	Does the declared biological topology matter?	Graph architecture or regularization, rather than the declared biological topology, drives the result
Sign and direction scrambling	Are activating, inhibitory, and directional relations informative?	Edge semantics contribute nothing measurable
Wrong plausible pathway	Would any biological-looking prior work?	The result supports generic regularization rather than the declared mechanism
Randomized kinetic scales	Do physically plausible rates matter?	Topology alone carries the result and kinetic interpretation is unsupported
Observation-map ablation	Is the operator or the decoder producing the gain?	An unrestricted map is doing the predictive work
Discrepancy ablation and prior sensitivity	Is structural error represented rather than hidden?	Parameter interpretations are unstable or the operator is misspecified
Encoder frozen versus unfrozen	Is pretraining transfer useful under equal tuning?	Pretraining adds no value or introduces contamination and instability
Matched learned-dynamical operator	Does predeclared biological topology add value beyond an equally sized learned state equation?	If performance is equal or better, the result supports dynamics but not incremental value from predeclared biological structure

**Table 13:** Evidence thresholds and gating for proposed uses of predictive virtual cells.

Proposed Use	Minimum Evidence Before the Use Can Be Claimed	Gate Before the Next Expansion
Target prioritization	Prospective hit-rate improvement in a primary or disease-relevant system under a publicly registered protocol	Calibrated abstention and prospective predictive improvement in the tested context; positive mechanistic predictive gain is required only for the further claim that mechanistic coupling supplied the improvement
Mechanism-of-action assignment	Demonstrated pathway perturbation or rescue with direct readouts, not attribution maps	Identifiable or explicitly reduced parameters at Stage 1
Combination selection	Dose-resolved prospective testing against an additivity model	Passing dose and time generalization as separate experiments
Context extension to primary cells	External prediction across several independent donors or primary systems with context-level calibration	A later multi-context Stage 3b analysis shows persistent gain, calibrated uncertainty, and no systematic out-of-domain failure
Second operator or second scale	Declared interface contract with quantified splitting error and propagated uncertainty	Reproducible compute advantage relative to the gain achieved
Individual digital twin	Longitudinal calibration, uncertainty propagation, external validation, and demonstrated decision impact	Prospective locked-batch success and a fit-for-purpose model credibility assessment for the declared context of use, following the verification, validation, and uncertainty-quantification framework used for in silico evidence [15,16]

## Data Availability

No new experimental data were generated, no predictive model was implemented, and no pilot, synthetic-recovery, or performance analysis has been carried out. Numerical thresholds in Section 7 and Section 8 are prospective design choices rather than results. The protocol is intended to be deposited in a public dated registry before any confirmatory Stage 2 outcome data are accessible, with sensitivity analyses prespecified for thresholds whose choice can materially affect interpretation. All primary sources are cited with digital object identifiers or persistent preprint/proceedings records, and publication status for the rapidly changing 2025–2026 records was rechecked on 22 August 2026. Supplementary File S1 supplies the search records, evidence matrix, authentication ledger, UPR specification, sampling and power templates, intervention mapping, discrepancy specification, comparator definitions, solver/compute checklist, and cross-scale interface contract. The schematics in Figure 1, Figure 2, Figure 3, Figure 4, Figure 5 and Figure 6 are original explanatory artwork prepared for this article.

## Supplementary Materials

The following supporting information can be downloaded at: https://www.mdpi.com/article/10.3390/cells15181711/s1, Supplementary File S1 contains the database-ready search strings, expanded evidence-extraction matrix, updated reference-authentication ledger, complete design-level probabilistic specification, UPR state and intervention tables, Stage 1 and Stage 1b sampling plan, module-gene panel freeze template and target-to-operator mapping records, discrepancy factorization, prospective statistical and power-analysis templates, comparator definitions, solver and compute checklist, minimum reporting checklist, and cross-scale interface contract. Implementation-specific numerical values that depend on Stage 1 measurements are identified as fields to be frozen in the public protocol rather than presented as observed results.

## Appendix A. Search Strategy and Update Procedure

The literature search was first run to 29 July 2026 and was rerun and broadened on 14 August 2026 using PubMed, Scopus, bioRxiv, arXiv, publisher sites, and backward and forward citation chains. MEDLINE records were reached through PubMed rather than through a separate interface, so MEDLINE is not listed as an independent database. Foundational sources in mathematics and systems biology were not date-restricted. For cellular representation models, benchmarks, and hybrid perturbation predictors, the search period began in 2015. This is not a systematic or exhaustive investigation and no PRISMA flow count is claimed. Publication status and citation metadata for included 2025–2026 records were rechecked on 22 August 2026. Table A1 documents the reproducible query families, including the dynamical-systems family added in the August update that recovered the studies discussed in Section 3.5 [5,7,10,13,54,55,56,57,58].

**Table A1:** Reproducible search families. Database syntax was adapted without changing the concepts.

Source	Search String or Update Procedure	Purpose
PubMed	(“virtual cell”[Title/Abstract] OR “whole-cell model”[Title/Abstract] OR “cell state model”[Title/Abstract]) AND (mechanistic[Title/Abstract] OR “foundation model”[Title/Abstract] OR transformer[Title/Abstract] OR “differentiable simulation”[Title/Abstract]) AND (perturbation[Title/Abstract] OR “out-of-distribution”[Title/Abstract] OR benchmark[Title/Abstract])	Locate reviews, empirical models, and benchmarks connecting virtual-cell concepts to perturbation prediction
PubMed	(“single-cell”[Title/Abstract] AND perturbation[Title/Abstract]) AND (benchmark[Title/Abstract] OR baseline[Title/Abstract] OR generalization[Title/Abstract] OR extrapolation[Title/Abstract])	Locate evaluations of perturbation response prediction and simple comparators
Scopus	TITLE-ABS-KEY((“single-cell” OR “cellular foundation model”) AND perturbation AND (benchmark OR “out-of-distribution” OR baseline OR calibration))	Broaden citation and conference coverage for benchmark and evaluation studies
Scopus	TITLE-ABS-KEY((mechanistic OR “physics-informed” OR “neural differential equation” OR “universal differential equation” OR “simulation-based inference”) AND cell* AND (hybrid OR learning OR prediction))	Locate mechanistic and machine-learning integration methods relevant to cellular systems
PubMed and Scopus, dynamical family added 14 August 2026	(“single-cell” OR “Perturb-seq” OR “perturbation”) AND (“neural ODE” OR “neural ordinary differential equation” OR “ordinary differential equation” OR “stochastic differential equation” OR diffusion OR “steady-state ODE” OR “gene regulatory network” OR “dynamical system” OR dynamics)	Recover learned state-equation models of perturbation response that the first pass missed
bioRxiv	“single-cell” perturbation prediction benchmark; “foundation model” perturbation; “virtual cell” mechanistic; “differentiable” systems biology	Locate current preprints and identify later versions of record
arXiv	all:(single-cell perturbation benchmark) OR all:(mechanistic machine learning cell) OR all:(virtual cell foundation model)	Locate machine-learning and numerical-method preprints not indexed in biomedical databases
Publisher/Crossref status recheck (22 August 2026)	Exact title, DOI, proceedings, and author searches for every included preprint or online-first record; replace with a version of record when available	Verify publication status, authorship, pagination, DOI/URL, and conference or workshop status immediately before submission
Citation chaining	Backward references and forward citations for every benchmark, explicit operator method, and minimum-system precedent	Locate contradictory findings, methodological precedents, and primary support for quantitative claims

Records entered the evidence matrix only when task, partition, comparator, and findings could be established from the primary report. Application papers without a meaningful control were used as architectural or measurement precedents rather than as evidence of superiority. Preprints are labeled at each material use, and a version of record supersedes the preprint when available. Quantitative claims were checked against the primary report rather than an institutional announcement. Study selection and data extraction were carried out by one author, which remains a limitation despite the explicit query and evidence records [43,51].
