# Supplementary material for: Explicit Mechanistic Operators in Predictive Virtual Cells: Evidence, Failure Modes, and a Minimum Falsification Framework for Single-Cell Perturbation Prediction
Source: Cells. 2026 Sep 20;15(18):1711. doi: 10.3390/cells15181711 (PMC13605750; doi:10.3390/cells15181711)
Supplement: Supplementary file 1 [file cells-15-01711-s001.zip › cells-4555170-supplementary.pdf]

Supplementary Materials

Explicit Mechanistic Operators in Predictive Virtual Cells: Evidence, Failure Modes, and a Minimum Falsification Framework for Single-Cell Perturbation Prediction

Mikołaj Stańczak 1* Sarfaraz K. Niazi 2

1. *School of Mathematical and Computer Science, Heriot-Watt University, Edinburgh EH14 4AS, UK*
2. *College of Pharmacy and Pharmaceutical Sciences, Washington State University, Spokane, WA 99202, USA*

* Correspondence: mikolajstanczak@wp.pl

Supplementary File S1

This synchronized supplement supports the final manuscript and records the design-level details needed to audit its literature synthesis and proposed unfolded-protein-response (UPR) falsification framework. It does not report experimental or computational performance. The literature search itself was rerun through 14 August 2026, and publication status and citation metadata for fast-moving records were rechecked on 22 August 2026. Numerical values that depend on Stage 1 or Stage 1b observations are explicitly treated as prospective fields to be frozen in a public dated protocol before confirmatory Stage 2 test access.

S1. Scope, Provenance, and Relationship to the Main Manuscript

The supplement follows the manuscript's narrow context of use: prediction of measurable single-cell or replicate-level transcriptomic responses to declared genetic or chemical interventions. It distinguishes biologically specified operators from learned dynamical operators and from nonoperator sources of structure. Its purpose is to make the proposed comparison reproducible without converting the Perspective into an implemented performance study.

Main-manuscript element	Supplementary record	Purpose
Search and evidence appraisal	Sections S2-S4	Reproduce the search concepts, evidence classification, and reference authentication.
Minimum UPR hybrid	Sections S5-S6	Specify states, intervention actions, sampling, biological units, and data provenance.
Evaluation and falsification contract	Section S7	Fix the confirmatory hierarchy, noise-floor construction, MPG controls, and interpretation rules.
Numerical and compute requirements	Section S8	Specify solver, gradient, capacity, tuning, and compute reporting.
Gene-panel freeze record	Section S9	Provide the template used to freeze the 120-gene panel before locked testing.
Future multiscale extension	Section S10	State the interface contract required before adding a second operator or scale.
Readiness status	Section S11	Separate completed manuscript elements from prospective actions required before implementation.

S2. Database-Ready Search Strategy and Update Procedure

The search is a reproducible critical-review update rather than a systematic-review prevalence estimate. Comparative evidence is retained only when task, split, comparator, and reported finding can be established from the primary record. Preprints are labeled at material use and replaced by a version of record when one is available. The August update deliberately added dynamical-system terms after the first pass underrepresented neural ODE, SDE, and steady-state perturbation models.

Table S2. Search families and update procedures used in the final manuscript.

Source	Search string or update procedure	Purpose

Source	Search string or update procedure	Purpose
PubMed	("virtual cell"[Title/Abstract] OR "whole-cell model"[Title/Abstract] OR "cell state model"[Title/Abstract]) AND (mechanistic[Title/Abstract] OR "foundation model"[Title/Abstract] OR transformer[Title/Abstract] OR "differentiable simulation"[Title/Abstract]) AND (perturbation[Title/Abstract] OR "out-of-distribution"[Title/Abstract] OR benchmark[Title/Abstract])	Locate reviews, empirical models, and benchmarks connecting virtual-cell concepts to perturbation prediction
PubMed	("single-cell"[Title/Abstract] AND perturbation[Title/Abstract]) AND (benchmark[Title/Abstract] OR baseline[Title/Abstract] OR generalization[Title/Abstract] OR extrapolation[Title/Abstract])	Locate evaluations of perturbation response prediction and simple comparators
Scopus	TITLE-ABS-KEY(("single-cell" OR "cellular foundation model") AND perturbation AND (benchmark OR "out-of-distribution" OR baseline OR calibration))	Broaden citation and conference coverage for benchmark and evaluation studies
Scopus	TITLE-ABS-KEY((mechanistic OR "physics-informed" OR "neural differential equation" OR "universal differential equation" OR "simulation-based inference") AND cell* AND (hybrid OR learning OR prediction))	Locate mechanistic and machine-learning integration methods relevant to cellular systems
PubMed and Scopus, dynamical family added 14 August 2026	("single-cell" OR "Perturb-seq" OR "perturbation") AND ("neural ODE" OR "neural ordinary differential equation" OR "ordinary differential equation" OR "stochastic differential equation" OR diffusion OR "steady-state ODE" OR "gene regulatory network" OR "dynamical system" OR dynamics)	Recover learned state-equation models of perturbation response that the first pass missed
bioRxiv	"single-cell" perturbation prediction benchmark; "foundation model" perturbation; "virtual cell" mechanistic; "differentiable" systems biology	Locate current preprints and identify later versions of record
arXiv	all:(single-cell perturbation benchmark) OR all:(mechanistic machine learning cell) OR all:(virtual cell foundation model)	Locate machine-learning and numerical-method preprints not indexed in biomedical databases
Publisher/Crossref status recheck (22 August 2026)	Exact title, DOI, proceedings, and author searches for every included preprint or online-first record; replace with a version of record when available	Verify publication status, authorship, pagination, DOI/URL, and conference or workshop status immediately before submission
Citation chaining	Backward references and forward citations for every benchmark, explicit operator method, and minimum-system precedent	Locate contradictory findings, methodological precedents, and primary support for quantitative claims

For submission, the same families should be rerun on the manuscript-freeze date. The search log should record database or server, interface date and time zone, exact query, filters, result count, duplicate cluster, screening decision, exclusion reason, version-of-record substitution, and checker initials. The 22 August 2026 recheck was a publication-status and citation-metadata verification rather than a new evidence-search cutoff.

S3. Expanded Evidence Matrix

The matrix below reproduces the main evidence table because it is the core audit trail for the manuscript's narrowed evidence-gap claim. Learned dynamical operators are treated as genuine state-equation models; the remaining gap is the lack of a locked operator-isolation experiment against matched late fusion, static capacity, learned-dynamical capacity, and rewired-topology controls.

Table S3. Evidence directly bearing on out-of-distribution perturbation prediction.

Study	Design and comparator	Main result relevant here	Operator status
Ahlmann-Eltze et al. [7]	Five cellular foundation models and two deep models versus no-change, additive, and linear controls	No deep model exceeded the controls on the unseen-single and unseen-combination tasks examined	No explicit operator
Wong et al. [8]	Graph and pretrained models versus simple controls on three Perturb-seq datasets	Simple controls won; a curated benchmark label inconsistency was identified	No explicit operator
PertEval-scFM and Csendes et al. [9,47]	Foundation-model embeddings under held-out perturbations and distribution shift	Benefits were limited or inconsistent and depended on the setting	No explicit operator

Study	Design and comparator	Main result relevant here	Operator status
Systema [46]	Perturbation-specific evaluation after accounting for systematic variation	Common metrics can overestimate prediction by rewarding shared treated-control differences	Evaluation framework
Wei et al. and scArchon [10,48]	27 methods across 29 datasets and six metrics; nine methods across six datasets	Rankings vary by dataset, metric, and generalization axis; context representation remains limiting	Evaluation frameworks
STATE [53]	Set-based transformer versus mean, linear, autoencoder, and encoder baselines	Preprint reports consistent gains in held-out cellular contexts, strongest in context-diverse data	No explicit operator
TxPert [29]	Multiple knowledge graphs versus additive and task-specific baselines	Peer-reviewed gains on defined unseen-perturbation and cross-context tasks	Relational prior
PrePR-CT [54]	Cell-type-specific coexpression graphs in data-limited chemical-response prediction	Improved prediction in unseen cell-type and perturbation settings	Relational prior
CMonge [12]	Conditional optimal transport across drugs, doses, and combinations	Improved distributional prediction for unseen conditions using a global conditional map	Geometric and transport model
Latent causal diffusion [55]	Stationary stochastic differential equation with neural drift versus established distributional predictors	More accurate distributional prediction for unseen perturbation combinations; learns a gene-level dynamical system	Learned dynamical operator
PerturbODE [56]	Neural ordinary differential equation with the network encoded in its parameters versus linear structural causal models	Improved prediction of held-out transcription-factor overexpression and recovery of network structure	Learned dynamical operator
LazyNet [57]	One-step neural ordinary differential equation on pre and post snapshots versus transformer and state-space baselines at matched budget	Competitive genome-wide accuracy on CPU with interpretable multiplicative parameters	Learned dynamical operator
CLAMP [58] (GenBio 2026 Spotlight; non-archival)	Mechanistic probe of regulatory information preserved by frozen single-cell foundation-model representations, using a shared differentiable steady-state Hill-kinetics system fitted to Perturb-seq endpoints	Recovers a gene-space regulatory adjacency and evaluates held-out combinatorial perturbations; workshop report is non-archival	Learned dynamical operator
CellBox [60]	Explicit ordinary differential equations with deep-learning parameter inference versus static network and neural-network models	Prediction of untested drug combinations and transfer to unseen targets; protein and phenotypic readouts, outside this estimand	Biologically specified operator, different estimand
Operator isolation	Biologically specified operator versus late fusion, validation-frozen hard rewirings, a capacity-matched unstructured network, and a capacity-matched learned-dynamical operator on one locked split	No study identified through the 22 August 2026 status recheck isolates this incremental effect under the full design	Evidence gap

S4. Reference-Authentication Ledger

Every main-manuscript reference is listed below with a persistent identifier or stable bibliographic locator when one is present in the citation. The ledger is an authentication aid, not an independent source of scientific claims. Publication-status statements for the rapidly changing 2025-2026 records were rechecked on 22 August 2026.

Table S4-1-22. Reference-authentication ledger, main-manuscript references 1-22.

Ref.	Authenticated citation	Persistent identifier	Status	Function
1	Loew, L.M.; Schaff, J.C. The Virtual Cell: a software environment for computational cell biology. Trends Biotechnol. 2001, 19, 401-406. doi:10.1016/S0167-7799(01)01740-1	DOI 10.1016/S0167-7799(01)01740-1	Peer-reviewed journal/record	Virtual-cell, whole-cell, and digital-twin boundary

Ref.	Authenticated citation	Persistent identifier	Status	Function
2	Karr, J.R.; Sanghvi, J.C.; Macklin, D.N.; Gutschow, M.V.; Jacobs, J.M.; Bolival, B.; Assad-Garcia, N.; Glass, J.I.; Covert, M.W. A whole-cell computational model predicts phenotype from genotype. Cell 2012, 150, 389-401. doi:10.1016/j.cell.2012.05.044	DOI 10.1016/j.cell.2012.05.044	Peer-reviewed journal/record	Virtual-cell, whole-cell, and digital-twin boundary
3	Thornburg, Z.R.; Bianchi, D.M.; Brier, T.A.; Gilbert, B.R.; Earnest, T.M.; Melo, M.C.R.; Safronova, N.; Sáenz, J.P.; Cook, A.T.; Wise, K.S.; et al. Fundamental behaviors emerge from simulations of a living minimal cell. Cell 2022, 185, 345-360. doi:10.1016/j.cell.2021.12.025	DOI 10.1016/j.cell.2021.12.025	Peer-reviewed journal/record	Virtual-cell, whole-cell, and digital-twin boundary
4	Macklin, D.N.; Ahn-Horst, T.A.; Choi, H.; Ruggero, N.A.; Carrera, J.; Mason, J.C.; Sun, G.; Agmon, E.; DeFelice, M.M.; Maayan, I.; et al. Simultaneous cross-evaluation of heterogeneous E. coli datasets via mechanistic simulation. Science 2020, 369, eaav3751. doi:10.1126/scienc...	DOI 10.1126/science.aav3751	Peer-reviewed journal/record	Virtual-cell, whole-cell, and digital-twin boundary
5	Bunne, C.; Roohani, Y.; Rosen, Y.; Gupta, A.; Zhang, X.; Roed, M.; Alexandrov, T.; AlQuraishi, M.; Brennan, P.; Burkhardt, D.B.; et al. How to build the virtual cell with artificial intelligence: priorities and opportunities. Cell 2024, 187, 7045-7063. doi:10.1016/j.cell.2024....	DOI 10.1016/j.cell.2024.11.015	Peer-reviewed journal/record	Virtual-cell, whole-cell, and digital-twin boundary
6	Laubenbacher, R.; Niarakis, A.; Helikar, T.; An, G.; Shapiro, B.; Malik-Sheriff, R.S.; Sego, T.J.; Knapp, A.; Macklin, P.; Glazier, J.A. Building digital twins of the human immune system: toward a roadmap. npj Digit. Med. 2022, 5, 64. doi:10.1038/s41746-022-00610-z	DOI 10.1038/s41746-022-00610-z	Peer-reviewed journal/record	Virtual-cell, whole-cell, and digital-twin boundary
7	Ahlmann-Eltze, C.; Huber, W.; Anders, S. Deep-learning-based gene perturbation effect prediction does not yet outperform simple linear baselines. Nat. Methods 2025, 22, 1657-1661. doi:10.1038/s41592-025-02772-6	DOI 10.1038/s41592-025-02772-6	Peer-reviewed journal/record	Perturbation benchmark, transport, or field synthesis
8	Wong, D.R.; Hill, A.S.; Moccia, R. Simple controls exceed best deep learning algorithms and reveal foundation model effectiveness for predicting genetic perturbations. Bioinformatics 2025, 41, btaf317. doi:10.1093/bioinformatics/btaf317	DOI 10.1093/bioinformatics/btaf317	Peer-reviewed journal/record	Perturbation benchmark, transport, or field synthesis
9	Wenteler, A.; Occhetta, M.; Branson, N.; Curean, V.; Huebner, M.; Dee, W.; Connell, W.; Chung, S.P.; Hawkins-Hooker, A.; Ektefaie, Y.; et al. PertEval-scFM: benchmarking single-cell foundation models for perturbation effect prediction. Proc. Mach. Learn. Res. 2025, 267, 66633-...	https://proceedings.mlr.press/v267/wenteler25a.html	Conference/proceedings	Perturbation benchmark, transport, or field synthesis
10	Wei, Z.; Wang, Y.; Gao, Y.; Wang, S.; Li, P.; Si, D.; Gao, Y.; Wu, S.; Li, D.; Dong, K.; et al. Benchmarking algorithms for generalizable single-cell perturbation response prediction. Nat. Methods 2026, 23, 451-464. doi:10.1038/s41592-025-02980-0	DOI 10.1038/s41592-025-02980-0	Peer-reviewed journal/record	Perturbation benchmark, transport, or field synthesis

Ref.	Authenticated citation	Persistent identifier	Status	Function
11	Bunne, C.; Stark, S.G.; Gut, G.; del Castillo, J.S.; Levesque, M.; Lehmann, K.-V.; Pelkmans, L.; Krause, A.; Rätsch, G. Learning single-cell perturbation responses using neural optimal transport. Nat. Methods 2023, 20, 1759-1768. doi:10.1038/s41592-023-01969-x	DOI 10.1038/s41592-023-01969-x	Peer-reviewed journal/record	Perturbation benchmark, transport, or field synthesis
12	Driessen, A.; Rajwade, D.A.; Harsanyi, B.; Rapsomaniki, M.; Born, J. Conditional Monge Gap enables generalizable single-cell perturbation modelling. Nat. Mach. Intell. 2026, 8, 984-996. doi:10.1038/s42256-026-01242-8	DOI 10.1038/s42256-026-01242-8	Peer-reviewed journal/record	Perturbation benchmark, transport, or field synthesis
13	Dimitrov, D.; Schrod, S.; Rohbeck, M.; Stegle, O. Interpretation, extrapolation and perturbation of single cells. Nat. Rev. Genet. 2026, 27, 349-370. doi:10.1038/s41576-025-00920-4	DOI 10.1038/s41576-025-00920-4	Peer-reviewed journal/record	Perturbation benchmark, transport, or field synthesis
14	Goldberg, A.P.; Szigeti, B.; Chew, Y.H.; Sekar, J.A.; Roth, Y.D.; Karr, J.R. Emerging whole-cell modeling principles and methods. Curr. Opin. Biotechnol. 2018, 51, 97-102. doi:10.1016/j.copbio.2017.12.013	DOI 10.1016/j.copbio.2017.12.013	Peer-reviewed journal/record	Model credibility, standards, and reproducible systems biology
15	Musuamba, F.T.; Skottheim Rusten, I.; Lesage, R.; Russo, G.; Bursi, R.; Emili, L.; Wangorsch, G.; Manolis, E.; Karlsson, K.E.; Kulesza, A.; et al. Scientific and regulatory evaluation of mechanistic in silico drug and disease models in drug development: building model credibil...	DOI 10.1002/psp4.12669	Peer-reviewed journal/record	Model credibility, standards, and reproducible systems biology
16	Viceconti, M.; Pappalardo, F.; Rodriguez, B.; Horner, M.; Bischoff, J.; Musuamba Tshinanu, F. In silico trials: verification, validation and uncertainty quantification of predictive models used in the regulatory evaluation of biomedical products. Methods 2021, 185, 120-127. do...	DOI 10.1016/j.ymeth.2020.01.011	Peer-reviewed journal/record	Model credibility, standards, and reproducible systems biology
17	Ma, C.; Zhang, H.; Rao, Y.; Jiang, X.; Liu, B.; Sun, Z.; Song, Z.; Gao, Y.; Cui, Y.; Liu, X.; Li, Z. AI-driven virtual cell models in preclinical research: technical pathways, validation mechanisms, and clinical translation potential. npj Digit. Med. 2026, 9, 25. doi:10.1038/s...	DOI 10.1038/s41746-025-02198-6	Peer-reviewed journal/record	Model credibility, standards, and reproducible systems biology
18	Harris, L.A.; Hogg, J.S.; Tapia, J.J.; Sekar, J.A.P.; Gupta, S.; Korsunsky, I.; Arora, A.; Barua, D.; Sheehan, R.P.; Faeder, J.R. BioNetGen 2.2: advances in rule-based modeling. Bioinformatics 2016, 32, 3366-3368. doi:10.1093/bioinformatics/btw469	DOI 10.1093/bioinformatics/btw469	Peer-reviewed journal/record	Model credibility, standards, and reproducible systems biology
19	Hucka, M.; Finney, A.; Sauro, H.M.; Bolouri, H.; Doyle, J.C.; Kitano, H.; Arkin, A.P.; Bornstein, B.J.; Bray, D.; Cornish-Bowden, A.; et al. The systems biology markup language (SBML): a medium for representation and exchange of biochemical network models. Bioinformatics 2003,...	DOI 10.1093/bioinformatics/btg015	Peer-reviewed journal/record	Model credibility, standards, and reproducible systems biology

Ref.	Authenticated citation	Persistent identifier	Status	Function
20	Keating, S.M.; Waltemath, D.; König, M.; Zhang, F.; Dräger, A.; Chaouiya, C.; Bergmann, F.T.; Finney, A.; Gillespie, C.S.; Helikar, T.; et al. SBML Level 3: an extensible format for the exchange and reuse of biological models. Mol. Syst. Biol. 2020, 16, e9110. doi:10.15252/msb...	DOI 10.15252/msb.20199110	Peer-reviewed journal/record	Model credibility, standards, and reproducible systems biology
21	Waltemath, D.; Adams, R.; Bergmann, F.T.; Hucka, M.; Kolpakov, F.; Miller, A.K.; Moraru, I.I.; Nickerson, D.; Sahle, S.; Snoep, J.L.; Le Novère, N. Reproducible computational biology experiments with SED-ML: the Simulation Experiment Description Markup Language. BMC Syst. Biol...	DOI 10.1186/1752-0509-5-198	Peer-reviewed journal/record	Model credibility, standards, and reproducible systems biology
22	Schmiester, L.; Schälte, Y.; Bergmann, F.T.; Camba, T.; Dudkin, E.; Egert, J.; Fröhlich, F.; Fuhrmann, L.; Hauber, A.L.; Kemmer, S.; et al. PEtab: interoperable specification of parameter estimation problems in systems biology. PLoS Comput. Biol. 2021, 17, e1008646. doi:10.137...	DOI 10.1371/journal.pcbi.1008646	Peer-reviewed journal/record	Model credibility, standards, and reproducible systems biology

Table S4-23-44. Reference-authentication ledger, main-manuscript references 23-44.

Ref.	Authenticated citation	Persistent identifier	Status	Function
23	Wieland, F.G.; Hauber, A.L.; Rosenblatt, M.; Tönsing, C.; Timmer, J. On structural and practical identifiability. Curr. Opin. Syst. Biol. 2021, 25, 60-69. doi:10.1016/j.coisb.2021.03.005	DOI 10.1016/j.coisb.2021.03.005	Peer-reviewed journal/record	Identifiability and parameter inference
24	Villaverde, A.F.; Barreiro, A.; Papachristodoulou, A. Structural identifiability of dynamic systems biology models. PLoS Comput. Biol. 2016, 12, e1005153. doi:10.1371/journal.pcbi.1005153	DOI 10.1371/journal.pcbi.1005153	Peer-reviewed journal/record	Identifiability and parameter inference
25	Raue, A.; Kreutz, C.; Maiwald, T.; Bachmann, J.; Schilling, M.; Klingmüller, U.; Timmer, J. Structural and practical identifiability analysis of partially observed dynamical models by exploiting the profile likelihood. Bioinformatics 2009, 25, 1923-1929. doi:10.1093/bioinforma...	DOI 10.1093/bioinformatics/btp358	Peer-reviewed journal/record	Identifiability and parameter inference
26	Ma, J.; Yu, M.K.; Fong, S.; Ono, K.; Sage, E.; Demchak, B.; Sharan, R.; Ideker, T. Using deep learning to model the hierarchical structure and function of a cell. Nat. Methods 2018, 15, 290-298. doi:10.1038/s41592-018-0032-0	DOI 10.1038/s41592-018-0032-0	Peer-reviewed journal/record	Structured, causal, topological, or late-fusion methodology
27	Elmarakeby, H.A.; Hwang, J.; Arafeh, R.; Crowdis, J.; Gang, S.; Liu, D.; AlDubayan, S.H.; Salari, K.; Kregel, S.; Richter, C.; et al. Biologically informed deep neural network for prostate cancer discovery. Nature 2021, 598, 348-352. doi:10.1038/s41586-021-03922-4	DOI 10.1038/s41586-021-03922-4	Peer-reviewed journal/record	Structured, causal, topological, or late-fusion methodology
28	Fortelny, N.; Bock, C. Knowledge-primed neural networks enable biologically interpretable deep learning on single-cell sequencing data. Genome Biol. 2020, 21, 190. doi:10.1186/s13059-020-02100-5	DOI 10.1186/s13059-020-02100-5	Peer-reviewed journal/record	Structured, causal, topological, or late-fusion methodology

Ref.	Authenticated citation	Persistent identifier	Status	Function
29	Wenkel, F.; Tu, W.; Masschelein, C.; Shirzad, H.; Hodgson, L.; Bendidi, I.; Eastwood, C.; Whitfield, S.T.; Russell, C.; El Mesbahi, Y.; et al. TxPert: using multiple knowledge graphs for prediction of transcriptomic perturbation effects. Nat. Biotechnol. 2026, published online...	DOI 10.1038/s41587-026-03113-4	Peer-reviewed journal/record	Structured, causal, topological, or late-fusion methodology
30	Roohani, Y.; Huang, K.; Leskovec, J. Predicting transcriptional outcomes of novel multigene perturbations with GEARS. Nat. Biotechnol. 2024, 42, 927-935. doi:10.1038/s41587-023-01905-6	DOI 10.1038/s41587-023-01905-6	Peer-reviewed journal/record	Structured, causal, topological, or late-fusion methodology
31	Pearl, J. Causality: Models, Reasoning, and Inference, 2nd ed.; Cambridge University Press: Cambridge, UK, 2009; ISBN 978-0-521-89560-6.	ISBN 978-0-521-89560-6	Book	Structured, causal, topological, or late-fusion methodology
32	Schölkopf, B.; Locatello, F.; Bauer, S.; Ke, N.R.; Kalchbrenner, N.; Goyal, A.; Bengio, Y. Toward causal representation learning. Proc. IEEE 2021, 109, 612-634. doi:10.1109/JPROC.2021.3058954	DOI 10.1109/JPROC.2021.3058954	Peer-reviewed journal/record	Structured, causal, topological, or late-fusion methodology
33	Peters, J.; Bühlmann, P.; Meinshausen, N. Causal inference by using invariant prediction: identification and confidence intervals. J. R. Stat. Soc. Ser. B 2016, 78, 947-1012. doi:10.1111/rssb.12167	DOI 10.1111/rssb.12167	Peer-reviewed journal/record	Structured, causal, topological, or late-fusion methodology
34	Lopez, R.; Tagasovska, N.; Ra, S.; Cho, K.; Pritchard, J.; Regev, A. Learning causal representations of single cells via sparse mechanism shift modeling. In Proceedings of the 2nd Conference on Causal Learning and Reasoning, 2023, 213, 662-691. arXiv:2211.03553.	arXiv:2211.03553.	Conference/proceedings	Structured, causal, topological, or late-fusion methodology
35	Carlsson, G. Topology and data. Bull. Am. Math. Soc. 2009, 46, 255-308. doi:10.1090/S0273-0979-09-01249-X	DOI 10.1090/S0273-0979-09-01249-X	Peer-reviewed journal/record	Structured, causal, topological, or late-fusion methodology
36	Cohen-Steiner, D.; Edelsbrunner, H.; Harer, J. Stability of persistence diagrams. Discrete Comput. Geom. 2007, 37, 103-120. doi:10.1007/s00454-006-1276-5	DOI 10.1007/s00454-006-1276-5	Peer-reviewed journal/record	Structured, causal, topological, or late-fusion methodology
37	Nave, O.; Elbaz, M. Artificial immune system features added to breast cancer clinical data for machine learning (ML) applications. Biosystems 2021, 202, 104341. doi:10.1016/j.biosystems.2020.104341	DOI 10.1016/j.biosystems.2020.104341	Peer-reviewed journal/record	Structured, causal, topological, or late-fusion methodology
38	Adamson, B.; Norman, T.M.; Jost, M.; Cho, M.Y.; Nuñez, J.K.; Chen, Y.; Villalta, J.E.; Gilbert, L.A.; Horlbeck, M.A.; Hein, M.Y.; et al. A multiplexed single-cell CRISPR screening platform enables systematic dissection of the unfolded protein response. Cell 2016, 167, 1867-188...	DOI 10.1016/j.cell.2016.11.048	Peer-reviewed journal/record	Perturbation resource, benchmark, or relational/context model
39	Norman, T.M.; Horlbeck, M.A.; Replogle, J.M.; Ge, A.Y.; Xu, A.; Jost, M.; Gilbert, L.A.; Weissman, J.S. Exploring genetic interaction manifolds constructed from rich single-cell phenotypes. Science 2019, 365, 786-793. doi:10.1126/science.aax4438	DOI 10.1126/science.aax4438	Peer-reviewed journal/record	Perturbation resource, benchmark, or relational/context model
40	Replogle, J.M.; Saunders, R.A.; Pogson, A.N.; Hussmann, J.A.; Lenail, A.; Guna, A.; Mascibroda, L.; Wagner, E.J.; Adelman, K.; Lithwick-Yanai, G.; et al. Mapping information-rich genotype-phenotype landscapes with genome-scale Perturb-seq. Cell 2022, 185, 2559-2575. doi:10.101...	DOI 10.1016/j.cell.2022.05.013	Peer-reviewed journal/record	Perturbation resource, benchmark, or relational/context model

Ref.	Authenticated citation	Persistent identifier	Status	Function
41	Srivatsan, S.R.; McFaline-Figueroa, J.L.; Ramani, V.; Saunders, L.; Cao, J.; Packer, J.; Pliner, H.A.; Jackson, D.L.; Daza, R.M.; Christiansen, L.; et al. Massively multiplex chemical transcriptomics at single-cell resolution. Science 2020, 367, 45-51. doi:10.1126/science.aax6234	DOI 10.1126/science.aax6234	Peer-reviewed journal/record	Perturbation resource, benchmark, or relational/context model
42	Peidli, S.; Green, T.D.; Shen, C.; Gross, T.; Min, J.; Garda, S.; Yuan, B.; Schumacher, L.J.; Taylor-King, J.P.; Marks, D.S.; et al. scPerturb: harmonized single-cell perturbation data. Nat. Methods 2024, 21, 531-540. doi:10.1038/s41592-023-02144-y	DOI 10.1038/s41592-023-02144-y	Peer-reviewed journal/record	Perturbation resource, benchmark, or relational/context model
43	Zhang, J.; Ubas, A.A.; de Borja, R.; Svensson, V.; Thomas, N.; Thakar, N.; Lai, I.; Winters, A.; Khan, U.; Jones, M.G.; et al. Tahoe-100M: a giga-scale single-cell perturbation atlas for context-dependent gene function and cellular modeling. bioRxiv 2025 [preprint]. doi:10.110...	DOI 10.1101/2025.02.20.639398	Preprint	Perturbation resource, benchmark, or relational/context model
44	Heumos, L.; Ji, Y.; May, L.; Green, T.D.; Peidli, S.; Zhang, X.; Wu, X.; Ostner, J.; Schumacher, A.; Hrovatin, K.; et al. Pertpy: an end-to-end framework for perturbation analysis. Nat. Methods 2026, 23, 350-359. doi:10.1038/s41592-025-02909-7	DOI 10.1038/s41592-025-02909-7	Peer-reviewed journal/record	Perturbation resource, benchmark, or relational/context model

Table S4-45-66. Reference-authentication ledger, main-manuscript references 45-66.

Ref.	Authenticated citation	Persistent identifier	Status	Function
45	Nadig, A.; Replogle, J.M.; Pogson, A.N.; Murthy, M.; McCarroll, S.A.; Weissman, J.S.; Robinson, E.B.; O'Connor, L.J. Transcriptome-wide analysis of differential expression in perturbation atlases. Nat. Genet. 2025, 57, 1228-1237. doi:10.1038/s41588-025-02169-3	DOI 10.1038/s41588-025-02169-3	Peer-reviewed journal/record	Perturbation resource, benchmark, or relational/context model
46	Vinas Torne, R.; Wiatrak, M.; Piran, Z.; Fan, S.; Jiang, L.; Teichmann, S.A.; Nitzan, M.; Brbic, M. Systema: a framework for evaluating genetic perturbation response prediction beyond systematic variation. Nat. Biotechnol. 2026, 44, 1050-1059. doi:10.1038/s41587-025-02777-8	DOI 10.1038/s41587-025-02777-8	Peer-reviewed journal/record	Perturbation resource, benchmark, or relational/context model
47	Csendes, G.; Sanz, G.; Szalay, K.Z.; Szalai, B. Benchmarking foundation cell models for post-perturbation RNA-seq prediction. BMC Genomics 2025, 26, 393. doi:10.1186/s12864-025-11600-2	DOI 10.1186/s12864-025-11600-2	Peer-reviewed journal/record	Perturbation resource, benchmark, or relational/context model
48	Radig, J.; Droit, R.; Doncevic, D.; Li, A.; Bui, D.T.; Herfurth, L.; Kuhn, T.; Herrmann, C. scArchon: a scalable benchmarking framework for assessing single-cell perturbation models. Genome Biol. 2026, 27, 162. doi:10.1186/s13059-026-04104-z	DOI 10.1186/s13059-026-04104-z	Peer-reviewed journal/record	Perturbation resource, benchmark, or relational/context model
49	Zimmerman, K.D.; Espeland, M.A.; Langefeld, C.D. A practical solution to pseudoreplication bias in single-cell studies. Nat. Commun. 2021, 12, 738. doi:10.1038/s41467-021-21038-1	DOI 10.1038/s41467-021-21038-1	Peer-reviewed journal/record	Perturbation resource, benchmark, or relational/context model
50	Squair, J.W.; Gautier, M.; Kathe, C.; Anderson, M.A.; James, N.D.; Hutson, T.H.; Hudelle, R.; Qaiser, T.; Matson, K.J.E.; Barraud, Q.; et al. Confronting false discoveries in single-cell differential expression. Nat. Commun. 2021, 12, 5692. doi:10.1038/s41467-021-25960-2	DOI 10.1038/s41467-021-25960-2	Peer-reviewed journal/record	Perturbation resource, benchmark, or relational/context model
51	Murphy, A.E.; Skene, N.G. A balanced measure shows superior performance of pseudobulk methods in single-cell RNA-sequencing analysis. Nat. Commun. 2022, 13, 7851. doi:10.1038/s41467-022-35519-4	DOI 10.1038/s41467-022-35519-4	Peer-reviewed journal/record	Perturbation resource, benchmark, or relational/context model
52	Kapoor, S.; Narayanan, A. Leakage and the reproducibility crisis in machine-learning-based science. Patterns 2023, 4, 100804. doi:10.1016/j.patter.2023.100804	DOI 10.1016/j.patter.2023.100804	Peer-reviewed journal/record	Perturbation resource, benchmark, or relational/context model

Ref.	Authenticated citation	Persistent identifier	Status	Function
53	Adduri, A.K.; Gautam, D.; Bevilacqua, B.; Imran, A.; Shah, R.; Naghipourfar, M.; Teyssier, N.; Ilango, R.; Nagaraj, S.; Dong, M.; et al. Predicting cellular responses to perturbation across diverse contexts with State. bioRxiv 2025 [preprint]. doi:10.1101/2025.06.26.661135	DOI 10.1101/2025.06.26.661135	Preprint	Perturbation resource, benchmark, or relational/context model
54	Alsulami, R.; Lehmann, R.; Khan, S.A.; Lagani, V.; Maillo, A.; Gomez-Cabrero, D.; Kiani, N.A.; Tegner, J. Predicting and interpreting cell-type-specific drug responses in the small-data regime using inductive priors. Nat. Mach. Intell. 2026, 8, 461-473. doi:10.1038/s42256-026-...	DOI 10.1038/s42256-026-01202-2	Peer-reviewed journal/record	Perturbation resource, benchmark, or relational/context model
55	Lorch, L.; Zhang, J.; Bunne, C.; Krause, A.; Schölkopf, B.; Uhler, C. Latent causal diffusions for single-cell perturbation modeling. arXiv 2026 [preprint], arXiv:2601.15341.	arXiv:2601.15341.	Preprint	Learned or biologically specified dynamical perturbation precedent
56	Lin, Z.; Chang, S.; Zweig, A.; Kang, M.; Theis, F.J.; Azizi, E.; Knowles, D.A. Interpretable neural ODEs for gene regulatory network discovery under perturbations. In Proceedings of the 43rd International Conference on Machine Learning (ICML 2026), Seoul, Republic of Korea, 20...	arXiv:2501.02409.	Preprint	Learned or biologically specified dynamical perturbation precedent
57	Yi, Z.; Ma, N.; Ao, Y. LazyNet: interpretable ODE modeling of sparse CRISPR single-cell screens reveals new biological insights. Biology 2026, 15, 62. doi:10.3390/biology15010062	DOI 10.3390/biology15010062	Peer-reviewed journal/record	Learned or biologically specified dynamical perturbation precedent
58	Gallagher-Syed, A.; Wenteler, A.; Martinez, S.A.; Slabaugh, G. CLAMP: A Mechanistic Probe of Regulatory Structure in Foundation Models under Single-Cell Perturbations. ICML 2026 Workshop on Generative and Agentic AI for Biology (GenBio), Spotlight, non-archival. OpenReview forum C0DTxoMuYO. https://openreview.net/forum?id=C0DTxoMuYO	OpenReview C0DTxoMuYO	Non-archival workshop Spotlight	Learned dynamical perturbation precedent and mechanistic probe of foundation-model regulatory structure
59	Gebru, T.; Morgenstern, J.; Vecchione, B.; Vaughan, J.W.; Wallach, H.; Daume, H., III; Crawford, K. Datasheets for datasets. Commun. ACM 2021, 64, 86-92. doi:10.1145/3458723	DOI 10.1145/3458723	Peer-reviewed journal/record	Dataset provenance and documentation
60	Yuan, B.; Shen, C.; Luna, A.; Korkut, A.; Marks, D.S.; Ingraham, J.; Sander, C. CellBox: interpretable machine learning for perturbation biology with application to the design of cancer combination therapy. Cell Syst. 2021, 12, 128-140. doi:10.1016/j.cels.2020.11.013	DOI 10.1016/j.cels.2020.11.013	Peer-reviewed journal/record	Learned or biologically specified dynamical perturbation precedent
61	Theodoris, C.V.; Xiao, L.; Chopra, A.; Chaffin, M.D.; Al Sayed, Z.R.; Hill, M.C.; Mantineo, H.; Brydon, E.M.; Zeng, Z.; Liu, X.S.; Ellinor, P.T. Transfer learning enables predictions in network biology. Nature 2023, 618, 616-624. doi:10.1038/s41586-023-06139-9	DOI 10.1038/s41586-023-06139-9	Peer-reviewed journal/record	Foundation model and leakage/transfer evidence
62	Cui, H.; Wang, C.; Maan, H.; Pang, K.; Luo, F.; Duan, N.; Wang, B. scGPT: toward building a foundation model for single-cell multi-omics using generative AI. Nat. Methods 2024, 21, 1470-1480. doi:10.1038/s41592-024-02201-0	DOI 10.1038/s41592-024-02201-0	Peer-reviewed journal/record	Foundation model and leakage/transfer evidence
63	Hao, M.; Gong, J.; Zeng, X.; Liu, C.; Guo, Y.; Cheng, X.; Wang, T.; Ma, J.; Zhang, X.; Song, L. Large-scale foundation model on single-cell transcriptomics. Nat. Methods 2024, 21, 1481-1491. doi:10.1038/s41592-024-02305-7	DOI 10.1038/s41592-024-02305-7	Peer-reviewed journal/record	Foundation model and leakage/transfer evidence
64	Rosen, Y.; Roohani, Y.; Agrawal, A.; Samotorcan, L.; Tabula Sapiens Consortium; Quake, S.R.; Leskovec, J. Universal cell embedding provides a foundation model for cell biology. Nature 2026, 656, 183-191. doi:10.1038/s41586-026-10689-z	DOI 10.1038/s41586-026-10689-z	Peer-reviewed journal/record	Foundation model and leakage/transfer evidence
65	Kedzierska, K.Z.; Crawford, L.; Amini, A.P.; Lu, A.X. Zero-shot evaluation reveals limitations of single-cell foundation models. Genome Biol. 2025, 26, 101. doi:10.1186/s13059-025-03574-x	DOI 10.1186/s13059-025-03574-x	Peer-reviewed journal/record	Foundation model and leakage/transfer evidence
66	Fröhlich, F.; Weindl, D.; Schälte, Y.; Pathirana, D.; Paszkowski, Ł.; Lines, G.T.; Stapor, P.; Hasenauer, J. AMICI: high-performance sensitivity analysis for large ordinary differential equation models. Bioinformatics 2021, 37, 3676-3677. doi:10.1093/bioinformatics/btab227	DOI 10.1093/bioinformatics/btab227	Peer-reviewed journal/record	Operator, solver, stochastic, optimization, or inference method

Table S4-67-88. Reference-authentication ledger, main-manuscript references 67-88.

Ref.	Authenticated citation	Persistent identifier	Status	Function

Ref.	Authenticated citation	Persistent identifier	Status	Function
67	Kim, S.; Ji, W.; Deng, S.; Ma, Y.; Rackauckas, C. Stiff neural ordinary differential equations. Chaos 2021, 31, 093122. doi:10.1063/5.0060697	DOI 10.1063/5.0060697	Peer-reviewed journal/record	Operator, solver, stochastic, optimization, or inference method
68	Bouhaddou, M.; Barrette, A.M.; Stern, A.D.; Koch, R.J.; DiStefano, M.S.; Riesel, E.A.; Santos, L.C.; Tan, A.L.; Mertz, A.E.; Birtwistle, M.R. A mechanistic pan-cancer pathway model informed by multi-omic data interprets stochastic cell fate responses to drugs and mitogens. PLo...	DOI 10.1371/journal.pcbi.1005985	Peer-reviewed journal/record	Operator, solver, stochastic, optimization, or inference method
69	Erdem, C.; Mutsuddy, A.; Bensman, E.M.; Dodd, W.B.; Saint-Antoine, M.M.; Bouhaddou, M.; Blake, R.C.; Dodd, S.L.; Robinson, B.S.; Sorger, P.K.; Birtwistle, M.R. A scalable, open-source implementation of a large-scale mechanistic model for single cell proliferation and death sig...	DOI 10.1038/s41467-022-31138-1	Peer-reviewed journal/record	Operator, solver, stochastic, optimization, or inference method
70	Gillespie, D.T. Exact stochastic simulation of coupled chemical reactions. J. Phys. Chem. 1977, 81, 2340-2361. doi:10.1021/j100540a008	DOI 10.1021/j100540a008	Peer-reviewed journal/record	Operator, solver, stochastic, optimization, or inference method
71	Gillespie, D.T. Approximate accelerated stochastic simulation of chemically reacting systems. J. Chem. Phys. 2001, 115, 1716-1733. doi:10.1063/1.1378322	DOI 10.1063/1.1378322	Peer-reviewed journal/record	Operator, solver, stochastic, optimization, or inference method
72	Munsky, B.; Khammash, M. The finite state projection algorithm for the solution of the chemical master equation. J. Chem. Phys. 2006, 124, 044104. doi:10.1063/1.2145882	DOI 10.1063/1.2145882	Peer-reviewed journal/record	Operator, solver, stochastic, optimization, or inference method
73	Elowitz, M.B.; Levine, A.J.; Siggia, E.D.; Swain, P.S. Stochastic gene expression in a single cell. Science 2002, 297, 1183-1186. doi:10.1126/science.1070919	DOI 10.1126/science.1070919	Peer-reviewed journal/record	Operator, solver, stochastic, optimization, or inference method
74	Raj, A.; van Oudenaarden, A. Nature, nurture, or chance: stochastic gene expression and its consequences. Cell 2008, 135, 216-226. doi:10.1016/j.cell.2008.09.050	DOI 10.1016/j.cell.2008.09.050	Peer-reviewed journal/record	Operator, solver, stochastic, optimization, or inference method
75	Mohamed, S.; Rosca, M.; Figurnov, M.; Mnih, A. Monte Carlo gradient estimation in machine learning. J. Mach. Learn. Res. 2020, 21, 1-62. arXiv:1906.10652.	arXiv:1906.10652.	Peer-reviewed journal/record	Operator, solver, stochastic, optimization, or inference method
76	Jang, E.; Gu, S.; Poole, B. Categorical reparameterization with Gumbel-softmax. In Proceedings of the International Conference on Learning Representations, 2017. arXiv:1611.01144.	arXiv:1611.01144.	Conference/proceedings	Operator, solver, stochastic, optimization, or inference method
77	Orth, J.D.; Thiele, I.; Palsson, B.Ø. What is flux balance analysis? Nat. Biotechnol. 2010, 28, 245-248. doi:10.1038/nbt.1614	DOI 10.1038/nbt.1614	Peer-reviewed journal/record	Operator, solver, stochastic, optimization, or inference method
78	Mahadevan, R.; Edwards, J.S.; Doyle, F.J. Dynamic flux balance analysis of diauxic growth in Escherichia coli. Biophys. J. 2002, 83, 1331-1340. doi:10.1016/S0006-3495(02)73903-9	DOI 10.1016/S0006-3495(02)73903-9	Peer-reviewed journal/record	Operator, solver, stochastic, optimization, or inference method

Ref.	Authenticated citation	Persistent identifier	Status	Function
79	Heirendt, L.; Arreckx, S.; Pfau, T.; Mendoza, S.N.; Richelle, A.; Heinken, A.; Haraldsdóttir, H.S.; Wachowiak, J.; Keating, S.M.; Vlasov, V.; et al. Creation and analysis of biochemical constraint-based models using the COBRA Toolbox v.3.0. Nat. Protoc. 2019, 14, 639-702. doi:...	DOI 10.1038/s41596-018-0098-2	Peer-reviewed journal/record	Operator, solver, stochastic, optimization, or inference method
80	O'Brien, E.J.; Lerman, J.A.; Chang, R.L.; Hyduke, D.R.; Palsson, B.Ø. Genome-scale models of metabolism and gene expression extend and refine growth phenotype prediction. Mol. Syst. Biol. 2013, 9, 693. doi:10.1038/msb.2013.52	DOI 10.1038/msb.2013.52	Peer-reviewed journal/record	Operator, solver, stochastic, optimization, or inference method
81	Amos, B.; Kolter, J.Z. OptNet: differentiable optimization as a layer in neural networks. In Proceedings of the 34th International Conference on Machine Learning, Sydney, Australia, 6-11 August 2017; PMLR 70, pp. 136-145. arXiv:1703.00443.	arXiv:1703.00443.	Conference/proceedings	Operator, solver, stochastic, optimization, or inference method
82	Agrawal, A.; Amos, B.; Barratt, S.; Boyd, S.; Diamond, S.; Kolter, J.Z. Differentiable convex optimization layers. Adv. Neural Inf. Process. Syst. 2019, 32, 9562-9574. arXiv:1910.12430.	arXiv:1910.12430.	Peer-reviewed journal/record	Operator, solver, stochastic, optimization, or inference method
83	Lopez, C.F.; Muhlich, J.L.; Bachman, J.A.; Sorger, P.K. Programming biological models in Python using PySB. Mol. Syst. Biol. 2013, 9, 646. doi:10.1038/msb.2013.1	DOI 10.1038/msb.2013.1	Peer-reviewed journal/record	Operator, solver, stochastic, optimization, or inference method
84	Kauffman, S.A. Metabolic stability and epigenesis in randomly constructed genetic nets. J. Theor. Biol. 1969, 22, 437-467. doi:10.1016/0022-5193(69)90015-0	DOI 10.1016/0022-5193(69)90015-0	Peer-reviewed journal/record	Operator, solver, stochastic, optimization, or inference method
85	Stoll, G.; Caron, B.; Viara, E.; Dugourd, A.; Zinovyev, A.; Naldi, A.; Kroemer, G.; Barillot, E.; Calzone, L. MaBoSS 2.0: an environment for stochastic Boolean modeling. Bioinformatics 2017, 33, 2226-2228. doi:10.1093/bioinformatics/btx123	DOI 10.1093/bioinformatics/btx123	Peer-reviewed journal/record	Operator, solver, stochastic, optimization, or inference method
86	Terfve, C.; Cokelaer, T.; Henriques, D.; MacNamara, A.; Goncalves, E.; Morris, M.K.; van Iersel, M.; Lauffenburger, D.A.; Saez-Rodriguez, J. CellNOptR: a flexible toolkit to train protein signaling networks to data using multiple logic formalisms. BMC Syst. Biol. 2012, 6, 133....	DOI 10.1186/1752-0509-6-133	Peer-reviewed journal/record	Operator, solver, stochastic, optimization, or inference method
87	Cowan, A.E.; Moraru, I.I.; Schaff, J.C.; Slepchenko, B.M.; Loew, L.M. Spatial modeling of cell signaling networks. Methods Cell Biol. 2012, 110, 195-221. doi:10.1016/B978-0-12-388403-9.00008-4	DOI 10.1016/B978-0-12-388403-9.00008-4	Peer-reviewed journal/record	Operator, solver, stochastic, optimization, or inference method
88	Ghaffarizadeh, A.; Heiland, R.; Friedman, S.H.; Mumenthaler, S.M.; Macklin, P. PhysiCell: an open source physics-based cell simulator for 3-D multicellular systems. PLoS Comput. Biol. 2018, 14, e1005991. doi:10.1371/journal.pcbi.1005991	DOI 10.1371/journal.pcbi.1005991	Peer-reviewed journal/record	Operator, solver, stochastic, optimization, or inference method

Table S4-89-111. Reference-authentication ledger, main-manuscript references 89-111.

Ref.	Authenticated citation	Persistent identifier	Status	Function
89	Swat, M.H.; Thomas, G.L.; Belmonte, J.M.; Shirinifard, A.; Hmeljak, D.; Glazier, J.A. Multi-scale modeling of tissues using CompuCell3D. Methods Cell Biol. 2012, 110, 325-366. doi:10.1016/B978-0-12-388403-9.00013-8	DOI 10.1016/B978-0-12-388403-9.00013-8	Peer-reviewed journal/record	Operator, solver, stochastic, optimization, or inference method
90	Li, Z.; Kovachki, N.; Azizzadenesheli, K.; Liu, B.; Bhattacharya, K.; Stuart, A.; Anandkumar, A. Fourier neural operator for parametric partial differential equations. In Proceedings of the International Conference on Learning Representations, 2021. arXiv:2010.08895.	arXiv:2010.08895.	Conference/proceedings	Operator, solver, stochastic, optimization, or inference method
91	Lu, L.; Jin, P.; Pang, G.; Zhang, Z.; Karniadakis, G.E. Learning nonlinear operators via DeepONet based on the universal approximation theorem of operators. Nat. Mach. Intell. 2021, 3, 218-229. doi:10.1038/s42256-021-00302-5	DOI 10.1038/s42256-021-00302-5	Peer-reviewed journal/record	Operator, solver, stochastic, optimization, or inference method
92	Cranmer, K.; Brehmer, J.; Louppe, G. The frontier of simulation-based inference. Proc. Natl. Acad. Sci. USA 2020, 117, 30055-30062. doi:10.1073/pnas.1912789117	DOI 10.1073/pnas.1912789117	Peer-reviewed journal/record	Operator, solver, stochastic, optimization, or inference method
93	Tejero-Cantero, A.; Boelts, J.; Deistler, M.; Lueckmann, J.-M.; Durkan, C.; Gonçalves, P.J.; Greenberg, D.S.; Macke, J.H. sbi: a toolkit for simulation-based inference. J. Open Source Softw. 2020, 5, 2505. doi:10.21105/joss.02505	DOI 10.21105/joss.02505	Peer-reviewed journal/record	Operator, solver, stochastic, optimization, or inference method
94	Rackauckas, C.; Ma, Y.; Martensen, J.; Warner, C.; Zubov, K.; Supekar, R.; Skinner, D.; Ramadhan, A.; Edelman, A. Universal differential equations for scientific machine learning. arXiv 2020 [preprint], arXiv:2001.04385.	arXiv:2001.04385.	Preprint	Operator, solver, stochastic, optimization, or inference method
95	Chen, R.T.Q.; Rubanova, Y.; Bettencourt, J.; Duvenaud, D. Neural ordinary differential equations. Adv. Neural Inf. Process. Syst. 2018, 31, 6571-6583. arXiv:1806.07366.	arXiv:1806.07366.	Peer-reviewed journal/record	Operator, solver, stochastic, optimization, or inference method
96	Raissi, M.; Perdikaris, P.; Karniadakis, G.E. Physics-informed neural networks: a deep learning framework for solving forward and inverse problems involving nonlinear partial differential equations. J. Comput. Phys. 2019, 378, 686-707. doi:10.1016/j.jcp.2018.10.045	DOI 10.1016/j.jcp.2018.10.045	Peer-reviewed journal/record	Operator, solver, stochastic, optimization, or inference method
97	Lopez, R.; Regier, J.; Cole, M.B.; Jordan, M.I.; Yosef, N. Deep generative modeling for single-cell transcriptomics. Nat. Methods 2018, 15, 1053-1058. doi:10.1038/s41592-018-0229-2	DOI 10.1038/s41592-018-0229-2	Peer-reviewed journal/record	Operator, solver, stochastic, optimization, or inference method
98	Kennedy, M.C.; O'Hagan, A. Bayesian calibration of computer models. J. R. Stat. Soc. Ser. B 2001, 63, 425-464. doi:10.1111/1467-9868.00294	DOI 10.1111/1467-9868.00294	Peer-reviewed journal/record	Discrepancy, uncertainty, scoring, or compute methodology

Ref.	Authenticated citation	Persistent identifier	Status	Function
99	Brynjarsdóttir, J.; O'Hagan, A. Learning about physical parameters: the importance of model discrepancy. Inverse Probl. 2014, 30, 114007. doi:10.1088/0266-5611/30/11/114007	DOI 10.1088/0266-5611/30/11/114007	Peer-reviewed journal/record	Discrepancy, uncertainty, scoring, or compute methodology
100	Lakshminarayanan, B.; Pritzel, A.; Blundell, C. Simple and scalable predictive uncertainty estimation using deep ensembles. Adv. Neural Inf. Process. Syst. 2017, 30, 6402-6413. arXiv:1612.01474.	arXiv:1612.01474.	Peer-reviewed journal/record	Discrepancy, uncertainty, scoring, or compute methodology
101	Gneiting, T.; Raftery, A.E. Strictly proper scoring rules, prediction, and estimation. J. Am. Stat. Assoc. 2007, 102, 359-378. doi:10.1198/016214506000001437	DOI 10.1198/016214506000001437	Peer-reviewed journal/record	Discrepancy, uncertainty, scoring, or compute methodology
102	Angelopoulos, A.N.; Bates, S. A gentle introduction to conformal prediction and distribution-free uncertainty quantification. arXiv 2021 [preprint], arXiv:2107.07511.	arXiv:2107.07511.	Preprint	Discrepancy, uncertainty, scoring, or compute methodology
103	Schwartz, R.; Dodge, J.; Smith, N.A.; Etzioni, O. Green AI. Commun. ACM 2020, 63, 54-63. doi:10.1145/3381831	DOI 10.1145/3381831	Peer-reviewed journal/record	Discrepancy, uncertainty, scoring, or compute methodology
104	Walter, P.; Ron, D. The unfolded protein response: from stress pathway to homeostatic regulation. Science 2011, 334, 1081-1086. doi:10.1126/science.1209038	DOI 10.1126/science.1209038	Peer-reviewed journal/record	UPR biology and dynamical modeling
105	Hetz, C.; Zhang, K.; Kaufman, R.J. Mechanisms, regulation and functions of the unfolded protein response. Nat. Rev. Mol. Cell Biol. 2020, 21, 421-438. doi:10.1038/s41580-020-0250-z	DOI 10.1038/s41580-020-0250-z	Peer-reviewed journal/record	UPR biology and dynamical modeling
106	Erguler, K.; Pieri, M.; Deltas, C. A mathematical model of the unfolded protein stress response reveals the decision mechanism for recovery, adaptation and apoptosis. BMC Syst. Biol. 2013, 7, 16. doi:10.1186/1752-0509-7-16	DOI 10.1186/1752-0509-7-16	Peer-reviewed journal/record	UPR biology and dynamical modeling
107	Diedrichs, D.R.; Gomez, J.A.; Huang, C.S.; Rutkowski, D.T.; Curtu, R. A data-entrained computational model for testing the regulatory logic of the vertebrate unfolded protein response. Mol. Biol. Cell 2018, 29, 1502-1517. doi:10.1091/mbc.E17-09-0565	DOI 10.1091/mbc.E17-09-0565	Peer-reviewed journal/record	UPR biology and dynamical modeling
108	Yang, H.; Niemeijer, M.; van de Water, B.; Beltman, J.B. ATF6 is a critical determinant of CHOP dynamics during the unfolded protein response. iScience 2020, 23, 100860. doi:10.1016/j.isci.2020.100860	DOI 10.1016/j.isci.2020.100860	Peer-reviewed journal/record	UPR biology and dynamical modeling
109	Wilkinson, M.D.; Dumontier, M.; Aalbersberg, I.J.; Appleton, G.; Axton, M.; Baak, A.; Blomberg, N.; Boiten, J.-W.; da Silva Santos, L.B.; Bourne, P.E.; et al. The FAIR Guiding Principles for scientific data management and stewardship. Sci. Data 2016, 3, 160018. doi:10.1038/sda...	DOI 10.1038/sdata.2016.18	Peer-reviewed journal/record	Provenance, FAIR metadata, or atlas context
110	Regev, A.; Teichmann, S.A.; Lander, E.S.; Amit, I.; Benoist, C.; Birney, E.; Bodenmiller, B.; Campbell, P.; Carninci, P.; Clatworthy, M.; et al. The Human Cell Atlas. eLife 2017, 6, e27041. doi:10.7554/eLife.27041	DOI 10.7554/eLife.27041	Peer-reviewed journal/record	Provenance, FAIR metadata, or atlas context

Ref.	Authenticated citation	Persistent identifier	Status	Function
111	Ye, J.; Rawson, R.B.; Komuro, R.; Chen, X.; Davé, U.P.; Prywes, R.; Brown, M.S.; Goldstein, J.L. ER stress induces cleavage of membrane-bound ATF6 by the same proteases that process SREBPs. Mol. Cell 2000, 6, 1355-1364. doi:10.1016/S1097-2765(00)00133-7	DOI 10.1016/S1097-2765(00)00133-7	Peer-reviewed journal/record	ATF6 cleavage and prespecified MBTPS1/MBTPS2 SREBP off-pathway risk

S5. Unfolded-Protein-Response Operator and Capacity Specification

The proposed operator is intentionally reduced to ten scaled states. PERK, IRE1, ATF6, and phosphorylated eIF2α are represented as scaled active abundance or activity rather than immutable fractions so that target knockdown can reduce the attainable pool without being misrepresented as a change in an intrinsic activation rate. Direct pathway measurements are used at Stage 1 to determine which kinetic quantities remain interpretable before the transcriptomic prediction stage.

Table S5. UPR state vector and preferred direct observations.

Symbol	Biological quantity	Preferred direct observation	Role in the reduced operator
U	Unfolded or misfolded protein load in the endoplasmic reticulum	Stress reporter, aggregation-sensitive probe, or calibrated surrogate	Drives sensor activation and consumes folding capacity
P	Scaled active PERK abundance/activity	PERK phosphorylation or kinase reporter	Initiates eIF2α phosphorylation and translational attenuation
E	Scaled phosphorylated eIF2α abundance/activity	Phospho-eIF2α measurement	Reduces global translation and promotes selective ATF4 translation
A	ATF4 activity	ATF4 protein or transcriptional reporter	Controls integrated-stress-response genes and contributes to CHOP induction
I	Scaled active IRE1 abundance/activity	IRE1 phosphorylation or RNase activity	Controls XBP1 splicing and adaptive output
X	Spliced XBP1 activity	XBP1s RNA or protein	Induces folding and degradation programs
F	Nuclear cleaved ATF6 abundance/activity	Cleaved ATF6 or nuclear reporter	Induces chaperone genes and modifies CHOP dynamics
H	Effective chaperone and folding capacity	BiP or GRP78 and selected folding-capacity readouts	Clears unfolded load and mediates negative feedback
C	CHOP-associated terminal-stress signal	CHOP RNA, protein, or reporter	Stress-severity and cell-fate endpoint
G	GADD34-directed eIF2α phosphatase activity	GADD34 RNA or protein and phospho-eIF2α recovery	Delayed negative feedback that restores translation

Table S6. Capacity ledger for the Stage 2 configuration.

Component	Fixed before fitting	Trainable at Stage 2	Count formula
Pretrained encoder	Checkpoint, corpus, tokenization	0 (frozen)	Identical across all arms; capacity shared, not compared
Initial-state model q_η	Architecture, two hidden layers of width 32, input G + 1	5,620	(121×32 + 32) + (32×32 + 32) + (32×20 + 20)
Context model (B, b_c)	Disabled at Stages 1 and 2	0	Activated only at Stage 3b with at least four training contexts
Mechanistic operator θ	37 kinetic parameters and thresholds; signs and bounds declared	12 at most	Prospective cap; sensitivity analyses at 8 and 16
Chemical intervention operator θI	Chemical target and action; exposure-function forms frozen	4	2 stressors × (maximal effect, half-maximal concentration)

Component	Fixed before fitting	Trainable at Stage 2	Count formula
Genetic action inputs aj	Target-to-process mapping plus assay-derived e_j and ρ_j distributions frozen before Stage 2	0	16 externally specified action distributions; uncertainty propagated but never fitted to locked outcomes
Observation map	Module membership, endpoint gene weights, sign of each loading	360	3G: intercept, one nonzero module loading, dispersion per gene
Discrepancy δ	Rank 3, regularization operator R, latent δ_{rt} marginalized	369	3G loadings + 3 diagonal scales + 3×2 time offsets
Total	Trainable structure plus frozen 120-gene panel and genetic action inputs	6,365	5,620 + 12 + 4 + 360 + 369; plus 16 fixed genetic action distributions

The genetic intervention contract uses an independently measured guide knockdown efficiency e_j and an independently characterized target-to-process coefficient ρ_j, with a_j = 1 − ρ_j e_j. Neither quantity is treated as known without error. The e_j distribution is estimated from the targeted-RNA assay and technical replicates, while the ρ_j distribution is obtained only from the proximal or functional assay assigned to that action. These assay-derived distributions and the target-to-process mapping are frozen before the Stage 2 partition is generated and are propagated through the primary predictive distribution; Stage 2 outcomes never update them. The Stage 1 assay plan must also freeze a target-engagement precision criterion. A candidate whose external action uncertainty does not meet that criterion is not eligible for confirmatory testing.

Sensor and chemical functions: h_j(U,H) = (U/H)^2 / [K_j^2 + (U/H)^2], for j in {P,I,F}. Tunicamycin acts through q_load(u,d,t) = E_TM d/(EC50_TM + d) 1[t >= 0]. Thapsigargin is time gated: g_Ca(u,d,t) = 1 for t < 0 and max(0.05, 1 - E_TG d/(EC50_TG + d)) for t >= 0. The four chemical intervention parameters are E_TM, EC50_TM, E_TG, and EC50_TG.

Reduced ODE system: dU/dt = q_load + k_syn - k_fold g_Ca H U/(K_U + U) - a_U k_U U; dP/dt = k_P h_P(U,H)(a_P-P)-d_P P; dE/dt = k_E P(a_E-E)-(d_E+k_EG G)E; dA/dt = a_A k_A E/(K_A+E)-d_A A; dI/dt = k_I h_I(U,H)(a_I-I)-d_I I; dX/dt = a_X k_X I-d_X X; dF/dt = k_F h_F(U,H)(a_F-F)-d_F F; dH/dt = a_H(k_H0 + k_HX X + k_HF F + k_HA A) - k_HU H U/(K_H+U)-d_H H; dC/dt = a_C[k_CA A + k_CF F + k_CU U/(K_C+U)]-d_C C; dG/dt = a_G[k_G0+k_GA A+k_GC C]-d_G G. The primary convention is that an availability multiplier on a produced species scales its complete production/input block unless a different action is declared prospectively. For H, two sensitivity equations are frozen. Basal-only sensitivity: dH/dt = a_H k_H0 + k_HX X + k_HF F + k_HA A - k_HU H U/(K_H+U)-d_H H. Folding-efficiency sensitivity: dU/dt = q_load + k_syn - a_H k_fold g_Ca H U/(K_U+U) - a_U k_U U, while dH/dt = k_H0 + k_HX X + k_HF F + k_HA A - k_HU H U/(K_H+U)-d_H H. These alternatives test whether chaperone knockdown acts primarily through total effective chaperone production, basal input only, or folding efficiency per unit H.

Table S7. Prespecified target-to-operator mapping and partition roles.

The held-out set is intentionally action-diverse: HSP90B1, SEL1L, MBTPS2, XBP1, and PPP1R15A/GADD34 represent a_H, a_U, a_F, a_X, and a_G, respectively. HSP90B1, SEL1L, and MBTPS2 are the primary known-action target-generalization stratum because their action classes are represented in training. XBP1 and PPP1R15A are secondary new-action extrapolations because a_X and a_G are not exercised during training; those tests therefore challenge the declared intervention functional form as well as the proposed topology. A pooled five-action summary is secondary and is not used to attribute success to either stratum. Validation uses EIF2S1/a_E, PDIA4/a_H, and MBTPS1/a_F; DNAJB11 is assigned to training. Even with three validation action classes, validation selection remains small-sample, so action-stratified selection stability is reported. MBTPS1 and MBTPS2 are declared off-pathway risks because the same Site-1/Site-2 proteases process SREBPs as well as ATF6; EIF2S1 knockdown can perturb global translation outside the reduced UPR representation. These effects are expected to appear as model discrepancy or failed direct-readout checks rather than being absorbed into kinetic parameters [104,105,111].

Interference target	Declared operator action	State/process affected	Partition and independent eligibility evidence
EIF2AK3 (PERK)	Attainable PERK pool aP	P	Training; Stage 1 calibration; proximal PERK assay

Interference target	Declared operator action	State/process affected	Partition and independent eligibility evidence
ERN1 (IRE1)	Attainable IRE1 pool aI	I	Training; Stage 1 calibration; IRE1/XBP1s assay
ATF6	Attainable ATF6 pool aF	F	Training; Stage 1 calibration; cleaved-ATF6 assay
EIF2S1	Available eIF2α substrate aE	E	Validation; target-engagement assay; declared global-translation risk
ATF4	ATF4 production multiplier aA	A	Training; ATF4 protein/reporter check
DDIT3 (CHOP)	CHOP production multiplier aC	C	Training; CHOP protein/reporter check
HSPA5 (BiP)	Effective-chaperone production multiplier aH on the full H input block	H	Training; prechallenge folding/chaperone assay defines ρj distribution
EDEM1	ERAD-clearance multiplier aU on kU	U	Training; prechallenge ERAD assay defines ρj distribution
XBP1	XBP1s production multiplier aX	X	Held-out test; new action class aX; secondary extrapolation; XBP1-splicing assay
PPP1R15A (GADD34)	Inducible phosphatase-production multiplier aG	G	Held-out test; new action class aG; secondary extrapolation; GADD34/phospho-eIF2α recovery assay
MBTPS1	ATF6 cleavage/availability multiplier aF	F	Validation; ATF6-cleavage assay; declared SREBP-processing risk
HSP90B1	Effective-chaperone production multiplier aH on the full H input block	H	Held-out test; known action class aH, new gene; independent functional assay
PDIA4	Effective-chaperone production multiplier aH on the full H input block	H	Validation; independent functional assay
DNAJB11	Effective-chaperone production multiplier aH on the full H input block	H	Training; independent functional assay defines ρ_j distribution
SEL1L	ERAD-clearance multiplier aU on kU	U	Held-out test; known action class aU, new gene; independent ERAD assay
MBTPS2	ATF6 cleavage/availability multiplier aF	F	Held-out test; known action class aF, new gene; declared SREBP-processing risk

S6. Sampling, Stage 1/1b, and Data Provenance

Stage 1 is an identification and intervention-semantics experiment. It uses only interventions assigned to Stage 2 training. The direct-readout series measures phospho-eIF2α, ATF4, XBP1s, cleaved ATF6, BiP/HSPA5, CHOP, and GADD34. A nominal eight-point schedule is baseline plus 0.5, 1, 2, 4, 6, 12, and 24 hours; the exact laboratory schedule is frozen before data collection. The two Stage 2 endpoint times are selected by the prespecified rule in the main manuscript and frozen before Stage 2. Stage 1b then uses separate pilot cultures and passages, not the six Stage 2

confirmatory cultures, to collect training-intervention RNA at the two selected times for variance, covariance, scaling, and power estimation. Stage 1b wells are not reused as locked Stage 2 confirmatory outcomes.

Sampling element	Planned observations	Purpose
Baseline	Untreated and post-CRISPRi/pre-stressor RNA; direct pathway state; culture, passage, assay metadata	Initial-state distribution and prechallenge target engagement
0.5, 1, 2 h	Phospho-eIF2α, ATF4 onset, XBP1s, cleaved ATF6, proximal target assays	Branch activation, action coefficients, early kinetics
4, 6 h	ATF4, XBP1s, cleaved ATF6, BiP, GADD34, CHOP	Adaptive branch dynamics and candidate early endpoint
12, 24 h	ATF4, CHOP, GADD34, BiP, viability/terminal-stress readouts	Late adaptation/terminal-stress endpoint selection
Stage 1b RNA variance pilot	Training interventions only; transcriptomic panel at the two selected endpoint times in pilot cultures/passages separate from the six Stage 2 confirmatory cultures	Estimate module-time covariance, residual well variance, intervention-by-culture response variance, predictive noise floor, and prospective power; pilot wells are not pooled into the locked Stage 2 confirmatory outcome set
Stage 3 reference panel	At least 20 untreated cultures with compatible library chemistry/preprocessing; no intervention outcomes used in model fitting	External calibration of context-distance and abstention threshold; if unavailable, no distance-based abstention claim is made

Stage 1 and the Stage 2 confirmatory test set are disjoint. No intervention that contributes orthogonal response data for model identification is eligible for the Stage 2 held-out target set. The chemical dose axis is analyzed separately from the genetic target-generalization axis and is not averaged into the primary confirmatory result.

S7. Statistical Analysis, Falsification, and Comparator Contract

For the culture-averaged control-subtracted module-time endpoint, RMSE_floor^2 = $[\sigma_{uc}^2 + \sigma_w^2(1 + 1/m)] / n\_cult$, where σ_{uc}^2 is the intervention-by-culture random-effect variance from the prespecified training mixed model after fixed design covariates, σ_w^2 is residual well-level variance after standardization, m is the number of matched control wells contributing to the within-culture control mean, and n_cult is the number of independent cultures averaged in the intervention-level score. The multivariate implementation uses the corresponding module-time covariance matrix before division by C. These are design-based training/Stage 1b variance components and are not redefined for each predictor. S(M) = 1 therefore means performance at the prediction floor permitted by the frozen measurement and replication design, not complete recovery of biological signal. A predictor may achieve S(M) greater than one when it explains variation treated as random in the design model. Because the floor depends on n_cult, the normalized scale, culture count, and MPG threshold are frozen together.

Mechanistic predictive gain (MPG) is the difference between normalized predictive scores of the biologically specified hybrid and the validation-selected strongest operator-isolation comparator. The primary isolation set contains late fusion, the capacity-matched static unstructured network, the capacity-matched learned-dynamical operator, and a validation-frozen hard-rewiring subset. The learned-dynamical operator receives the same frozen target-to-action mapping, the same assay-derived a_j distributions, the same state dimension, and the same intervention functions; only the internal state-equation connectivity and coefficients are learned. A prespecified sparsity/parameter-tying rule limits its free state-equation coefficients to exactly the same operator-block count as the biologically specified model after Stage 1. The rewiring subset is selected from training/validation data only: after fitting admissible rewires, the top 5 percent by validation score, with a minimum of ten, is frozen before test access; locked-test predictions are averaged across that frozen subset. No test-set maximum or percentile selection is permitted.

Table S8. Stage-gated development and quantitative decision rules.

Stage	Data and task	Required analyses	Quantitative gate

Stage	Data and task	Required analyses	Quantitative gate
0. Synthetic recovery	Simulated trajectories, replicate counts, batch effects, one omitted reaction	Gradient verification, parameter and state coverage, posterior predictive checks, misspecification detection	Maximum relative gradient error below 0.001; 90 percent credible-interval coverage between 0.85 and 0.95; omitted reaction detected with Bayes factor above 10 or discrepancy variance share above 0.20
1. Direct readouts + variance pilot	Eight-time direct pathway series plus training-only RNA variance pilot in separate pilot cultures at the two selected endpoint times	Identifiability, posterior calibration, intervention-action validation, endpoint-time selection, module-time covariance and variance-component estimation	Identifiability criteria met; at most twelve free kinetic quantities; two endpoint times frozen; variance-components noise floor and 80% power design estimable before Stage 2
2a. Restricted transcriptomics, predictive gate	Five held-out genetic targets total: three known-action targets form the primary test and two new-action targets form a secondary analysis; six cultures, two frozen times, one context	Primary prediction on the three known-action targets against the fixed general comparator; separate secondary new-action and pooled five-target reports; likelihood calibration; observation-map ablation	Normalized predictive advantage over the fixed comparator with paired 95 percent lower bound above zero; predictive-interval coverage between 0.85 and 0.95
2b. Mechanistic-attribution gate	Primary known-action partition after Stage 2a passes; new-action stratum analyzed secondarily	Primary confirmatory MPG across HSP90B1, SEL1L, and MBTPS2; simultaneous comparison with four isolation controls; discrepancy analysis; separate new-action analysis	Primary confirmatory gate: MPG > 0.25 with paired lower 95% bound > 0; simultaneous interval excludes the negligible region for late fusion, hard rewirings, capacity-matched static network, and matched learned-dynamical operator; discrepancy share < 0.30
3. Second-context external validation	One additional cell line or donor, model frozen, context terms inactive	Baseline-conditioned external prediction, distance-stress analysis, direct-readout checks	Normalized gain above 0.15 with coverage in band, initial states inside the calibrated domain, no solver failure or out-of-bound trajectory
4. Prospective locked batch	New independently generated cultures after freezing	Frozen code and weights, attempt ledger, publicly preregistered analysis, prospective intervals	Prespecified primary interval excludes the negligible region; coverage criterion met; no post hoc model change

Table S9. Locked evaluation contract.

Claim	Partition and unit	Primary comparison and endpoint	Required supporting evidence	Refutation
Held-out intervention prediction	Three primary known-action held-out targets plus two secondary new-action targets; six independent Stage 2 cultures; action class is the pairing unit	Primary: full hybrid versus validation-selected general predictor on HSP90B1, SEL1L, and MBTPS2; secondary: XBP1, PPP1R15A, and pooled five-target summaries	Exploratory metric panel, variance-components noise floor, interval calibration, attempt ledger	Predictive advantage has paired lower 95% bound ≤ 0 or calibration gate fails
Mechanistic interpretation	Same Stage 2 partition with Stage 1 orthogonal pathway measurements and prespecified target-action evidence	Profile-likelihood/posterior identifiability, parameter admissibility, bounded discrepancy	State coverage, posterior-predictive checks, intervention-action validation	Any free kinetic quantity fails the Stage 1 criteria, discrepancy share exceeds 0.30, or interpretation is prior-sensitive

Claim	Partition and unit	Primary comparison and endpoint	Required supporting evidence	Refutation
Mechanistic predictive gain	Paired scores on the three primary known-action classes; new-action MPG reported separately as secondary	Hybrid versus late fusion, validation-frozen hard rewirings, capacity-matched static network, and matched learned-dynamical operator	Paired design-unit interval, matched compute, multiple-comparisons-with-the-best interval	MPG < 0.25 or the simultaneous interval fails to exclude the negligible region for any isolation control
External case validation	One additional cell line or donor; model frozen; context terms inactive; initial state updated from baseline only	Frozen hybrid versus the fixed general predictor and late fusion	Externally calibrated context-distance stress curve, direct-readout checks, predictive coverage	Gain < 0.15, coverage failure, baseline state outside calibrated domain, solver/trajectory failure, or failed direct-readout/posterior-predictive check
Prospective reproducibility	New locked batch or site after model and analysis freezing	Frozen model and analysis versus the prespecified comparator	Sample and test manifests, prospective coverage, no post hoc model change	Prespecified predictive or calibration criterion is not met

Power is prospectively calculated for the Stage 2b MPG design effect of 0.25. A transparent first-pass action-count approximation is n_action approximately [(z_(1-α*) + z_0.80) σΔ / 0.25]^2, with α* = 0.05/4 as a conservative planning value for the four isolation comparisons. Numerically this is approximately n_action = 152 σΔ^2. Five independent action classes would therefore require σΔ no greater than about 0.18, while the three-class primary known-action analysis requires σΔ no greater than about 0.14. This closed form is a lower-bound planning screen, not the final power estimate: simulation or parametric bootstrap under the full module-time covariance, culture hierarchy, culture averaging, Stage 2a-to-2b gatekeeping, and four-comparator conjunction can be more demanding, not less. If 80 percent power cannot be achieved for the planned three-class primary known-action analysis, the known-action target pool or culture count is increased before Stage 2 is frozen; the 0.25 effect threshold is not lowered. Sensitivity analyses at MPG values of 0.15 and 0.35 are reported regardless of the primary result.

Control	Question answered	Interpretation if performance is unchanged
Operator alone	Does the curated operator predict without a learned representation?	The learned component may be unnecessary
Late fusion	Does gradient coupling add value beyond the same simulated features?	Tighter coupling is not justified
Capacity-matched unstructured network	Is the gain due to capacity rather than structure?	No evidence that mechanistic restriction adds information
Rewiring ensemble	Does the declared biological topology matter?	Graph architecture or regularization, rather than the declared biological topology, drives the result
Sign and direction scrambling	Are activating, inhibitory, and directional relations informative?	Edge semantics contribute nothing measurable
Wrong plausible pathway	Would any biological-looking prior work?	The result supports generic regularization rather than the declared mechanism
Randomized kinetic scales	Do physically plausible rates matter?	Topology alone carries the result and kinetic interpretation is unsupported
Observation-map ablation	Is the operator or the decoder producing the gain?	An unrestricted map is doing the predictive work
Discrepancy ablation and prior sensitivity	Is structural error represented rather than hidden?	Parameter interpretations are unstable or the operator is misspecified
Encoder frozen versus unfrozen	Is pretraining transfer useful under equal tuning?	Pretraining adds no value or introduces contamination and instability
Matched learned-dynamical operator	Does predeclared topology add value when a learned state equation receives the same fixed intervention mapping, action distributions, state dimension, and equal operator-block capacity?	If performance is equal or better, dynamics are supported but incremental value from the predeclared topology is not established.

S8. Solver, Gradient, Capacity, and Compute Checklist

Domain	Required frozen/reportable item
Solver	Name/version; precision; positivity handling; absolute/relative tolerances; maximum steps; event handling; stiffness diagnostics.
Gradient provenance	Forward/direct/continuous-adjoint/discrete-adjoint method; finite-difference check; maximum relative gradient error; parameters tested.

Domain	Required frozen/reportable item
Initial-state inference	q_eta architecture; conditional prior p(x0|c); Monte Carlo sample count; KL implementation; seed and convergence checks.
Discrepancy	Rank fixed at 3; 3G gene loadings; three diagonal scales; two three-vector time offsets; first-difference/ridge regularization; held-out delta marginalized from learned prior without outcome conditioning.
Rank sensitivity	Ranks 0, 1, 2, and 5 evaluated only as training/validation sensitivity analyses; no rank selection from locked test outcomes.
Capacity matching	Total trainable capacity 6,365 at G=120 under the stated architecture; operator-isolation controls share encoder, initial-state model, intervention inputs, observation map, and discrepancy and match the operator-block trainable count.
Compute budget	Accelerator-hour-equivalents; declared CPU conversion; wall time; energy where available; memory; solver calls; failed runs; seeds; tuning trials; early-stopping decisions.
Attempt ledger	Every locked-test execution, including failed or exploratory executions, with timestamp, commit/checkpoint hash, configuration, and reason.
Numerical failure rule	No model receives credit because a comparator failed numerically; unstable negative-control draws are rejected according to the prespecified admissibility rule and redrawn before locked testing.

S9. Module-Gene Panel Freeze Template

The manuscript specifies a 120-gene restricted confirmatory panel but does not present an observed or post hoc gene list. Before the Stage 2 split is opened, the panel is frozen from external UPR knowledge plus the training partition after globally excluding all 16 candidate CRISPRi target transcripts and any additional transcript predeclared as directly guide-affected. These transcripts remain available for target-engagement quality control and a sensitivity observation model but cannot contribute to the primary module score. The eligible panel is then replenished to exactly 120 genes and equal within-module endpoint weights are frozen. The table below is the required freeze record and prevents both direct-knockdown contamination and outcome-driven gene selection.

Row	Gene symbol	Module	External/training rationale	Endpoint weight	State-loading sign	Freeze note/date
1		PERK-eIF2α-ATF4 / IRE1-XBP1s-ERAD / ATF6-chaperone / CHOP-terminal stress		Equal within module	+ / -	
2		PERK-eIF2α-ATF4 / IRE1-XBP1s-ERAD / ATF6-chaperone / CHOP-terminal stress		Equal within module	+ / -	
3		PERK-eIF2α-ATF4 / IRE1-XBP1s-ERAD / ATF6-chaperone / CHOP-terminal stress		Equal within module	+ / -	
4		PERK-eIF2α-ATF4 / IRE1-XBP1s-ERAD / ATF6-chaperone / CHOP-terminal stress		Equal within module	+ / -	
5		PERK-eIF2α-ATF4 / IRE1-XBP1s-ERAD / ATF6-chaperone / CHOP-terminal stress		Equal within module	+ / -	
6		PERK-eIF2α-ATF4 / IRE1-XBP1s-ERAD / ATF6-chaperone / CHOP-terminal stress		Equal within module	+ / -	
7		PERK-eIF2α-ATF4 / IRE1-XBP1s-ERAD / ATF6-chaperone / CHOP-terminal stress		Equal within module	+ / -	
8		PERK-eIF2α-ATF4 / IRE1-XBP1s-ERAD / ATF6-chaperone / CHOP-terminal stress		Equal within module	+ / -	

Row	Gene symbol	Module	External/training rationale	Endpoint weight	State-loading sign	Freeze note/date
9		PERK-eIF2α-ATF4 / IRE1-XBP1s-ERAD / ATF6-chaperone / CHOP-terminal stress		Equal within module	+ / -	
10		PERK-eIF2α-ATF4 / IRE1-XBP1s-ERAD / ATF6-chaperone / CHOP-terminal stress		Equal within module	+ / -	
11		PERK-eIF2α-ATF4 / IRE1-XBP1s-ERAD / ATF6-chaperone / CHOP-terminal stress		Equal within module	+ / -	
12		PERK-eIF2α-ATF4 / IRE1-XBP1s-ERAD / ATF6-chaperone / CHOP-terminal stress		Equal within module	+ / -	
13		PERK-eIF2α-ATF4 / IRE1-XBP1s-ERAD / ATF6-chaperone / CHOP-terminal stress		Equal within module	+ / -	
14		PERK-eIF2α-ATF4 / IRE1-XBP1s-ERAD / ATF6-chaperone / CHOP-terminal stress		Equal within module	+ / -	
15		PERK-eIF2α-ATF4 / IRE1-XBP1s-ERAD / ATF6-chaperone / CHOP-terminal stress		Equal within module	+ / -	

The full 120-row version of this template is to be completed from eligible genes before the Stage 2 split is opened. The freeze record must separately list the excluded CRISPRi targets and any other predeclared directly guide-affected transcripts, with the reason and date of exclusion. Zero counts remain observations; no gene is removed because of a locked-test outcome. Any library-quality exclusion applies at the well level under prespecified, outcome-independent criteria. A sensitivity analysis may reinstate target transcripts only through an explicit direct-knockdown observation term whose additional coefficients and capacity are reported.

S10. Cross-Scale Interface Contract for Any Future Second Operator

No second operator or biological scale is added until the single-operator system passes its stated predictive, mechanistic, calibration, and compute gates. A future extension must complete the following interface record before implementation.

Interface item	Required declaration
Upstream state	Name, biological meaning, units/scaling, uncertainty representation, and sampling interval.
Downstream state	Name, biological meaning, units/scaling, uncertainty representation, and update interval.
Coupling direction	Forward, feedback, or bidirectional; exact variables transmitted.
Aggregation/disaggregation	Mathematical mapping between scales and conservation assumptions.
Time synchronization	Operator-splitting/synchronization scheme and step sizes.
Boundary/initial conditions	Source, measurement or inference method, and uncertainty.
Numerical error	Splitting error, solver error, tolerance study, and failure threshold.
Uncertainty propagation	How upstream uncertainty enters downstream predictions and returns through feedback.
Isolation control	Late fusion or decoupled alternative using the same upstream information without end-to-end coupling.
Falsification rule	Result that rejects the added interface or second operator without redefining the endpoint.

S11. Submission-Readiness and Prospective-Implementation Audit

Item	Status in this submission	Action before confirmatory implementation

Item	Status in this submission	Action before confirmatory implementation
Main scientific framing	Complete: operator taxonomy, learned-dynamical literature, narrowed evidence gap, UPR protocol, and falsification hierarchy synchronized.	None beyond ordinary editorial revision.
Literature search	Search rerun through 14 Aug 2026; fast-moving publication status/citation metadata rechecked 22 Aug 2026.	Rerun status check on the actual submission-freeze date.
Reference ledger	Synchronized to 111 main-manuscript references, including CLAMP/OpenReview, ICML 2026 PerturbODE metadata, and the ATF6/SREBP protease precedent used for the declared MBTPS off-pathway risk.	Confirm final publisher/proceedings metadata immediately before submission.
Experimental data	Not generated.	Generate Stage 1 and Stage 1b data only after protocol freeze and required approvals/logistics.
Synthetic recovery	Not run; Stage 0 is prospective.	Run Stage 0 before biological fitting and document any protocol amendment before Stage 2.
Public registration	Not claimed as already completed.	Deposit the Stage 2 protocol, thresholds, comparator versions, panel freeze, and analysis code in a public dated registry before confirmatory outcome access.
Gene panel	120-gene size and freeze procedure specified; candidate CRISPRi targets and any predeclared directly guide-affected transcripts are globally excluded from the primary endpoint; final eligible membership is not presented as already frozen.	Complete the eligible 120-row freeze record and separate exclusion log before Stage 2 test access; retain excluded target genes for QC/sensitivity only.
Context-distance panel	Design requires at least 20 compatible untreated cultures.	Assemble and freeze the external reference panel before Stage 3; otherwise defer distance-based abstention claims.
Stage 2 power	Closed-form action-count approximation and required variance components specified for the three-class primary known-action test; final simulation-based power result not yet available.	Use Stage 1b variance components from separate pilot cultures; enlarge known-action classes and/or cultures if 80% power at MPG = 0.25 is not achieved.
Version control	Conceptual comparator/version requirements specified.	Freeze software releases, checkpoint hashes, preprocessing, solver versions, and compute conversion rules in the public protocol.

End of Supplementary File S1.
